# Identifying what works in mental health apps through meta-regression analyses of 169 trials

**DOI:** 10.1038/s41746-026-02466-z

**Published:** 2026-03-11

**Authors:** Jannis Kraiss, Felix Fiß, Farid Chakhssi, Fatma Betül Aktas, Jurrijn Alexander Koelen, Jorge Piano Simões

**Affiliations:** 1 Department of Psychology, Health, and Technology, University of Twente, Enschede, the Netherlands; 2 Thubble, Dimence Groep, Deventer, the Netherlands; 3 Department of Guidance and Psychological Counseling, Ibn Haldun University, Istanbul, Turkey

**Keywords:** Diseases, Health care, Medical research, Psychology, Psychology

## Abstract

This meta-analysis aimed to code active cognitive behavioral elements in mental health apps and to examine the association between these elements and improvements in depression and anxiety. Trials evaluating mental health apps were coded based on 34 pre-registered elements. 169 trials with 1137 timepoints were included (*N* = 41,807; mean age = 34.3 years; 72.9% female). Psychoeducation, relaxation, mindfulness, and self-monitoring were used most frequently. Bivariate mixed-effect meta-regression models showed that many elements were weakly to moderately effective. Desensitization, stimulus control, and activity scheduling were most strongly and robustly associated with improvements in depression and exposure-based elements with improvements in anxiety. Ineffective elements included graded tasks and personal strengths, but in sum, there was considerable variation in the frequency and impact of active elements. Interventions incorporating a greater number of elements were more effective. This meta-analysis provides insight into how active elements in mental health apps are associated with therapeutic change, informing future interventions.

## Introduction

The widespread adoption of smartphones and the convenience they offer have positioned mental health applications (apps) as promising solutions to address the growing global mental health crisis, including challenges in mental health care access and a growing demand for services^1^. Mental health apps are highly scalable, allowing a large number of individuals to receive support^2,3^. Additionally, app-based interventions provide users with the discretion to seek care privately, reducing stigma, and the flexibility to engage with therapeutic tools anytime and anywhere^4–6^. Depressive and anxiety disorders, as well as subthreshold levels of these conditions, are among the most commonly experienced mental health issues worldwide and are associated with substantial economic costs, decreased quality of life, and disease burden^7,8^. Given the high prevalence and burden of these conditions and the significant gaps in mental health service availability^9–11^, mental health apps hold great potential for supporting people with these conditions and overcoming many barriers that limit access to care.

However, to realize this potential optimally, it is crucial to identify which features of mental health apps are most effective in addressing symptoms of depression and anxiety. Scrutinizing the evidence for mental health apps is essential, given that apps focusing on mental health are among the most widely used health apps^12,13^. Several meta-analyses have investigated the efficacy of app-based interventions on symptoms of depression and anxiety^14–18^. The most recent and comprehensive meta-analysis synthesized findings from 176 randomized controlled trials (RCTs) and reported small to moderate effect sizes for app-based interventions in improving symptoms of depression and anxiety^12^, indicating that app-based cognitive behavioral therapy (CBT) interventions are effective in addressing mental health challenges.

While this research indicates that apps can be effective, existing meta-analyses often lack a detailed analysis of specific *active elements* within mental health apps. Previous meta-analyses^14,15,19,20^, including the one by Linardon and colleagues^12^, categorized interventions using low-resolution labels such as “cognitive behavioral therapy” or “acceptance-based therapies”. We argue that it is essential to move beyond these broad categorizations and rather examine which specific active elements of app-based interventions are effective. Active elements are the procedures or techniques delivered by the app in order to produce therapeutic change^21^. The most established set of active elements stems from the treatment model of CBT and involves elements such as relaxation, exposure, or behavioral activation. In addition, active elements may be based on so-called “third-wave” CBT interventions^22^, including acceptance and commitment therapy (ACT)^23^, mindfulness-based cognitive therapy (MBCT)^24^, and dialectical behavior therapy (DBT)^25^. Examining active elements in a more granular manner is essential because interventions with the same label may employ different active elements or combinations thereof^21^. For example, two “CBT” interventions might differ significantly in the active elements they entail. Furthermore, interventions not labeled as “CBT” might still incorporate CBT active elements (e.g., an ACT intervention may integrate relaxation exercises). Complicating this matter, the provision of active elements in trials is not limited to experimental conditions, but comparator groups in trials may also integrate active elements. For example, a relaxation app may be employed in the comparison group. Ideally, meta-analyses should also consider the active elements provided in the comparator groups to derive more precise estimates regarding the efficacy of active elements.

Understanding the efficacy of specific active elements requires a higher-resolution approach than the common practice of labeling interventions into broad treatment models. This granular approach can be achieved by coding specific active elements (in both the experimental and comparator groups) based on theory-driven active elements, grounded in established treatment models. Manual coding of active elements has recently been used in health psychology to identify effective behavior change techniques, particularly in the context of smoking cessation^26,27^. However, in the field of digital mental health interventions, this approach remains novel. Applying it in this context could provide more granular insights into what active elements are effective in app-based mental health interventions, offering a clear advantage over traditional meta-analytic methods that rely on broader classifications.

The aim of the current study is to code active cognitive behavioral elements in randomized controlled trials evaluating the efficacy of mental health apps and meta-analytically examine the association between each active element and changes in symptoms of depression and anxiety.

## Results

### Study selection

**Fig. 1:**
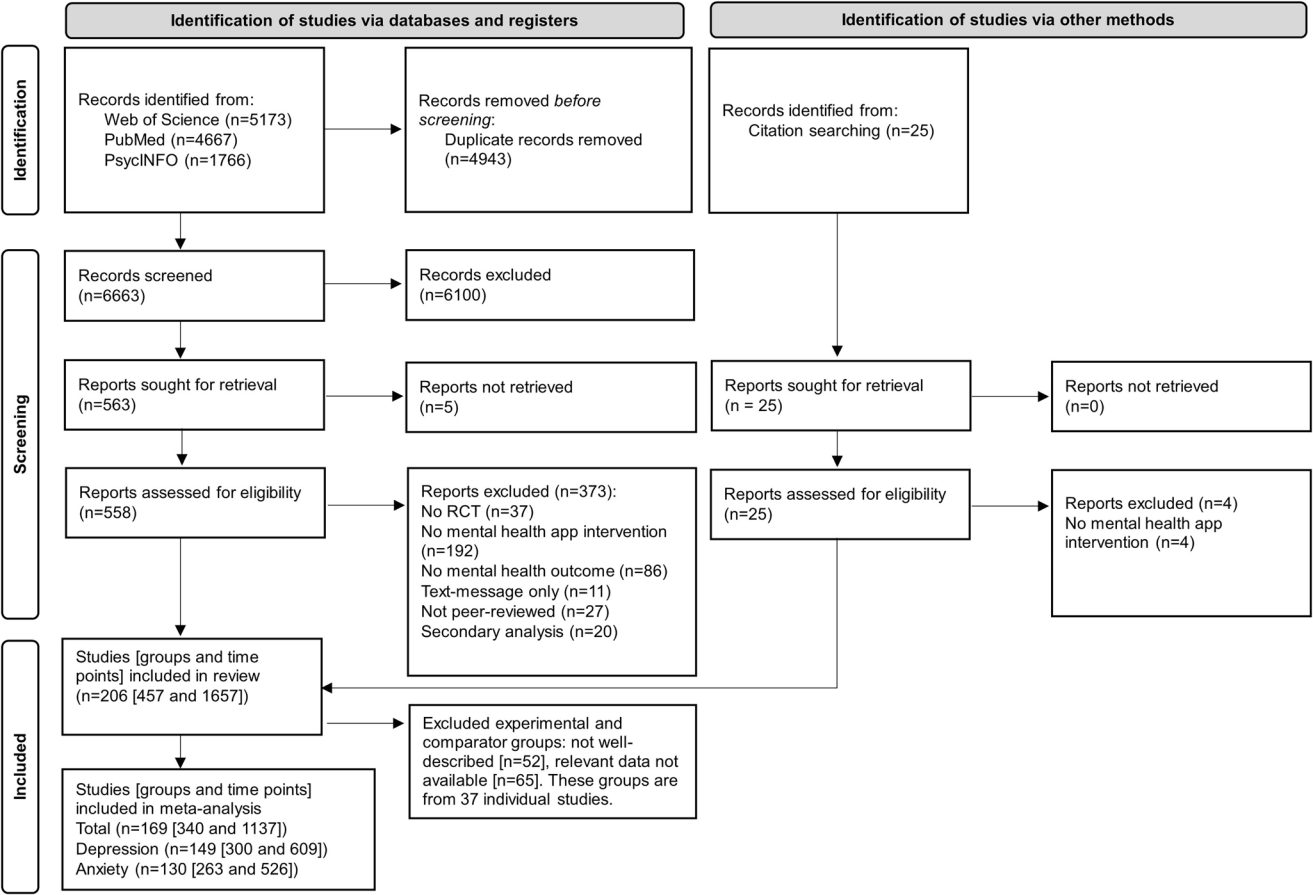
PRISMA flowchart of study selection. This figure shows the study selection process, including records and reports excluded at each stage and reasons for exclusion of full-text reports.

In total, 11,606 records were identified. After removal of duplicates and exclusion of titles and abstracts, 558 full texts were assessed for eligibility. Eventually, 206 papers were included, of which 25 papers were identified after additional citation searching. A reference list of all included trials and an overview of study characteristics can be found in the Supplementary Information(Tables S2 and S3). Finally, 38 individual trials and 118 groups were removed because the groups were not well-described (*n* = 51) or relevant information for the calculation of effect sizes was missing (*n* = 67). For the current meta-analysis, 169 trials with 340 groups and 1137 timepoints were included. Figure 1 shows the PRISMA flowchart of study selection.

### Study and sample characteristics

The 169 trials included in the analysis included 41,807 participants (weighted mean age = 34.3, 72.9% female), of which 20,704 were allocated to a control group and 21,103 to an experimental group. Of the 340 groups, 173 were experimental, and 167 were comparator groups. The average weighted dropout rate at post-intervention was 28.0% in the experimental and 23.7% in the comparator groups. Most trials were conducted in the United States (*n* = 51), Australia (*n* = 20), China (*n* = 14), and the United Kingdom (*n* = 12). Most trials included nonclinical (*n* = 71) or subclinical samples (*n* = 64), and most trials included samples without comorbidity (*n* = 155). The mental health app was an adjunctive treatment in only a few trials (*n* = 18). The average time to post-intervention was 6.02 weeks. Most experimental interventions were CBT (*n* = 55), multidisciplinary (*n* = 45), or third-wave (*n* = 45) interventions, while most control groups were waitlist (*n* = 60), usual care (*n* = 23), or 'no intervention' groups (*n* = 20).

### Active elements

**Fig. 2:**
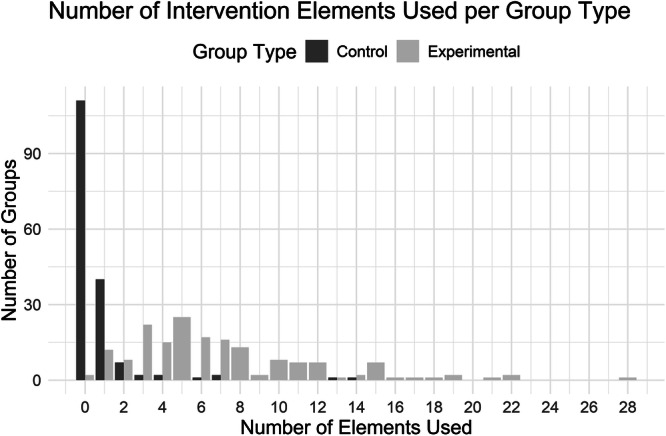
Frequency of active elements used in experimental and comparator groups. This figure shows the frequency of active elements used in experimental and comparator groups across all 169 trials included in the meta-regression analysis.

On average, groups used 3.82 (SD = 4.77) active elements. Experimental groups used 6.84 active elements (SD = 4.80, range 0–28). Two experimental groups used zero active elements, which is due to the fact that none of the 34 previously defined active elements occurred in these groups. These two groups comprised a body dissatisfaction app targeting avoidance tendencies and a simplified version of an ACT intervention that asked participants to regularly indicate whether their current behavior is directed towards or away from their values. Trials assessing symptoms of depression as treatment outcome used 6.76 active elements in experimental conditions (SD = 4.79, range 0–28), while trials assessing symptoms of anxiety used 6.69 active elements in experimental groups (SD = 4.83, range 0–22). On average, control groups used 0.69 active elements (SD = 1.80, range 0–14). Trials assessing symptoms of depression used 0.74 active elements in control groups (SD = 1.88, range 0–14), and trials assessing symptoms of anxiety used 0.47 active elements in control groups (SD = 0.92, range 0–7). Only 9.6% of the comparator groups used more than one active element, while 80.9% of experimental groups used between 0 and 10 active elements. Figure 2 shows the distribution of active elements separately for experimental and comparator groups.

Psychoeducation (*n* = 152, 44.7%), relaxation (*n* = 104, 30.6%), and mindfulness (*n* = 100, 29.4%) were the most frequently used active elements. When examining trials examining symptoms of depression or anxiety separately, these three were also the most frequent elements (Table 1). In experimental groups, the most frequently used active elements were also psychoeducation (*n* = 117, 67.6%), relaxation (*n* = 99, 57.2%), and mindfulness (*n* = 96, 55.5%). The most frequent active elements in control groups were psychoeducation (*n* = 35, 21%), self-monitoring (*n* = 21, 12.6%), goal-setting (*n* = 6, 3.6%), and problem-solving (*n* = 6, 3.6%).

**Table 1:** Frequency of active elements across groups included in the meta-analysis

Active element	Number of groups (%)		
	Total	Depression	Anxiety
Psychoeducation	152 (44.7)	134 (44.7)	115 (43.7)
Relaxation	104 (30.6)	94 (31.3)	83 (31.6)
Mindfulness	100 (29.4)	90 (30)	78 (29.7)
Self-monitoring	99 (29.1)	88 (29.3)	72 (27.4)
Present-moment focus	94 (27.6)	84 (28)	74 (28.1)
Cognitive restructuring	69 (20.3)	62 (20.7)	46 (17.5)
Behavioral activation	57 (16.8)	54 (18)	35 (13.3)
Problem-solving	53 (15.6)	47 (15.7)	39 (14.8)
Acceptance	52 (15.3)	45 (15)	39 (14.8)
Goal-setting	48 (14.1)	44 (14.7)	35 (13.3)
Self-compassion	42 (12.4)	35 (11.7)	35 (13.3)
Activity scheduling	36 (10.6)	35 (11.7)	20 (7.6)
Social skills	36 (10.6)	33 (11)	25 (9.5)
Cognitive defusion	33 (9.7)	28 (9.3)	28 (10.6)
Gratitude	33 (9.7)	28 (9.3)	26 (9.9)
Journaling	26 (7.7)	28 (9.3)	18 (6.8)
Mental imagery	26 (7.7)	25 (8.3)	22 (8.4)
Savoring	24 (7.1)	18 (6)	21 (8)
Values	24 (7.1)	22 (7.3)	18 (6.8)
Personal strengths	21 (6.2)	20 (6.7)	13 (4.9)
Behavior experiment	18 (5.3)	14 (4.7)	14 (5.3)
Exposure in vivo	18 (5.3)	11 (3.7)	14 (5.3)
Thought record	18 (5.3)	16 (5.3)	15 (5.7)
Functional analysis	16 (4.7)	12 (4)	12 (4.6)
Optimism	16 (4.7)	15 (5)	8 (3)
Externally-focused attention	14 (4.1)	10 (3.3)	12 (4.6)
Committed action	14 (4.1)	13 (4.3)	8 (3)
Self reinforcement	14 (4.1)	13 (4.3)	8 (3)
Stimulus control	11 (3.2)	10 (3.3)	10 (3.8)
Graded tasks	10 (2.9)	9 (3)	9 (3.4)
Interoceptive exposure	8 (2.4)	4 (1.3)	7 (2.7)
Desensitization	5 (1.5)	2 (0.7)	4 (1.5)
Imagery-based exposure	4 (1.2)	3 (1)	4 (1.5)
Worry exposure	4 (1.2)	4 (1.3)	3 (1.1)

Correlations showed weak to moderate correlations between active elements, suggesting weak to moderate co-occurrence of most combinations of active elements (Fig. S1).

### Association between active elements and changes in symptoms of depression

Findings from bivariate mixed-effect meta-regression models on the association between active elements and changes in symptoms of depression can be found in Table 2. Most active elements were found to be significantly associated with improvements in symptoms of depression. However, exposure in vivo and desensitization were not found to be associated with changes in symptoms of depression. Substantial differences in associations were found, ranging from elements strongly related to improvements in symptoms of depression, such as imagery-based exposure (*B* = −0.76, *p* < 0.001) or self-reinforcement (*B* = −0.42, *p* < 0.001), to elements less associated with improvements in symptoms, such as social skills (*B* = −0.22, *p* < 0.01) or self-compassion (*B* = −0.21, *p* < 0.01). *I*² values ranged from 10.7% to 20.3% (level 2) and from 78.6% to 88.2% (level 3), indicating that heterogeneity in effect sizes is largely explained by between-study differences Table 2.

**Table 2:** Results of bivariate mixed-effect meta-regression models for the association between individual active elements and improvements in symptoms of depression

Active element	Associations (*β*, 95% CI)	
	Complete data	Outliers excluded
Functional analysis	−0.31 (−0.55, −0.06)*	−0.11 (−0.32, 0.10)
Exposure in vivo	−0.22 (−0.46, 0.02)	−0.19 (−0.39, 0.01)
Imagery-based exposure	−0.76 (−1.20, −0.32)***	−0.25 (−0.67, 0.17)
Interoceptive exposure	−0.71 (−1.09, −0.33)***	−0.34 (−0.71, 0.02)
Desensitization	−0.44 (−0.92, 0.05)	−0.47 (−0.90, −0.05)*
Problem-solving	−0.30 (−0.41, −0.19)***	−0.25 (−0.34, −0.16)***
Goal-setting	−0.27 (−0.38, −0.15)***	−0.20 (−0.30, −0.09)***
Self-monitoring	−0.24 (−0.33, −0.15)***	−0.21 (−0.29, −0.13)***
Journaling	−0.38 (−0.55, −0.20)***	−0.27 (−0.42, −0.12)***
Cognitive restructuring	−0.29 (−0.38, −0.21)***	−0.25 (−0.33, −0.18)***
Thought record	−0.26 (−0.44, −0.08)**	−0.24 (−0.39, −0.08)**
Self-reinforcement	−0.42 (−0.62, −0.22)***	−0.29 (−0.47, −0.11)**
Social skills	−0.22 (−0.35, −0.08)**	−0.19 (−0.31, −0.08)***
Activity scheduling	−0.36 (−0.49, −0.23)***	−0.31 (−0.43, −0.20)***
Behavior experiment	−0.32 (−0.54, −0.10)**	−0.16 (−0.35, 0.03)
Self-compassion	−0.21 (−0.34, −0.08)**	−0.14 (−0.25, −0.03)*
Mental imagery	−0.27 (−0.41, −0.13)***	−0.19 (−0.31, −0.06)**
Worry exposure	−0.47 (−0.84, −0.11)*	−0.20 (−0.54, 0.15)
Relaxation	−0.30 (−0.36, −0.23)***	−0.27 (−0.33, −0.22)***
Behavior activation	−0.31 (−0.40, −0.22)***	−0.27 (−0.35, −0.18)***
Psychoeducation	−0.31 (−0.38, −0.23)***	−0.28 (−0.34, −0.21)***
Graded tasks	−0.30 (−0.60, −0.05)*	−0.10 (−0.33, 0.13)
Stimulus control	−0.39 (−0.62, −0.16)***	−0.37 (−0.57, −0.18)***
Externally-focused attention	−0.35 (−0.61, −0.08)*	−0.33 (−0.54, −0.11)**
Cognitive defusion	−0.27 (−0.41, −0.14)***	−0.26 (−0.38, −0.14)***
Values	−0.27 (−0.43, −0.11)***	−0.25 (−0.39, −0.11)***
Acceptance	−0.24 (−0.35, −0.13)***	−0.23 (−0.33, −0.14)***
Present-moment focus	−0.22 (−0.31, −0.14)***	−0.23 (−0.29, −0.16)***
Committed action	−0.27 (−0.49, −0.05)*	−0.28 (−0.47, −0.08)**
Mindfulness	−0.23 (−0.30, −0.15)***	−0.23 (−0.29, −0.16)***
Gratitude	−0.23 (−0.38, −0.09)**	−0.16 (−0.29, −0.04)**
Savoring	−0.31 (−0.49, −0.14)***	−0.20 (−0.35, −0.05)**
Optimism	−0.24 (−0.43, −0.04)*	−0.14 (−0.31, 0.03)
Personal strengths	−0.20 (−0.38, −0.03)*	−0.10 (−0.25, 0.05)
Timepoints (level 1)	609	591-595^a^
Groups (level 2)	300	296-297^a^
Trials (level 3)	149	148

*β* Standardized estimate, *CI* Confidence interval.

^a^Varying number of timepoints and groups, as the number of outliers excluded differed between active elements.

***p < 0.001, **p < 0.01, *p < 0.05.

Most of the estimates were relatively robust to the removal of outliers. However, some less frequently used active elements became statistically insignificant after removing outliers. These included functional analysis, imagery-based exposure, interoceptive exposure, graded tasks, optimism and gratitude. Desensitization became significant after removal of outliers (*B* = −0.22, *p* < 0.01). Conducting the analyses with covariates included did not substantially change the results, and no significant moderation was found with the source of coding for any of the active elements.

### Association between active elements and changes in symptoms of anxiety

For symptoms of anxiety (Table 3), exposure in vivo and desensitization were found to be significantly related to improvements in symptoms. However, many other elements that were significantly associated with improvements in symptoms of depression were not found to be significant, for example, journaling, self-reinforcement or social skills. *I*² values ranged from 54.2% to 74.6% (level 2) and from 22.8% to 42.9% (level 3), suggesting a larger proportion of within-study heterogeneity compared to the results on depression.

**Table 3:** Results of bivariate mixed-effect meta-regression models for the association between individual active elements and improvements in symptoms of anxiety

Active element	Associations (*B*, 95% CI)	
	Complete data	Outliers excluded
Functional analysis	−0.17 (−0.41, 0.07)	−0.18 (−0.36, −0.01)*
Exposure in vivo	−0.32 (−0.54, -0.10)**	−0.32 (−0.49, −0.15)***
Imagery-based exposure	−0.85 (−1.22, −0.48)***	−0.74 (−1.04, −0.43)***
Interoceptive exposure	−0.33 (−0.63, −0.02)*	−0.36 (−0.59, −0.13)**
Desensitization	−0.55 (−0.94, −0.15)**	−0.57 (−0.86, −0.28)***
Problem-solving	−0.25 (−0.38, −0.12)***	−0.28 (−0.38, −0.19)***
Goal-setting	−0.13 (−0.27, 0.01)	−0.16 (−0.27, −0.06)**
Self-monitoring	−0.18 (−0.29, −0.07)**	−0.19 (−0.27, −0.11)***
Journaling	−0.16 (−0.35, 0.04)	−0.20 (−0.34, −0.06)**
Cognitive restructuring	−0.20 (−0.33, -0.08)**	−0.20 (−0.29, −0.11)***
Thought record	−0.12 (−0.33, 0.09)**	−0.15 (−0.30, −0.00)*
Self-reinforcement	−0.18 (−0.46, 0.10)	−0.21 (−0.41, −0.01)*
Social skills	−0.14 (−0.31, 0.02)	−0.18 (−0.29, −0.06)**
Activity scheduling	−0.20 (−0.39, −0.02)*	−0.24 (−0.37, −0.11)***
Behavior experiment	−0.23 (−0.45, −0.01)*	−0.27 (−0.43, −0.10)**
Self-compassion	−0.19 (−0.33, −0.05)**	−0.23 (−0.33, −0.12)***
Mental imagery	−0.13 (−0.31, 0.04)	−0.17 (−0.30, −0.05)**
Worry exposure	−0.15 (−0.60, 0.30)	−0.11 (−0.45, 0.22)
Relaxation	−0.29 (−0.38, −0.20)***	−0.27 (−0.33, −0.21)***
Behavior activation	−0.35 (−0.49, −0.21)***	−0.25 (−0.35, −0.14)***
Psychoeducation	−0.30 (−0.39, −0.21)***	−0.25 (−0.31, −0.18)***
Graded tasks	−0.09 (−0.36, 0.19)	−0.13 (−0.32, 0.07)
Stimulus control	−0.33 (−0.58, −0.09)*	−0.30 (−0.49, −0.11)**
Externally-focused attention	−0.24 (−0.48, 0.00)	−0.28 (−0.45, −0.10)**
Cognitive defusion	−0.21 (−0.37, −0.06)**	−0.26 (−0.38, −0.15)***
Values	−0.13 (−0.32, −0.06)	−0.18 (−0.32, −0.04)*
Acceptance	−0.18 (−0.31, −0.04)**	−0.22 (−0.32, −0.13)***
Present-moment focus	−0.22 (−0.32, −0.12)***	−0.24 (−0.30, −0.17)***
Committed action	−0.19 (−0.48, −0.10)	−0.26 (−0.47, −0.04)*
Mindfulness	−0.21 (−0.31, −0.11)***	−0.23 (−0.29, −0.16)***
Gratitude	−0.08 (−0.24, 0.08)	−0.13 (−0.24, −0.01)*
Savoring	−0.11 (−0.29, 0.06)	−0.16 (−0.29, −0.03)*
Optimism	0.00 (−0.28, 0.29)	−0.04 (−0.24, 0.17)
Personal strengths	−0.05 (−0.28, 0.17)	−0.09 (−0.26, 0.07)
Timepoints (level 1)	526	508–511^a^
Groups (level 2)	263	258–260^a^
Trials (level 3)	130	130

*β* Standardized estimate, *CI* Confidence interval.

^a^Varying number of timepoints and groups, as the number of outliers excluded differed between active elements.

***p < 0.001, **p < 0.01, *p < 0.05.

Most of the estimates were relatively robust to the removal of outliers, but some of the active elements became statistically significant after removing outliers. These included functional analysis, goal-setting, journaling, self-reinforcement, social skills, mental imagery, externally-focused attention, values, committed action, gratitude, and savoring. The magnitude of the effect size did not substantially change in most cases. Conducting the analyses with covariates included did not substantially change the results. We did find a significant moderation with source of coding for exposure in vivo (*B* = 0.46, *p* < 0.05), but not for any of the other active elements.

### Moderation by clinical status

Problem-solving, values, and savoring were more strongly associated with improvements in symptoms of depression in clinical samples (problem-solving: *B* = −0.27, *p* < 0.05; values: *B* = −2.14, *p* < 0.001; savoring: *B* = −2.11, *p* < 0.001). Relaxation, behavioral activation, and psychoeducation were more strongly associated with improvements in symptoms of anxiety in clinical groups (relaxation: *B* = −0.44, *p* < 0.001; behavioral activation: *B* = −0.45, *p* < 0.05; psychoeducation: *B* = −0.32, *p* < 0.01).

### Association between the number of active elements and changes in symptoms

**Fig. 3:**
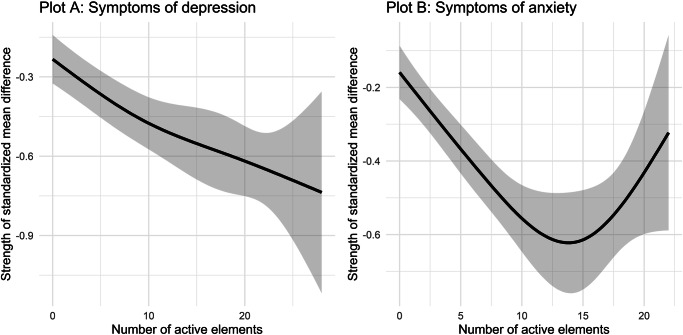
Cubic splines for the association between total number of active elements and improvements in symptoms of depression (Plot A) and anxiety (Plot B). This plot shows improvement in symptoms of depression or anxiety as a non-linear function of number of elements used in mental health apps. Improvement in symptoms is expressed as standardized within-group change from baseline to follow-up assessments.

We found that a higher number of active elements was significantly associated with improvements in symptoms of depression (*B* = −0.03, *p* < 0.001) and anxiety (*B* = −0.03, *p* < 0.001), suggesting that the more active elements an app integrated, the greater the improvement in depression and anxiety. As can be seen in Fig. 3, fitting cubic splines suggested that the relationship between the number of elements and improvements in depression was largely linear. However, for symptoms of anxiety, the cubic splines showed an U-shaped relationship, suggesting that in groups that utilized 15 or more active elements, interventions were becoming less effective. It should be noted though that for symptoms of anxiety, only 14 groups (5%) utilized 15 or more active elements. Hence, this result needs to be interpreted with caution.

### Risk of bias

The majority of the 169 trials (96%) had low risk for bias from in- and exclusion criteria, random sequence generation (79%) and blinding of outcome assessment (97%). Sixty-seven percent of trials reported an analysis approach adhering to intent-to-treat principles. However, only 39% of trials were scored with low risk for allocation concealment, 30% for blinding of participants or personnel, and 47% for risk of bias from drop-out. Only seven trials were scored with low risk for four or more items. A table with the risk of bias assessment per trial can be found in the Supplementary Information (Table S4) as well as a summary of risk of bias across all trials (Fig. S2).

## Discussion

This systematic review and meta-analysis coded active cognitive behavioral elements in randomized controlled trials evaluating the efficacy of mental health apps for improving symptoms of anxiety and depression. Its primary aim was to examine how each active element was associated with changes in symptoms. This meta-analysis is the first to examine the fine-grained impact of active elements in app-based mental health interventions targeting symptoms of depression and anxiety.

An interesting finding is the considerable variation in how frequently each active element was used in mental health apps. The most frequently used elements were psychoeducation, relaxation, mindfulness, and self-monitoring. In contrast, elements from ACT and positive psychology were used less frequently. This pattern is generally consistent with a recent review that found that ACT and positive psychology interventions were less commonly included in mental health apps^28^. Interestingly, that review reported that only about 2% of interventions explicitly claimed to use ACT, whereas we found that ACT elements were used much more frequently, for example, cognitive defusion was deployed in about 10% of interventions. This might suggest that ACT elements are often embedded within interventions, even if the intervention is not explicitly labeled as ACT-based. Broader consumer familiarity with CBT and mindfulness-based approaches may make developers more likely to label interventions explicitly as such, whereas ACT elements may be incorporated under more broader mindfulness or CBT labels. The frequencies of active elements observed in this study should be interpreted in light of potential underreporting, which is a common issue in trials on behavioral interventions^29,30^ and likely leads to an underestimation of the true prevalence of active elements. While coding interventions for this meta-analysis, we observed considerable variation in the quality of intervention reporting. We therefore urgently encourage authors of future trials to report active elements more consistently and thoroughly. This could be achieved, for example, by requiring authors to complete an intervention reporting checklist as a condition for publication in academic journals.

Most active elements were associated with improvements in depressive symptoms, also after excluding outliers. Many of the more frequently used active elements, such as problem-solving, cognitive restructuring, and behavioral activation, appear to have a relatively similar strength of association with improvements in depressive symptoms. This suggests that these elements are similarly effective and may therefore all be comparably effective when integrated into mental health apps. However, there might still be moderators of their efficacy, which we did not investigate in the current study. These may include other app features, such as guidance, or sample characteristics, such as age or gender. It could, for example, be that certain active elements are more effective in non-clinical or subthreshold groups, while others are more effective in clinical populations. Elements such as mood tracking may be particularly suited for individuals with milder symptoms, where increasing awareness and self-monitoring can play a preventive role or serve as early intervention. In contrast, more intensive elements like cognitive restructuring or exposure-based techniques may be necessary to produce meaningful change in clinical populations with higher symptom severity. However, the effectiveness of such intensive elements may also depend on the presence of therapeutic guidance, particularly in clinical populations. Our exploratory analyses by clinical status indeed suggest that some active elements are more effective in clinical groups. However, results from these analyses should be interpreted with caution, as the meta-analysis was not powered for this purpose, and as their lower impact in non-clinical and subclinical groups may be attributable to floor effects. Notably, the number of trials including clinical groups was relatively low in this meta-analysis, suggesting that the evidence base for these interventions in clinical populations remains limited. This may be due to ethical concerns around withholding standard treatment from clinical participants, challenges in recruiting individuals with more severe symptoms, or the additional resources required to conduct rigorous trials in clinical settings. Consequently, further research is needed to establish the effectiveness of specific active elements in these relevant groups.

It is striking that the strength of the effect sizes for many active elements is comparable to that found in the large meta-analysis by Linardon and colleagues^12^. They found an effect size of 0.28 across all trials using low-resolution labels. This overlap might be explained by the fact that the active elements (at least partially) co-occur in the apps. Therefore, the observed effect sizes in the current meta-analysis may partly reflect the influence of an underlying factor shared by these elements. This underlying factor might be “core” CBT, including elements such as relaxation, cognitive restructuring, and exposure, which are the most frequent active elements and are likely to be combined regularly within one intervention. A complementary hypothesis could be that engagement, including related factors such as gamification and personalization, might be a common factor in digital interventions and might eventually explain more of the effect than specific active elements^31^. It is also worth noting that there was substantial overlap in included studies with the meta-analysis by Linardon and colleagues, as 136 of the trials in our meta-analysis were also included in their study. This overlap might also partially explain the similarity in some of the observed effect sizes. Nevertheless, our fine-grained analysis complements and extends their work by identifying the specific intervention ingredients most strongly associated with symptom improvement. To better disentangle the efficacy of active elements, future studies might want to deploy factorial designs or micro-randomized trials by repeatedly assigning people to specific active elements and assessing their impact on mental health^32,33^. Future meta-analyses may also focus on identifying the best subset of active elements to find the most effective combination of elements.

Most of the third-wave active elements, including mindfulness and ACT elements, as well as positive psychology elements, were also significantly associated with improvements in symptoms of depression. This is generally in line with the efficacy of mindfulness-based interventions^34,35^, ACT^36^, and positive psychology^37^ in improving symptoms of depression. One finding that should be highlighted is that the effect size for mindfulness was comparable to the overall effect of mindfulness-based interventions reported in the previous meta-analysis by Linardon and colleauges^12^, who found an effect size of 0.26 across all mindfulness-based apps for improving depressive symptoms. Our approach still provides a more fine-grained estimate of the effect of mindfulness, since it also includes groups delivering mindfulness content that are not explicitly labeled as mindfulness interventions in the trial. This could be the case if mindfulness is integrated into a multidisciplinary intervention involving multiple different active elements. Nevertheless, this also highlights an important shortcoming of our coding scheme that deserves attention. Eventually, we were unable to code at least some of the active elements, such as mindfulness or gratitude, in a more fine-grained manner. For gratitude, for example, one could theoretically implement different types of gratitude exercises within one mental health app^38^. This limitation stems from the fact that there is a limited number of trials deploying these third-wave or positive psychology elements^28^, which restricts our ability to further differentiate between specific types or intensities of these active elements. This issue is further complicated by the lack of an established framework for active elements, not only for digital mental health interventions but also for mental health interventions in general. In health psychology, the taxonomy of behavior change techniques provides a reliable method for coding intervention content^39,40^, but this taxonomy was primarily developed in the context of health-related interventions, such as treatments targeting smoking cessation. In the context of mental health interventions, a comparable comprehensive taxonomy is currently lacking. To better understand what works in mental health interventions, future research could focus on the development of such a taxonomy tailored to mental health interventions. Furthermore, future meta-analyses or experimental studies could benefit from incorporating outcomes related to positive functioning and well-being^41^, rather than focusing solely on symptom reduction. It is possible that certain active elements primarily influence symptom-related outcomes, while others may have a greater impact on positive functioning and well-being.

Several exposure-based elements, such as imagery-based exposure and interoceptive exposure (but not exposure in vivo), were also associated with improvements in depressive symptoms. In some cases, such as for interoceptive exposure, the association is even considerably stronger than for other elements. Additionally, desensitization and stimulus control emerged as the most significant elements after excluding outliers, suggesting that they may be particularly effective in alleviating depressive symptoms. Traditionally, such exposure-based elements are effective in treating anxiety symptoms^42^, making this finding somewhat surprising. However, it is also important to note that imagery-based exposure, interoceptive exposure, and desensitization were only investigated in a small number of trials. This raises the possibility that their apparent efficacy may be driven by a few highly effective studies, rather than by the specific impact of the individual active elements themselves. We recommend interpreting the results of these less prevalent elements with caution, particularly when they no longer reach significance after the exclusion of outliers. Future research should prioritize (micro-)randomized trials that specifically isolate these promising but understudied techniques to confirm their efficacy.

It is interesting to note that most of the exposure-based elements were significantly associated with improvements in symptoms of anxiety, even after excluding outliers. These included exposure in vivo, imagery-based exposure, interoceptive exposure, and desensitization. This is relevant for two reasons. First, it suggests that exposure exercises delivered via apps are effective and could therefore be integrated into digital interventions. This has the potential to significantly reduce the burden on therapists if individuals can engage in these exercises independently^43^. The combination of exposure exercises with virtual reality could be especially promising in this context^44^. Second, it indicates that there may be a symptom-specific effect of exposure elements on anxiety, as we did not observe similarly robust effects for depressive symptoms after excluding outliers. This finding is important, as it supports the potential for greater personalization in mental health apps, depending on which symptoms the intervention aims to target.

We also found that many other active elements were associated with improvements in symptoms of anxiety, such as problem-solving, self-monitoring, and cognitive restructuring. Overall, these findings suggest that many elements that are effective in reducing depressive symptoms are also effective for anxiety. This is also in line with the meta-analysis by Linardon and colleagues, who found similar effects for anxiety as for symptoms of depression^12^, and it also aligns with the ongoing development of transdiagnostic treatment approaches, such as the Unified Protocol, which proposes that similar compositions of treatment elements may be equally effective across a range of mental disorders^45^. Clinically, these similar effects for depression and anxiety may also be explained by the strong symptomatic overlap between the two conditions, as well as their high degree of comorbidity^46^. While many of the active elements were significantly associated with improvements in symptoms of depression and anxiety, it is important to note that there are substantial differences in the magnitude of these associations. This contributes to our understanding by identifying which elements are more effective, thereby informing future research and guiding the development of apps to maximize effect sizes. Notably, as for symptoms of anxiety, more active elements became statistically significant after removing outliers, while the magnitude of the associations remained relatively unchanged. This is likely due to reduced heterogeneity in the models, which decreases the uncertainty of the estimates.

Another relevant finding is that app-based interventions incorporating a greater number of active elements tended to be more effective. This suggests that multicomponent apps may be particularly beneficial, likely because they offer a more comprehensive intervention package that addresses a broader range of problems. Additionally, including a wider variety of elements provides people with more options, which could enhance adherence and engagement, which remain challenging to enhance in contemporary digital interventions^47–49^. For symptoms of depression, this relationship appeared to be linear, whereas for anxiety, a U-shaped relationship was observed. This is interesting, as it might suggest a “sweet spot” in the number of active elements. Due to the relatively low number of trials that included a high number of active elements, the evidence in this meta-analysis remains limited and should be interpreted with caution. Nevertheless, it could be valuable for future research to compare different interventions with a varying number of active elements to examine whether such a sweet spot can be identified empirically.

Several limitations need to be considered when interpreting the findings. First, the coding of active elements was based on a framework that focused on a selected subset of elements. Some therapeutic models, such as DBT or schema therapy, are likely to be rare in app-based mental health interventions, since they often require intensive, individualized, and therapist-guided approaches. Therefore, the current framework does not represent an exhaustive list of active therapeutic elements. In this context, some elements could theoretically also be coded in a more fine-grained manner. For example, cognitive restructuring includes many different techniques. However, distinguishing different techniques remains challenging due to the limited number of trials and a lack of detailed reporting. Second, the coding of elements was partially conducted manually by a data extractor and was based on published intervention materials. This likely led to an underestimation of active elements, as underreporting is a common issue in behavioral intervention studies^29,30^. Third, the dosage of each active element remains unknown. It is reasonable to assume that the same element could be delivered with varying intensity across different apps, which is an important factor to consider in future analyses.

In sum, this meta-analysis is the first to examine the fine-grained impact of specific active elements on symptoms of depression and anxiety in app-based mental health interventions. We found substantial variation in the frequency with which different active elements were used, with the most common being psychoeducation, relaxation, mindfulness, and self-monitoring. Many active elements were significantly associated with reductions in depressive and anxiety symptoms. However, some elements appeared to primarily affect either depression or anxiety, and there were notable differences in the magnitude of these associations. In addition, we found that interventions incorporating a greater number of active elements tend to be more effective. Notably, we observed considerable variation in the quality of intervention reporting. We therefore urgently encourage authors of future trials to report active elements more consistently and thoroughly. Overall, this meta-analysis provides a foundation for optimizing digital mental health interventions by identifying which active elements are most effective. By pinpointing the components that may drive change, these findings offer concrete guidance for designing more targeted and impactful mental health apps.

## Methods

A systematic literature review and meta-analysis were conducted, which were pre-registered at PROSPERO (ID: CRD42025630092, registered January 17, 2025). The study protocol and extensive supporting information are available on OSF (https://osf.io/wc59a/). This article follows the Preferred Reporting Items for Systematic Reviews and Meta-Analyses (PRISMA)^50^. A completed PRISMA reporting checklist can be found in the Supplementary Information (Table S5).

### Eligibility criteria

Papers were included if (1) the study design was a randomized controlled trial, (2) the intervention was a smartphone-based mental health app, and (3) the trial included a validated instrument assessing symptoms of depression or anxiety. We defined smartphone-based mental health apps as mobile applications offered on smartphones designed to improve psychological distress or well-being, offering therapeutic techniques based on evidence-based treatment models, and potentially including features like self-management tools, data tracking, or educational resources. Smartphone-based mental health interventions were allowed to involve human guidance, could be combined with other technologies such as wearables, and may be offered in addition to face-to-face therapy. Trials that held an information session, an intake meeting, or a single session of psychoeducation before delivering the app program were included. Secondary analyses were included only if they provided additional findings not reported in the original paper. Moreover, smartphone-based interventions administered as adjunctive treatments (i.e., in combination with treatment as usual) were included, as long as the adjunctive smartphone-based intervention was primarily targeting mental health outcomes. Trials were excluded that evaluated interventions focusing on lifestyle behaviors (e.g., weight loss, diet), text-message interventions, or cognitive training (e.g., cognitive bias modification). Papers that were not peer-reviewed were excluded, as well as conference proceedings, abstracts, dissertations, and study protocols.

### Information sources

Relevant papers were identified through searches in Web of Science, PsycINFO (via EBSCO), and PubMed. PsycINFO and PubMed were chosen to cover both the fields of psychology and biomedical sciences, while Web of Science was included due to its interdisciplinary reach.

### Search strategy

The search string was adapted from the one deployed by Linardon and colleagues^12^ to ensure coverage of relevant terms like “smartphone”, “mental health” and “randomized controlled trials” and their variants. Search strings were adjusted to match the formatting requirements of each database (see review protocol for details, https://osf.io/vwgty). The search was conducted on April 30, 2024. Backward citation tracking was performed by examining the reference list of Linardon and colleagues^12^.

### Selection process

Two master’s students trained by the first and last author screened titles and abstracts of potentially eligible studies. A pilot screening of 750 records yielded a Cohen’s kappa of 0.72, indicating sufficient interrater reliability to proceed. When eligibility was uncertain, studies were included for full-text screening. Full-text articles were then assessed against predefined inclusion and exclusion criteria by three other trained master’s students. Initially, J.K. and F.F. independently screened 10% of the full texts to validate decisions and resolve discrepancies. Reviewers followed a standardized, hierarchical checklist, stopping the process when a study failed to meet any criterion. Remaining uncertainties during the screening of full texts were resolved through discussion.

### Data items

Data items that were extracted included study characteristics (e.g., first author’s last name, study year, country), sample characteristics (e.g., mean age, percentage of female participants), and treatment characteristics (e.g., length, presence of personalization or gamification features). For an overview of all data items, see the review protocol (https://osf.io/vwgty).

#### Active elements

The presence of 34 pre-registered active elements for each group of every trial was extracted. The active elements were derived from established frameworks, reviews, and trial protocols addressing CBT for depression^51–53^, anxiety^54–57^, and CBT in general^58,59^. In addition, active elements were integrated from “third-wave” CBT interventions^22^, including Acceptance and Commitment Therapy (ACT)^23^ and Mindfulness-Based Cognitive Therapy (MBCT)^24^. For ACT, these were cognitive defusion, values, acceptance, present-moment focus, and committed action^23,60^. Consistent with mindfulness-based protocols, “mindfulness” was also added as a distinct active element^61,62^. Finally, four active elements from the field of positive psychology were included that were aimed at enhancing positive behaviors and cognitions, namely gratitude, savoring, optimism, and personal strengths^63–65^. While these might not strictly fall under the umbrella of CBT, we still expected them to be relatively prevalent in mental health apps^28^. We also included them because we see them as complementary to CBT elements, whose focus is rather on dysfunctional cognitions or behaviors. In the Supplementary Information (Table S1), an overview and description of the 34 active elements can be found.

### Data extraction

Data extraction was conducted by four master’s students who received a training session from JK and JPS. During the training session, data from one example trial were extracted collaboratively. Afterwards, three papers were extracted independently by the four extractors and J.K. and J.P.S. and discussed inconsistencies and uncertainties. The four extractors then continued with data extraction, while J.K. provided feedback in cases of uncertainty.

Since many specific active elements were expected to be unreported in the published papers, a Qualtrics survey was created to gather additional information on whether specific active elements were used in each group of every trial. The corresponding author of each paper included in the review was contacted via email to indicate, for each of the 34 active elements, whether it was used in each group of the respective trial (coded as yes or no). In case information was missing for data extraction, this survey was sent together with the email in which the missing information was requested. After the initial request, two reminders were sent after two and four weeks. Deviating from the review protocol, a final step was added to the data extraction process in which one trained research assistant manually coded the presence of the 34 active elements for each group of every trial based on published materials. This was necessary due to the relatively low response rate to the Qualtrics survey (36.4%, *n* = 75 of contacted trial authors responded to the survey). In cases where trial groups were not adequately described to reliably extract active elements, the group was coded as not well-described. In addition, 70 of all trial authors were contacted whose papers lacked reporting of information relevant for data extraction and analyses (e.g., means, standard deviations, or sample characteristics), of which 39 (56%) responded with the requested information. Only those 70 authors whose papers lacked this information were contacted with the request to provide that additional information. In contrast, authors from all trials were invited to complete the Qualtrics survey to provide details on the active elements included in their interventions.

### Risk of bias

The risk of bias for all included RCTs was assessed using the Cochrane Collaboration Risk of Bias tool, with each criterion rated as “high“, “low”, or “insufficient information” ^66^. One trained rater conducted the assessment after receiving training. A senior rater (J.K.) independently evaluated 10% of the articles to compare results, and once a high level of agreement was confirmed, the trained rater proceeded to assess the remaining articles. Based on previous meta-analyses^12,67,68^, it was evaluated if (1) the inclusion and exclusion criteria were adequately described, (2) the method for generating the allocation sequence was truly random, and (3) the allocation sequence was concealed from those assigning participants to intervention groups. It was also examined if (4) participants and study personnel were sufficiently blinded to the assigned intervention, (5) outcome assessors were unaware of the intervention assignments (if only self-report measures were used, risk of bias was coded as “low”). Furthermore, (6) the completeness of outcome data was examined by verifying if an intention-to-treat analysis was performed (if there were no drop-outs, risk of bias was coded as “low”), and (7) risk of bias due to dropouts was evaluated, either through a description of the numbers and reasons, via dropout analysis, or by a flow chart that detailed the dropouts and reasons for dropout.

### Data analyses

Experimental and control groups that were not well-described were excluded from the analyses, as interventions that were not well-described are likely to lead to an underestimation of reported active elements. Sensitivity analyses were conducted with all studies, including trials that were not well-described, and we found that the findings of the primary meta-analyses did not substantially differ. Standardized mean differences were calculated by subtracting the average baseline score from the average post-measurement score, divided by the standard deviation of the difference^69^. This was done for each group within each trial and for each timepoint, resulting in time-varying within-group effect sizes nested within groups and trials. For the calculation of effect sizes, a correlation of 0.5 was assumed^70^, with sensitivity analyses suggesting no substantial differences when correlations of 0.3 or 0.7 were used. Hedge’s *g* correction was applied to account for bias due to small sample sizes.

For all analyses, a bivariate mixed-effects meta-regression model was used, which is implemented in R (version 4.3.2)^71^ in the *metafor* package^72^. The bivariate mixed-effects meta-regression model is a meta-analysis framework that allows for efficient modeling of active elements varying between trials and groups^26,27^. The nested structure of the data was accounted for by including random effects for timepoints nested within groups, and groups nested within trials. This approach utilizes effect sizes from all timepoints and groups, including comparator groups, thereby increasing statistical power and precision. Separate univariable models for each of the 34 active elements were run, in which the respective active element was entered as a fixed effect (coded as 0 or 1). Active elements were coded as varying between groups within and across trials. Since our approach relies on within-group changes over time instead of between-group effect sizes, the calculated standardized mean differences are not based on random assignment to groups. Therefore, as predefined in the study protocol, the following covariates were included in all models to control for potential confounding: age, gender, comorbidity, clinical severity, and length to follow-up. In addition, the variable adjunctive treatment was added as a post-hoc covariate. Furthermore, we conducted exploratory moderation analyses examining whether associations between active elements and clinical outcomes differed by clinical status. To this end, an interaction term between each active element and clinical status was included in all models. Moderation analyses were restricted to active elements that were present in at least 5% of intervention groups, as elements with lower prevalence were expected to yield poorly identified interaction effects. Clinical status was coded as non-clinical/subclinical (0) versus clinical (1). Descriptions of all covariates are provided in Table 4. Each model was run separately for symptoms of depression and anxiety.

**Table 4:** Overview of covariates included in bivariate mixed-effect meta-regression models

Variable	Description	Coding
Pre-defined covariates		
Age	Mean age of participants in the trial	Continuous
Gender	Percentage of female participants in the trial	Continuous
Comorbidity	If the population has a comorbidity with a physical or mental disorder (e.g., depressed patients being treated for cancer)	Categorical (0 = no, 1 = yes)
Clinical severity	Whether the sample was nonclinical, subclinical, or clinical. Subclinical: participants were screened for elevated levels of symptoms; Clinical: participants were selected after diagnosis was made by a healthcare professional	Categorical (0 = nonclinical, 1 = subclinical, 2 = clinical)
Length to follow-up	The time from baseline to a particular timepoint of the trial in weeks	Continuous
Post-hoc covariate		
Adjunctive intervention	Whether the smartphone-based mental health app was an adjunctive treatment	Categorical (0 = no, 1 = yes)

To examine whether results differed depending on the source of element coding, we conducted additional sensitivity analyses. Specifically, we reran all models, again restricted to active elements present in at least 5% of groups, adding an interaction term between the active element and coding source (0 = extractor-coded, 1 = author-reported via survey). In addition, outliers were identified by examining standardized residuals from the meta-regression model, by flagging timepoints with standardized residuals exceeding ±2.576 as potential outliers. These studies were visualized and removed in a sensitivity analysis to assess their impact on the results.

In an additional step, we examined whether the number of active elements used in each group is associated with changes in symptoms of depression and anxiety. For this purpose, the sum score was calculated reflecting the number of active elements in each group. We first examined this in a linear meta-regression model. However, because it could also be that the association between the number of elements and improvements in symptoms is nonlinear, we additionally fitted cubic spline models and plotted the association between the number of active elements and changes in symptoms as a nonlinear relationship. Syntax and outputs, including those of all sensitivity analyses, are available on OSF (https://osf.io/vwgty).

## Supplementary information

Supplementary Information

## Data Availability

The datasets generated and/or analyzed during the current study are not publicly available due to future publications but are available from the corresponding author upon reasonable request.

## Code availability

The underlying code for this study is available on OSF and can be accessed via this link: https://osf.io/wc59a/.
