# Supplementary material for: Identifying what works in mental health apps through meta-regression analyses of 169 trials
Source: NPJ Digit Med. 2026 Mar 11;9:336. doi: 10.1038/s41746-026-02466-z (PMC13111678; doi:10.1038/s41746-026-02466-z)
Supplement: Supplementary file 1 — Supplementary Information [file 41746_2026_2466_MOESM1_ESM.pdf]

Table S1. Description of active elements
Table S2. Reference list of all trials included in the systematic review (N=206)
Table S3. Sample and study characteristics of included studies (N=206)
Table S4. Risk of bias assessment on trial level
Figure S1. Correlation matrix
Figure S2. Risk of bias across all trials included in the systematic review
Table S5. PRISMA reporting checklist.

Table S1. Description of active elements

Intervention element	Description	References
Functional analysis	A technique in which antecedents (triggers, causes) and consequences of behavior are examined to identify the factors that maintain a problematic behavior.	Watkins et al., (2023)
Exposure in vivo	Confronting stimuli that are feared without showing avoidance behavior	Kaczkurkin et al. (2015)
Imagery-based exposure	Repeatedly visualizing feared or distressing situations to reduce emotional reactivity and maladaptive avoidance behaviors	Kaczkurkin et al. (2015)
Interoceptive exposure	Intentionally eliciting and confronting physical sensations associated with anxiety to decrease fear of those sensations	Kaczkurkin et al. (2015)
Desensitization	A gradual process of exposing individuals to feared stimuli or situations, often combined with relaxation techniques.	Kaczkurkin et al. (2015); Behar et al. (2009)
Problem solving	A structured approach to defining a problem, finding potential solutions, evaluating options, and implementing the best solution	Cuijpers & Kleiboer (2017); López-López et al. (2019);
Goal-setting	Defining specific, measurable, achievable, relevant, and time-bound (SMART) objectives to guide behavior and track progress	López-López et al. (2019)
Self-monitoring	Systematically recording and tracking one's behaviors, thoughts, or emotions, for example through daily diaries or mood charts	Newman et al. (2020); Behar et al. (2009)
Journaling	Writing down thoughts, emotions, and experiences to identify. May happen in a reflective or informal way.	Rice (2015); Kosasih et al. (2023)
Cognitive restructuring	Identifying and challenging irrational or maladaptive thoughts and replacing them with more constructive ones	Cuijpers & Kleiboer (2017); López-López et al. (2019); Rice (2015); Kaczkurkin et al. (2015); Clark et al. (2003); Behar et al. (2009)

Thought record	A cognitive-behavioral tool that helps individuals identify, evaluate, and reframe negative or distorted thoughts	Bennett-Levy (2003)
Self-reinforcement	Rewarding oneself for achieving specific goals or exhibiting desired behaviors	Rice (2015)
Social skills	Teaching and practicing strategies to improve interpersonal interactions, relationships, and communication. Assertiveness is a central part of this element.	Cuijpers & Kleiboer (2017); López-López et al. (2019)
Activity scheduling	Planning and scheduling activities that are likely to be enjoyable, meaningful or provide a sense of accomplishment	Rice (2015)
Behavior experiment	Testing out new behaviors or challenging beliefs in real-world situations	Bennett-Levy (2003); Clark et al. (2003)
Self-compassion	Fostering self-esteem and self-kindness in the face of difficulties or personal shortcomings	Watkins et al. (2023)
Mental imagery	Visualization techniques to create mental pictures of positive outcomes, desired behaviors, relaxing scenes, or safe spaces	Newman et al., (2020)
Worry exposure	A specific variant of imaginal exposure, in which cognitive avoidance is addressed by being imaginally exposed to the most feared outcome for a longer period	Kaczkurkin et al. (2015); McIntosh (2013)
Relaxation	A method to decrease physiological and psychological tension through techniques like deep breathing or progressive muscle relaxation	López-López et al. (2019); Newman et al. (2020); Rice (2015); Behar et al. (2009)
Behavioral activation	Encouraging individuals to engage in meaningful and enjoyable activities	Cuijpers & Kleiboer (2017); López-López et al. (2019)
Psychoeducation	The provision of information and resources to help individuals understand their mental health condition, its symptoms, and effective coping strategies	Cuijpers & Kleiboer (2017); López-López et al. (2019); Kaczkurkin et al. (2015)
Graded tasks	A strategy to break down complex or overwhelming goals into smaller, manageable steps	Rice (2015); Behar et al. (2009)
Stimulus control	A behavioral intervention that strengthens associations between specific cues and	Kaczkurkin et al. (2015); Behar et al. (2009)

	desired behaviors while weakening associations with interfering behaviors.	
Externally-focused attention	Direct attention to external stimuli in an adaptive way, allowing individuals to effectively engage with their environment and manage anxiety	Clarke et al. (2003)
Cognitive defusion	Learning to observe thoughts without attaching meaning or judgment to them	Hayes et al. (2006)
Values	Identifying what matters to an individual and using those values as a guide for behavior and decision-making	Hayes et al. (2006)
Acceptance	Embracing uncomfortable emotions, thoughts, and sensations without attempting to avoid or suppress them	Hayes et al. (2006)
Present-moment focus	Bringing attention to the current moment. It highlights awareness of the "here and now" to reduce distractions, rumination, or future-oriented worry.	Behar et al. (2009)
Committed action	Fostering value-driven actions despite the presence of obstacles or discomfort	Hayes et al. (2006)
Mindfulness	Paying non-judgmental attention to the present moment, observing thoughts, feelings, and bodily sensations with openness and acceptance	van Emmerik et al. (2017); Kabat-Zinn (1982)
Gratitude	Recognizing and appreciating the positive aspects of life	Ciarrochi et al. (2022); Carr et al. (2021); Geschwind et al. (2019)
Savoring	Fully enjoying and prolonging positive experiences.	Ciarrochi et al. (2022); Carr et al. (2021); Geschwind et al. (2019)
Optimism	Fostering a positive and hopeful outlook towards life and the future	Ciarrochi et al. (2022); Carr et al., (2021); Geschwind et al. (2019)

Table S2. Reference list of all trials included in the systematic review (N=206)

Abbasalizadeh, M., Farsi, Z., Sajadi, S. A., Atashi, A., & Fournier, A. (2024). The effect of resilience training with mHealth application based on micro-learning method on the stress and anxiety of nurses working in intensive care units: a randomized controlled trial. *BMC Medical Education*, *24*(1). https://doi.org/10.1186/s12909-024-05427-w

Abbott, D., Lack, C. W., & Anderson, P. (2023). Does using a mindfulness app reduce anxiety and worry? A randomized-controlled trial. *Journal of Cognitive Psychotherapy*, *37*(1), 26-42.

Aboody, D., Siev, J., & Doron, G. (2020). Building resilience to body image triggers using brief cognitive training on a mobile application: A randomized controlled trial. *Behaviour Research and Therapy*, *134*, 103723. https://doi.org/10.1016/j.brat.2020.10372

Abramovitch, A., Uwadiale, A., & Robinson, A. (2024). A randomized clinical trial of a gamified app for the treatment of perfectionism. *British Journal of Clinical Psychology*, *63*(1), 73–91. https://doi.org/10.1111/bjc.12444

Ahorsu, D. K., Lin, C., Imani, V., Carlbring, P., Nygårdh, A., Broström, A., Hamilton, K., & Pakpour, A. H. (2020). Testing an app-based intervention to improve insomnia in patients with epilepsy: A randomized controlled trial. *Epilepsy & Behavior*, *112*, 107371. https://doi.org/10.1016/j.yebeh.2020.107371

Akechi, T., Yamaguchi, T., Uchida, M., Imai, F., Momino, K., Katsuki, F., Sakurai, N., Miyaji, T., Mashiko, T., Horikoshi, M., Furukawa, T. A., Yoshimura, A., Ohno, S., Uehiro, N., Higaki, K., Hasegawa, Y., Akahane, K., Uchitomi, Y., & Iwata, H. (2023). Smartphone psychotherapy reduces fear of cancer recurrence among breast cancer survivors: a fully decentralized randomized controlled clinical trial (J-SUPPORT 1703 study). *Journal of Clinical Oncology*, *41*(5), 1069–1078. https://doi.org/10.1200/jco.22.00699

Al-Refae, M., Al-Refae, A., Munroe, M., Sardella, N. A., & Ferrari, M. (2021). A Self-Compassion and Mindfulness-Based Cognitive Mobile Intervention (SERENE) for depression, anxiety, and stress: promoting adaptive emotional regulation and wisdom. *Frontiers in Psychology*, *12*. https://doi.org/10.3389/fpsyg.2021.648087

Anastasiadou, D., Folkvord, F., Brugnera, A., Vinader, L. C., SerranoTroncoso, E., Jardí, C. C., Bertolin, R. L., Rodríguez, R. M., Nuñez, B. M., Berna, M. G., Torralbas-Ortega, J., Torrent-Solà, L., Puntí-Vidal, J., Ferrer, M. C., Domenjó, A. M., Marsa, M. D., Gunnard, K., Cusido, J., Cunillera, J. A., & Lupiañez-Villanueva, F. (2020). An mHealth intervention for the treatment of patients with an eating disorder: A multicenter randomized controlled trial. *International Journal of Eating Disorders*, *53*(7), 1120–1131. https://doi.org/10.1002/eat.23286

Araya, R., Menezes, P. R., Claro, H. G., Brandt, L. R., Daley, K. L., Quayle, J., Diez-Canseco, F., Peters, T. J., Cruz, D. V., Toyama, M., Aschar, S., Hidalgo-Padilla, L., Martins, H., Cavero, V., Rocha, T., Scotton, G., De Almeida Lopes, I. F., Begale, M., Mohr, D. C., & Miranda, J. J. (2021). Effect of a digital intervention on depressive symptoms in patients with comorbid hypertension or diabetes in Brazil and Peru. *JAMA*, *325*(18), 1852. https://doi.org/10.1001/jama.2021.4348

Bakker, D., Kazantzis, N., Rickwood, D., & Rickard, N. (2018). A randomized controlled trial of three smartphone apps for enhancing public mental health. *Behaviour Research and Therapy*, *109*, 75–83. https://doi.org/10.1016/j.brat.2018.08.003

Barroso, J., Madisetti, M., & Mueller, M. (2019). A feasibility study to develop and test a Cognitive Behavioral Stress Management mobile Health application for HIV-Related Fatigue. *Journal of Pain and Symptom Management*, *59*(2), 242–253. https://doi.org/10.1016/j.jpainsymman.2019.09.009

Bear, K. A., Barber, C. C., & Medvedev, O. N. (2022). The impact of a mindfulness app on postnatal distress. *Mindfulness*, *13*(11), 2765–2776. https://doi.org/10.1007/s12671-022-01992-7

Bell, I., Arnold, C., Gilbertson, T., D'Alfonso, S., Castagnini, E., Chen, N., Nicholas, J., O'Sullivan, S., Valentine, L., & Alvarez-Jimenez, M. (2023). A personalized, transdiagnostic smartphone intervention (MELLO) targeting repetitive negative thinking in young people with depression and anxiety: pilot randomized controlled trial. *Journal of Medical Internet Research*, *25*, e47860. https://doi.org/10.2196/47860

Ben-Zeev, D., Brian, R. M., Jonathan, G., Razzano, L., Pashka, N., Carpenter-Song, E., Drake, R. E., & Scherer, E. A. (2018). Mobile Health (MHealth) versus Clinic-Based Group Intervention for People with Serious Mental Illness: a randomized controlled trial. *Psychiatric Services*, *69*(9), 978–985. https://doi.org/10.1176/appi.ps.201800063

Ben-Zeev, D., Chander, A., Tauscher, J., Buck, B., Nepal, S., Campbell, A., & Doron, G. (2021). A smartphone intervention for people with serious mental illness: fully remote randomized controlled trial of CORE. *Journal of Medical Internet Research*, *23*(11). https://doi.org/10.2196/29201

Birney, A. J., Gunn, R., Russell, J. K., & Ary, D. V. (2016). MoodHacker Mobile web app with email for adults to Self-Manage Mild-to-Moderate Depression: Randomized Controlled trial. *JMIR Mhealth and Uhealth*, *4*(1). https://doi.org/10.2196/mhealth.4231

Birrell, L., Debenham, J., Furneaux-Bate, A., Prior, K., Spallek, S., Thornton, L., Chapman, C., & Newton, N. (2023). Evaluating a Peer-Support mobile app for mental health and substance use among adolescents over 12 months during the COVID-19 pandemic: randomized controlled trial. *Journal of Medical Internet Research*, *25*. https://doi.org/10.2196/45216

Boettcher, J., Magnusson, K., Marklund, A., Berglund, E., Blomdahl, R., Braun, U., Delin, L., Lundén, C., Sjöblom, K., Sommer, D., Von Weber, K., Andersson, G., & Carlbring, P. (2018). Adding a smartphone app to internet-based self-help for social anxiety: A randomized controlled trial. *Computers in Human Behavior*, *87*, 98–108. https://doi.org/10.1016/j.chb.2018.04.052

Borjalilu, S., Mazaheri, M. A., & Talebpour, A. (2019). Effectiveness of Mindfulness-Based stress management in the mental health of Iranian university students: a comparison of blended therapy, Face-to-Face sessions, and mHealth App (Aramgar). *Iranian Journal of Psychiatry and Behavioral Sciences*, *13*(2). https://doi.org/10.5812/ijpbs.84726

Børøsund, E., Ehlers, S. L., Clark, M. M., Andrykowski, M. A., Småstuen, M. C., & Nes, L. S. (2022). Digital stress management in cancer: Testing StressProffen in a 12-month randomized controlled trial. *Cancer*, *128*(7), 1503–1512. https://doi.org/10.1002/cncr.34046

Børøsund, E., Ehlers, S. L., Varsi, C., Clark, M. M., Andrykowski, M. A., Cvancarova, M., & Nes, L. S. (2020). Results from a randomized controlled trial testing StressProffen; an

application-based stress-management intervention for cancer survivors. *Cancer Medicine*, *9*(11), 3775–3785. https://doi.org/10.1002/cam4.3000
Bostock, S., Crosswell, A. D., Prather, A. A., & Steptoe, A. (2019). Mindfulness on-the-go: Effects of a mindfulness meditation app on work stress and well-being. *Journal of Occupational Health Psychology*, *24*(1), 127–138. https://doi.org/10.1037/ocp0000118
Bostock, S., Luik, A. I., & Espie, C. A. (2016). Sleep and Productivity Benefits of Digital Cognitive Behavioral therapy for insomnia. *Journal of Occupational and Environmental Medicine*, *58*(7), 683–689. https://doi.org/10.1097/jom.0000000000000778
Bröcker, E., Olff, M., Suliman, S., Kidd, M., Greyvenstein, L., & Seedat, S. (2024). A counsellor-supported 'PTSD Coach' intervention versus enhanced treatment as usual in a resource-constrained setting: A randomised controlled trial. *Cambridge Prisms Global Mental Health*, 1–25. https://doi.org/10.1017/gmh.2023.92
Bruehlman-Senecal, E., Hook, C. J., Pfeifer, J. H., FitzGerald, C., Davis, B., Delucchi, K. L., Haritatos, J., & Ramo, D. E. (2020). Smartphone app to address loneliness among college students: pilot randomized controlled trial. *JMIR Mental Health*, *7*(10), e21496. https://doi.org/10.2196/21496
Bruhns, A., Baumeister, A., Demeroutis, G., Jahn, H., Willenborg, B., Shaffy, A., Moritz, S., & Bücker, L. (2023a). A mobile-based aftercare intervention to increase self-esteem in inpatients diagnosed with depression: A randomized controlled trial. *Psychotherapy Research*, *33*(6), 783–802. https://doi.org/10.1080/10503307.2022.2157226
Bruhns, A., Baumeister, A., Demeroutis, G., Jahn, H., Willenborg, B., Shaffy, A., Moritz, S., & Bücker, L. (2023b). A mobile-based aftercare intervention to increase self-esteem in inpatients diagnosed with depression: A randomized controlled trial. *Psychotherapy Research*, *33*(6), 783–802. https://doi.org/10.1080/10503307.2022.2157226
Bruhns, A., Lüdtke, T., Moritz, S., & Bücker, L. (2021). A Mobile-Based intervention to increase self-esteem in students with depressive symptoms: randomized controlled trial. *JMIR Mhealth and Uhealth*, *9*(7), e26498. https://doi.org/10.2196/26498
Cardi, V., Meregalli, V., Di Rosa, E., Derrigo, R., Faustini, C., Keeler, J. L., Favaro, A., Treasure, J., & Lawrence, N. (2022). A community-based feasibility randomized controlled study to test food-specific inhibitory control training in people with disinhibited eating during COVID-19 in Italy. *Eating and Weight Disorders - Studies on Anorexia Bulimia and Obesity*, *27*(7), 2745–2757. https://doi.org/10.1007/s40519-022-01411-9
Carl, J. R., Miller, C. B., Henry, A. L., Davis, M. L., Stott, R., Smits, J. a. J., Emsley, R., Gu, J., Shin, O., Otto, M. W., Craske, M. G., Saunders, K. E. A., Goodwin, G. M., & Espie, C. A. (2020). Efficacy of digital cognitive behavioral therapy for moderate-to-severe symptoms of generalized anxiety disorder: A randomized controlled trial. *Depression and Anxiety*, *37*(12), 1168–1178. https://doi.org/10.1002/da.23079
Carli, V., Petros, N. G., Hadlaczky, G., Vitcheva, T., Berchialla, P., Bianchi, S., Carletto, S., Christinaki, E., Citi, L., Ottaviano, M., Ouakinin, S., Papastylianou, T., . . . Valenza, G. (2022). The NEVERMIND e-health system in the treatment of depressive symptoms among patients with severe somatic conditions: A multicentre, pragmatic randomised controlled trial. *EClinicalMedicine*, *48*, 101423. https://doi.org/10.1016/j.eclinm.2022.101423

Catuara-Solarz, S., Skorulski, B., Estella-Aguerri, I., Avella-Garcia, C. B., Shepherd, S., Stott, E., Hemmings, N. R., De Villa, A. R., Schulze, L., & Dix, S. (2022). The efficacy of "Foundations," a digital mental health app to improve mental well-being during COVID-19: Proof-of-Principle randomized controlled trial. *JMIR Mhealth and Uhealth*, *10*(7), e30976. https://doi.org/10.2196/30976

Cerea, S., Doron, G., Manoli, T., Patania, F., Bottesi, G., & Ghisi, M. (2022). Cognitive training via a mobile application to reduce some forms of body dissatisfaction in young females at high-risk for body image disorders: A randomized controlled trial. *Body Image*, *42*, 297–306. https://doi.org/10.1016/j.bodyim.2022.07.010

Cerea, S., Ghisi, M., Bottesi, G., Carraro, E., Broggio, D., & Doron, G. (2020). Reaching reliable change using short, daily, cognitive training exercises delivered on a mobile application: The case of Relationship Obsessive Compulsive Disorder (ROCD) symptoms and cognitions in a subclinical cohort. *Journal Of Affective Disorders*, *276*, 775–787. https://doi.org/10.1016/j.jad.2020.07.043

Chan, C. S., Wong, C. Y. F., Yu, B. Y. M., Hui, V. K. Y., Ho, F. Y. Y., & Cuijpers, P. (2021). Treating depression with a smartphone-delivered self-help cognitive behavioral therapy for insomnia: a parallel-group randomized controlled trial. *Psychological Medicine*, *53*(5), 1799–1813. https://doi.org/10.1017/s0033291721003421

Comtois, K. A., Mata-Greve, F., Johnson, M., Pullmann, M. D., Mosser, B., & Arean, P. (2022). Effectiveness of mental health apps for distress during COVID-19 in US Unemployed and Essential Workers: Remote Pragmatic Randomized clinical trial. *JMIR Mhealth and Uhealth*, *10*(11). https://doi.org/10.2196/41689

Cox, C. E., Hough, C. L., Jones, D. M., Ungar, A., Reagan, W., Key, M. D., Gremore, T., Olsen, M. K., Sanders, L., Greeson, J. M., & Porter, L. S. (2019). Effects of mindfulness training programmes delivered by a self-directed mobile app and by telephone compared with an education programme for survivors of critical illness: a pilot randomised clinical trial. *Thorax*, *74*(1), 33–42. https://doi.org/10.1136/thoraxjnl-2017-211264

Cox, C. E., Kelleher, S. A., Parish, A., Olsen, M. K., Bermejo, S., Dempsey, K., Jaggers, J., Hough, C. L., Moss, M., & Porter, L. S. (2023). Feasibility of Mobile App-based Coping Skills Training for Cardiorespiratory Failure Survivors: The Blueprint Pilot Randomized Controlled Trial. *Annals Of The American Thoracic Society*, *20*(6), 861–871. https://doi.org/10.1513/annalsats.202210-890oc

Dahne, J., Collado, A., Lejuez, C., Risco, C. M., Diaz, V. A., Coles, L., Kustanowitz, J., Zvolensky, M. J., & Carpenter, M. J. (2019). Pilot randomized controlled trial of a Spanish-language Behavioral Activation mobile app (¡Aptívate!) for the treatment of depressive symptoms among united states Latinx adults with limited English proficiency. *Journal of Affective Disorders*, *250*, 210–217. https://doi.org/10.1016/j.jad.2019.03.009

Dahne, J., Lejuez, C., Diaz, V. A., Player, M. S., Kustanowitz, J., Felton, J. W., & Carpenter, M. J. (2019). Pilot randomized trial of a Self-Help Behavioral Activation Mobile App for utilization in primary care. *Behavior Therapy*, *50*(4), 817–827. https://doi.org/10.1016/j.beth.2018.12.003

Danieli, M., Ciulli, T., Mousavi, S. M., Silvestri, G., Barbato, S., Di Natale, L., & Riccardi, G. (2022). Assessing the impact of conversational artificial intelligence in the treatment

of stress and anxiety in aging Adults: randomized controlled trial. *JMIR Mental Health*, *9*(9), e38067. https://doi.org/10.2196/38067

De Kock, J. H., Latham, H. A., Cowden, R. G., Cullen, B., Narzisi, K., Jerdan, S., Munoz, S., Leslie, S. J., Stamatis, A., & Eze, J. (2022). Brief digital interventions to support the psychological well-being of NHS staff during the COVID-19 pandemic: 3-ARM Pilot randomized controlled trial. *JMIR Mental Health*, *9*(4). https://doi.org/10.2196/34002

Deady, M., Collins, D. a. J., Lavender, I., Mackinnon, A., Glozier, N., Bryant, R., Christensen, H., & Harvey, S. B. (2023). Selective prevention of depression in workers using a smartphone app: randomized controlled trial. *Journal of Medical Internet Research*, *25*, e45963. https://doi.org/10.2196/45963

Deady, M., Glozier, N., Calvo, R., Johnston, D., Mackinnon, A., Milne, D., Choi, I., Gayed, A., Peters, D., Bryant, R., Christensen, H., & Harvey, S. B. (2022). Preventing depression using a smartphone app: a randomized controlled trial. *Psychological Medicine*, *52*(3), 457–466. https://doi.org/10.1017/s0033291720002081

Depp, C. A., Ceglowski, J., Wang, V. C., Yaghouti, F., Mausbach, B. T., Thompson, W. K., & Granholm, E. L. (2015). Augmenting psychoeducation with a mobile intervention for bipolar disorder: A randomized controlled trial. *Journal Of Affective Disorders*, *174*, 23–30. https://doi.org/10.1016/j.jad.2014.10.053

DiNardo, M. M., Greco, C., Phares, A. D., Beyer, N. M., Youk, A. O., Obrosky, D. S., Morone, N. E., Owen, J. E., Saba, S. K., Suss, S. J., & Siminerio, L. (2022). Effects of an integrated mindfulness intervention for veterans with diabetes distress: a randomized controlled trial. *BMJ Open Diabetes Research & Care*, *10*(2). https://doi.org/10.1136/bmjdrc-2021-002631

Dingwall, K. M., Sweet, M., Cass, A., Hughes, J. T., Kavanagh, D., Howard, K., Barzi, F., Brown, S., Sajiv, C., Majoni, S. W., & Nagel, T. (2021). Effectiveness of Wellbeing Intervention for Chronic Kidney Disease (WICKD): results of a randomised controlled trial. *BMC Nephrology*, *22*(1). https://doi.org/10.1186/s12882-021-02344-8

Ditton, E., Knott, B., Hodyl, N., Horton, G., Oldmeadow, C., Walker, F. R., & Nilsson, M. (2023). Evaluation of an APP-Delivered Psychological Flexibility Skill Training Intervention for medical student burnout and Well-being: randomized controlled trial. *JMIR Mental Health*, *10*. https://doi.org/10.2196/42566

Domar, A. D., Jasulaitis, L., Matevossian, K., Jasulaitis, S., Grill, E. A., & Meike, L. U. (2023). The impact of the FertiStrong mobile application on anxiety and depression in Men: a randomised control pilot study. *Journal of Human Reproductive Sciences*, *16*(3), 195–203. https://doi.org/10.4103/jhrs.jhrs_75_23

Donker, T., Fehribach, J., Van Klaveren, C., Cornelisz, I., Toffolo, M. B. J., Van Straten, A., & Van Gelder, J. (2022). Automated mobile virtual reality cognitive behavior therapy for aviophobia in a natural setting: a randomized controlled trial. *Psychological Medicine*, *53*(13), 6232–6241. https://doi.org/10.1017/s0033291722003531

Donker, T., Cornelisz, I., Van Klaveren, C., Van Straten, A., Carlbring, P., Cuijpers, P., & Van Gelder, J. (2019). Effectiveness of Self-guided App-Based Virtual Reality Cognitive Behavior Therapy for Acrophobia: a randomized clinical trial. *JAMA Psychiatry*, *76*(7), 682. https://doi.org/10.1001/jamapsychiatry.2019.0219

Economides, M., Bolton, H., Male, R., & Cavanagh, K. (2021). Feasibility and preliminary efficacy of Web-Based and mobile interventions for common mental health problems

in working adults: Multi-Arm randomized pilot trial. *JMIR Formative Research*, *6*(3), e34032. https://doi.org/10.2196/34032

El-Jawahri, A., Luskin, M. R., Greer, J. A., Traeger, L., Lavoie, M., Vaughn, D. M., Andrews, S., Yang, D., Boateng, K. Y., Newcomb, R. A., Ufere, N. N., Fathi, A. T., Hobbs, G., Brunner, A., Abel, G. A., Stone, R. M., DeAngelo, D. J., Wadleigh, M., & Temel, J. S. (2023). Psychological mobile app for patients with acute myeloid leukemia: A pilot randomized clinical trial. *Cancer*, *129*(7), 1075–1084. https://doi.org/10.1002/cncr.34645

Everitt, N., Broadbent, J., Richardson, B., Smyth, J. M., Heron, K., Teague, S., & Fuller-Tyszkiewicz, M. (2021). Exploring the features of an app-based just-in-time intervention for depression. *Journal Of Affective Disorders, 291*, 279–287. https://doi.org/10.1016/j.jad.2021.05.021

Faurholt-Jepsen, M., Tønning, M. L., Fros, M., Martiny, K., Tuxen, N., Rosenberg, N., Busk, J., Winther, O., Thaysen-Petersen, D., Aamund, K. A., Tolderlund, L., Bardram, J. E., & Kessing, L. V. (2021). Reducing the rate of psychiatric re-admissions in bipolar disorder using smartphones—The RADMIS trial. *Acta Psychiatrica Scandinavica*, *143*(5), 453–465. https://doi.org/10.1111/acps.13274

Fiol-DeRoque, M. A., Serrano-Ripoll, M. J., Jiménez, R., Zamanillo-Campos, R., Yáñez-Juan, A. M., Bennasar-Veny, M., Leiva, A., Gervilla, E., García-Buades, M. E., García-Toro, M., Alonso-Coello, P., Pastor-Moreno, G., Ruiz-Pérez, I., Sitges, C., García-Campayo, J., Llobera-Cánaves, J., & Ricci-Cabello, I. (2021). A Mobile Phone–Based Intervention to reduce mental health problems in health care workers during the COVID-19 pandemic (PsyCOvidApp): randomized controlled trial. *JMIR Mhealth and Uhealth*, *9*(5), e27039. https://doi.org/10.2196/27039

Fish, M. T., & Saul, A. D. (2019). The Gamification of Meditation: A Randomized-Controlled Study of a Prescribed Mobile Mindfulness Meditation Application in Reducing College Students' Depression. *Simulation & Gaming*, *50*(4), 419–435. https://doi.org/10.1177/1046878119851821

Fitzpatrick, K. K., Darcy, A., & Vierhile, M. (2017). Delivering Cognitive Behavior Therapy to Young Adults With Symptoms of Depression and Anxiety Using a Fully Automated Conversational Agent (Woebot): A Randomized Controlled Trial. *JMIR Mental Health*, *4*(2), e19. https://doi.org/10.2196/mental.7785

Flett, J. a. M., Hayne, H., Riordan, B. C., Thompson, L. M., & Conner, T. S. (2019). Mobile Mindfulness Meditation: a Randomised Controlled Trial of the Effect of Two Popular Apps on Mental Health. *Mindfulness*, *10*(5), 863–876. https://doi.org/10.1007/s12671-018-1050-9

Forman-Hoffman, V. L., Sihvonen, S., Wielgosz, J., Kuhn, E., Nelson, B. W., Peiper, N. C., & Gould, C. E. (2024). Therapist-supported digital mental health intervention for depressive symptoms: A randomized clinical trial. *Journal of Affective Disorders*, *349*, 494–501. https://doi.org/10.1016/j.jad.2024.01.057

Fuller-Tyszkiewicz, M., Richardson, B., Little, K., Teague, S., Hartley-Clark, L., Capic, T., Khor, S., Cummins, R. A., Olsson, C. A., & Hutchinson, D. (2020). Efficacy of a smartphone app intervention for reducing caregiver stress: randomized controlled trial. *JMIR Mental Health*, *7*(7), e17541. https://doi.org/10.2196/17541

Gao, M., Roy, A., Deluty, A., Sharkey, K. M., Hoge, E. A., Liu, T., & Brewer, J. A. (2022). Targeting Anxiety to Improve Sleep Disturbance: A Randomized Clinical Trial of App-Based Mindfulness Training. *Psychosomatic Medicine*, *84*(5), 632–642. https://doi.org/10.1097/psy.0000000000001083

Gao, Y., Shi, L., Fu, N., Yang, N., Weeks-Gariepy, T., & Mao, Y. (2024). Mobile-delivered mindfulness intervention on Anxiety Level among College Athletes: A Randomized Controlled Trial (Preprint). *Journal Of Medical Internet Research*, *26*, e40406. https://doi.org/10.2196/40406

Gee, B. L., Batterham, P. J., Gulliver, A., Reynolds, J., & Griffiths, K. M. (2021). An Ecological Momentary Intervention for people with social anxiety: A descriptive case study. *Informatics For Health And Social Care*, *46*(4), 370–398. https://doi.org/10.1080/17538157.2021.1896525

Ghaemi, S. N., Sverdlov, O., Van Dam, J., Campellone, T., & Gerwien, R. (2022). A Smartphone-Based Intervention as an adjunct to Standard-of-Care Treatment for schizophrenia: randomized controlled trial. *JMIR Formative Research*, *6*(3). https://doi.org/10.2196/29154

Ghanbari, E., Yektatalab, S., & Mehrabi, M. (2021). Effects of psychoeducational interventions using mobile apps and Mobile-Based Online group Discussions on Anxiety and Self-Esteem in Women with Breast Cancer: randomized controlled trial. *JMIR Mhealth and Uhealth*, *9*(5). https://doi.org/10.2196/19262

Gnanapragasam, S. N., Tinch-Taylor, R., Scott, H. R., Hegarty, S., Souliou, E., Bhundia, R., Lamb, D., Weston, D., Greenberg, N., Madan, I., Stevelink, S., Raine, R., Carter, B., & Wessely, S. (2023). Multicentre, England-wide randomised controlled trial of the 'Foundations' smartphone application in improving mental health and well-being in a healthcare worker population. *The British Journal of Psychiatry*, *222*(2), 58–66. https://doi.org/10.1192/bjp.2022.10

Goldberg, S. B., Imhoff-Smith, T., Bolt, D. M., Wilson-Mendenhall, C. D., Dahl, C. J., Davidson, R. J., & Rosenkranz, M. A. (2020). Testing the Efficacy of a Multicomponent, Self-Guided, Smartphone-Based Meditation App: Three-Armed Randomized Controlled Trial. *JMIR Mental Health*, *7*(11), e23825. https://doi.org/10.2196/23825

Graham, A. K., Greene, C. J., Kwasny, M. J., Kaiser, S. M., Lieponis, P., Powell, T., & Mohr, D. C. (2020). Coached mobile app platform for the treatment of depression and anxiety among primary care patients. *JAMA Psychiatry*, *77*(9), 906. https://doi.org/10.1001/jamapsychiatry.2020.1011

Greer, J. A., Jacobs, J., Pensak, N., MacDonald, J. J., Fuh, C., Perez, G. K., Ward, A., Tallen, C., Muzikansky, A., Traeger, L., Penedo, F. J., El-Jawahri, A., Safren, S. A., Pirl, W. F., & Temel, J. S. (2019). Randomized Trial of a Tailored Cognitive-Behavioral Therapy Mobile Application for Anxiety in Patients with Incurable Cancer. *The Oncologist*, *24*(8), 1111–1120. https://doi.org/10.1634/theoncologist.2018-0536

Grubbs, K. M., Abraham, T. H., Pyne, J. M., Greene, C. J., Teng, E. J., & Fortney, J. C. (2022). Enhancing Problem-Solving therapy with smartphone Technology: a pilot randomized controlled trial. *Psychiatric Services*, *73*(7), 805–808. https://doi.org/10.1176/appi.ps.201900254

Guo, Y., Hong, Y. A., Cai, W., Li, L., Hao, Y., Qiao, J., Xu, Z., Zhang, H., Zeng, C., Liu, C., Li, Y., Zhu, M., Zeng, Y., & Penedo, F. J. (2020). Effect of a WeChat-Based intervention

(Run4Love) on depressive symptoms among people living with HIV in China: a randomized controlled trial. *Journal of Medical Internet Research*, *22*(2), e16715. https://doi.org/10.2196/16715

Ha, S. W., & Kim, J. (2020). Designing a Scalable, Accessible, and Effective Mobile App Based Solution for Common Mental Health Problems. *International Journal Of Human-Computer Interaction*, *36*(14), 1354–1367. https://doi.org/10.1080/10447318.2020.1750792

Ham, K., Chin, S., Suh, Y. J., Rhee, M., Yu, E., Lee, H. J., Kim, J., Kim, S. W., Koh, S., & Chung, K. (2019). Preliminary Results From a Randomized Controlled Study for an App-Based Cognitive Behavioral Therapy Program for Depression and Anxiety in Cancer Patients. *Frontiers in Psychology*, *10*. https://doi.org/10.3389/fpsyg.2019.01592

Hanssen, E., Balvert, S., Oorschot, M., Borkelmans, K., Van Os, J., Delespaul, P., & Fett, A. (2020). An ecological momentary intervention incorporating personalised feedback to improve symptoms and social functioning in schizophrenia spectrum disorders. *Psychiatry Research*, *284*, 112695. https://doi.org/10.1016/j.psychres.2019.112695

He, Y., Yang, L., Zhu, X., Wu, B., Zhang, S., Qian, C., & Tian, T. (2022). Mental health chatbot for young adults with depressive symptoms during the COVID-19 pandemic: Single-Blind, Three-Arm randomized controlled trial. *Journal of Medical Internet Research*, *24*(11), e40719. https://doi.org/10.2196/40719

Heim, E., Ramia, J. A., Hana, R. A., Burchert, S., Carswell, K., Cornelisz, I., Cuijpers, P., Chammay, R. E., Noun, P., Van Klaveren, C., Van Ommeren, M., Zoghbi, E., & Hof, E. V. (2021). Step-by-step: Feasibility randomised controlled trial of a mobile-based intervention for depression among populations affected by adversity in Lebanon. *Internet Interventions*, *24*, 100380. https://doi.org/10.1016/j.invent.2021.100380

Hensler, I., Sveen, J., Cernvall, M., & Arnberg, F. K. (2022). Efficacy, Benefits, and Harms of a Self-management App in a Swedish Trauma-Exposed Community Sample (PTSD Coach): Randomized Controlled Trial. *Journal Of Medical Internet Research*, *24*(3), e31419. https://doi.org/10.2196/31419

Hilt, L. M., Swords, C. M., & Webb, C. A. (2023). Randomized controlled trial of a mindfulness mobile application for ruminative adolescents. *Journal of Clinical Child & Adolescent Psychology*, 1–14. https://doi.org/10.1080/15374416.2022.2158840

Hirshberg, M. J., Frye, C., Dahl, C. J., Riordan, K. M., Vack, N. J., Sachs, J., Goldman, R., Davidson, R. J., & Goldberg, S. B. (2022). A randomized controlled trial of a smartphone-based well-being training in public school system employees during the COVID-19 pandemic. *Journal of Educational Psychology*, *114*(8), 1895–1911. https://doi.org/10.1037/edu0000739

Horsch, C. H., Lancee, J., Griffioen-Both, F., Spruit, S., Fitrianie, S., Neerincx, M. A., Beun, R. J., & Brinkman, W. (2017). Mobile Phone-Delivered Cognitive Behavioral Therapy for Insomnia: A Randomized Waitlist Controlled Trial. *Journal Of Medical Internet Research*, *19*(4), e70. https://doi.org/10.2196/jmir.6524

Howells, A., Ivtzan, I., & Eiroa-Orosa, F. J. (2016). Putting the ‘app’ in Happiness: A Randomised Controlled Trial of a Smartphone-Based Mindfulness Intervention to Enhance Wellbeing. *Journal of Happiness Studies*, *17*(1), 163–185. https://doi.org/10.1007/s10902-014-9589-1

Huberty, J. L., Espel-Huynh, H. M., Neher, T. L., & Puzia, M. E. (2022). Testing the Pragmatic Effectiveness of a Consumer-Based Mindfulness Mobile App in the Workplace: Randomized controlled trial. *JMIR Mhealth and Uhealth*, *10*(9). https://doi.org/10.2196/38903

Huberty, J., Puzia, M. E., Green, J., Vlisides-Henry, R. D., Larkey, L., Irwin, M. R., & Vranceanu, A. (2021). A mindfulness meditation mobile app improves depression and anxiety in adults with sleep disturbance: Analysis from a randomized controlled trial. *General Hospital Psychiatry*, *73*, 30–37. https://doi.org/10.1016/j.genhosppsych.2021.09.004

Hunt, M., Miguez, S., Dukas, B., Onwude, O., & White, S. (2021). Efficacy of Zemedy, a Mobile Digital Therapeutic for the Self-management of Irritable Bowel Syndrome: Crossover Randomized Controlled Trial. *JMIR Mhealth And Uhealth*, *9*(5), e26152. https://doi.org/10.2196/26152

Hur, J., Kim, B., Park, D., & Choi, S. (2018). A Scenario-Based Cognitive Behavioral Therapy Mobile App to Reduce Dysfunctional Beliefs in Individuals with Depression: A Randomized Controlled Trial. *Telemedicine Journal And e-Health*, *24*(9), 710–716. https://doi.org/10.1089/tmj.2017.0214

Hwang, H., Kim, S. M., Netterstrøm, B., & Han, D. H. (2022). The Efficacy of a Smartphone-Based App on Stress Reduction: Randomized Controlled trial. *Journal of Medical Internet Research*, *24*(2). https://doi.org/10.2196/28703

Hwang, W. J., & Jo, H. H. (2019). Evaluation of the effectiveness of Mobile App-Based Stress-Management Program: a randomized controlled trial. *International Journal of Environmental Research and Public Health*, *16*(21), 4270. https://doi.org/10.3390/ijerph16214270

Imamura, K., Tran, T. T. T., Nguyen, H. T., Sasaki, N., Kuribayashi, K., Sakuraya, A., Bui, T. M., Nguyen, A. Q., Nguyen, Q. T., Nguyen, N. T., Nguyen, K. T., Nguyen, G. T. H., Tran, X. T. N., Truong, T. Q., Zhang, M. W., Minas, H., Sekiya, Y., Watanabe, K., Tsutsumi, A., & Kawakami, N. (2021). Effect of smartphone-based stress management programs on depression and anxiety of hospital nurses in Vietnam: a three-arm randomized controlled trial. *Scientific Reports*, *11*(1). https://doi.org/10.1038/s41598-021-90320-5

Jannati, N., Mazhari, S., Ahmadian, L., & Mirzaee, M. (2020). Effectiveness of an app-based cognitive behavioral therapy program for postpartum depression in primary care: A randomized controlled trial. *International Journal Of Medical Informatics*, *141*, 104145. https://doi.org/10.1016/j.ijmedinf.2020.104145

Järvelä-Reijonen, E., Puttonen, S., Karhunen, L., Sairanen, E., Laitinen, J., Kolehmainen, M., Pihlajamäki, J., Kujala, U. M., Korpela, R., Ermes, M., Lappalainen, R., & Kolehmainen, M. (2020). The Effects of Acceptance and Commitment Therapy (ACT) Intervention on Inflammation and Stress Biomarkers: a Randomized Controlled Trial. *International Journal of Behavioral Medicine*, *27*(5), 539–555. https://doi.org/10.1007/s12529-020-09891-8

Kauer, S. D., Reid, S. C., Crooke, A. H. D., Khor, A., Hearps, S. J. C., Jorm, A. F., Sanci, L., & Patton, G. (2012). Self-monitoring Using Mobile Phones in the Early Stages of Adolescent Depression: Randomized Controlled Trial. *Journal Of Medical Internet Research*, *14*(3), e67. https://doi.org/10.2196/jmir.1858

Keng, S., Chin, J. W. E., Mammadova, M., & Teo, I. (2022). Effects of Mobile App-Based Mindfulness Practice on Healthcare Workers: a Randomized Active Controlled Trial. *Mindfulness*, *13*(11), 2691–2704. https://doi.org/10.1007/s12671-022-01975-8

Kenny, R., Fitzgerald, A., Segurado, R., & Dooley, B. (2019). Is there an app for that? A cluster randomised controlled trial of a mobile app–based mental health intervention. *Health Informatics Journal*, *26*(3), 1538–1559. https://doi.org/10.1177/1460458219884195

Kerber, A., Beintner, I., Burchert, S., & Knaevelsrud, C. (2023). Effects of a Self-Guided Transdiagnostic Smartphone App on Patient Empowerment and Mental Health: Randomized Controlled Trial. *JMIR Mental Health*, *10*, e45068. https://doi.org/10.2196/45068

Kim, K., Hwang, H., Bae, S., Kim, S. M., & Han, D. H. (2024). The effectiveness of digital app for reduction of clinical symptoms in individuals with panic disorder: a randomized controlled trial (Preprint). *Journal of Medical Internet Research*, *26*. https://doi.org/10.2196/51428

Kirykowicz, K., Jaworski, B., Owen, J., Kirschbaum, C., Seedat, S., & Van Den Heuvel, L. L. (2023). Feasibility, acceptability and preliminary efficacy of a mental health self-management app in clinicians working during the COVID-19 pandemic: A pilot randomised controlled trial. *Psychiatry Research*, *329*, 115493. https://doi.org/10.1016/j.psychres.2023.115493

Kloos, N., Austin, J., Van 'T Klooster, J., Drossaert, C., & Bohlmeijer, E. (2022). Appreciating the good things in life during the COVID-19 pandemic: a randomized controlled trial and evaluation of a gratitude app. *Journal of Happiness Studies*, *23*(8), 4001–4025. https://doi.org/10.1007/s10902-022-00586-3

Kollei, I., Lukas, C. A., Loeber, S., & Berking, M. (2017). An app-based blended intervention to reduce body dissatisfaction: A randomized controlled pilot study. *Journal Of Consulting And Clinical Psychology*, *85*(11), 1104–1108. https://doi.org/10.1037/ccp0000246

Kosasih, F. R., Yee, V. T. S., Toh, S. H. Y., & Sündermann, O. (2023). Efficacy of Intellect's self-guided anxiety and worry mobile health programme: A randomized controlled trial with an active control and a 2-week follow-up. *PLOS Digital Health*, *2*(5), e0000095. https://doi.org/10.1371/journal.pdig.0000095

Krafft, J., Potts, S., Schoendorff, B., & , M. E. (2019). A Randomized Controlled Trial of Multiple Versions of an Acceptance and Commitment Therapy Matrix App for Well-Being. *Behavior Modification*, *43*(2), 246–272. https://doi.org/10.1177/0145445517748561

Kubo, A., Kurtovich, E., McGinnis, M., Aghaee, S., Altschuler, A., Quesenberry, C., Kolevska, T., & Avins, A. L. (2019). A randomized controlled trial of MHealth mindfulness intervention for cancer patients and informal cancer caregivers: a feasibility study within an integrated health care delivery system. *Integrative Cancer Therapies*, *18*. https://doi.org/10.1177/1534735419850634

Kubo, A., Kurtovich, E., McGinnis, M., Aghaee, S., Altschuler, A., Quesenberry, C., Kolevska, T., Liu, R., Greyz-Yusupov, N., & Avins, A. (2024). Pilot pragmatic randomized trial of mHealth mindfulness-based intervention for advanced cancer patients and their informal caregivers. *Psycho-Oncology*, *33*(2). https://doi.org/10.1002/pon.5557

Kuhn, E., Kanuri, N., Hoffman, J. E., Garvert, D. W., Ruzek, J. I., & Taylor, C. B. (2017). A randomized controlled trial of a smartphone app for posttraumatic stress disorder

symptoms. *Journal of Consulting and Clinical Psychology*, *85*(3), 267–273. https://doi.org/10.1037/ccp0000163

Kuhn, E., Miller, K. E., Puran, D., Wielgosz, J., YorkWilliams, S. L., Owen, J. E., Jaworski, B. K., Hallenbeck, H. W., McCaslin, S. E., & Taylor, K. L. (2022). A pilot randomized controlled trial of the Insomnia Coach Mobile app to assess its feasibility, acceptability, and potential efficacy. *Behavior Therapy*, *53*(3), 440–457. https://doi.org/10.1016/j.beth.2021.11.003

Kulikov, V. N., Crosthwaite, P. C., Hall, S. A., Flannery, J. E., Strauss, G. S., Vierra, E. M., Koepsell, X. L., Lake, J. I., & Padmanabhan, A. (2023). A CBT-based mobile intervention as an adjunct treatment for adolescents with symptoms of depression: a virtual randomized controlled feasibility trial. *Frontiers in Digital Health*, *5*. https://doi.org/10.3389/fdgth.2023.1062471

Kusumadewi, A. F., Marchira, C. R., Widyandana, D., & Wirasto, R. T. (2023). Randomized Clinical Trial on the Comparison of Effect of Asynchronous Mobile Application and Guided Brief Cognitive Behavioral Therapy in Managing Anxiety among Medical Students. *Trends in Psychiatry and Psychotherapy*. https://doi.org/10.47626/2237-6089-2023-0713

Lacey, C., Frampton, C., & Beaglehole, B. (2023). oVRcome – Self-guided virtual reality for specific phobias: A randomised controlled trial. *Australian & New Zealand Journal Of Psychiatry*, *57*(5), 736–744. https://doi.org/10.1177/00048674221110779

LaFreniere, L. S., & Newman, M. G. (2023). Upregulating positive emotion in generalized anxiety disorder: A randomized controlled trial of the SkillJoy ecological momentary intervention. *Journal of Consulting and Clinical Psychology*, *91*(6), 381–387. https://doi.org/10.1037/ccp0000794

Lahtinen, O., Aaltonen, J., Kaakinen, J., Franklin, L., & Hyönä, J. (2023). The effects of app-based mindfulness practice on the well-being of university students and staff. *Current Psychology*, *42*(6), 4412–4421. https://doi.org/10.1007/s12144-021-01762-z

Laird, B., Puzia, M., Larkey, L., Ehlers, D., & Huberty, J. (2022). Feasibility of using a mobile app for stress in Middle-Aged men and Women. (Preprint). *JMIR Formative Research*, *6*(5). https://doi.org/10.2196/30294

Lee, R. A., & Jung, M. E. (2018). Evaluation of an mHealth App (DeStressify) on University Students' Mental Health: Pilot Trial. *JMIR Mental Health*, *5*(1), e2. https://doi.org/10.2196/mental.8324

Lee, Y. J. (2023). Effects of a mobile health intervention on activities of stress self-management for workers. *Work*, *75*(1), 233–241. https://doi.org/10.3233/wor-211406

Levin, M. E., Haeger, J., & Cruz, R. A. (2019). Tailoring Acceptance and Commitment Therapy Skill Coaching in the Moment Through Smartphones: Results from a Randomized Controlled Trial. *Mindfulness*, *10*(4), 689–699. https://doi.org/10.1007/s12671-018-1004-2

Levin, M. E., Haeger, J., An, W., & Twohig, M. P. (2018). Comparing Cognitive Defusion and Cognitive Restructuring Delivered Through a Mobile App for Individuals High in Self-Criticism. *Cognitive Therapy And Research*, *42*(6), 844–855. https://doi.org/10.1007/s10608-018-9944-3

Levin, M. E., Hicks, E. T., & Krafft, J. (2020). Pilot evaluation of the stop, breathe & think mindfulness app for student clients on a college counseling center waitlist. *Journal Of*

American College Health, *70*(1), 165–173. https://doi.org/10.1080/07448481.2020.1728281

Lewis, S., Ainsworth, J., Sanders, C., Stockton-Powdrell, C., Machin, M., Whelan, P., Hopkins, R., He, Z., Applegate, E., Drake, R., Bamford, C., Roberts, C., & Wykes, T. (2020). Smartphone-Enhanced Symptom Management in Psychosis: open, randomized controlled trial. *Journal of Medical Internet Research*, *22*(8). https://doi.org/10.2196/17019

Li, A. C., Wong, K. K., Chio, F. H., Mak, W. W., & Poon, L. W. (2022). Delivering Mindfulness-Based interventions for insomnia, pain, and dysfunctional eating through a text messaging app: Three randomized controlled trials investigating the effectiveness and mediating mechanisms. *Journal of Medical Internet Research*, *24*(5). https://doi.org/10.2196/30073

Li, Y., Guo, Y., Hong, Y. A., Zhu, M., Zeng, C., Qiao, J., Xu, Z., Zhang, H., Zeng, Y., Cai, W., Li, L., & Liu, C. (2019). Mechanisms and Effects of a WeChat-Based Intervention on Suicide Among People Living With HIV and Depression: Path Model Analysis of a Randomized Controlled Trial. *Journal Of Medical Internet Research*, *21*(11), e14729. https://doi.org/10.2196/14729

Li, Y., Rhee, H., Bullock, L. F. C., McCaw, B., & Bloom, T. (2024). Self-Compassion, Health, and Empowerment: A pilot randomized controlled trial for Chinese immigrant women experiencing intimate partner violence. *Journal of Interpersonal Violence*, *39*(7–8), 1571–1595. https://doi.org/10.1177/08862605231207624

Lim, Y. S., Quek, J. H., Ching, X. W., Lim, D. T. R., Lim, K. G., Thuraisingham, C., & George, P. P. (2023). Efficacy of a Text-Based Mental Health Coaching App in Improving the Symptoms of Stress, Anxiety, and Depression: Randomized Controlled Trial. *JMIR Formative Research*, *7*, e46458. https://doi.org/10.2196/46458

Linardon, J., Shatte, A., McClure, Z., & Fuller-Tyszkiewicz, M. (2023). A broad v. focused digital intervention for recurrent binge eating: a randomized controlled non-inferiority trial. *Psychological Medicine*, *53*(10), 4580–4591. https://doi.org/10.1017/s0033291722001477

Linardon, J., Shatte, A., Rosato, J., & Fuller-Tyszkiewicz, M. (2022). Efficacy of a transdiagnostic cognitive-behavioral intervention for eating disorder psychopathology delivered through a smartphone app: a randomized controlled trial. *Psychological Medicine*, *52*(9), 1679–1690. https://doi.org/10.1017/s0033291720003426

Litvin, S., Saunders, R., Jefferies, P., Seely, H., Pössel, P., & Lüttke, S. (2023). The Impact of a Gamified Mobile Mental Health App (eQuoo) on Resilience and Mental Health in a Student Population: Large-Scale Randomized Controlled Trial. *JMIR Mental Health*, *10*, e47285. https://doi.org/10.2196/47285

Litvin, S., Saunders, R., Maier, M. A., & Lüttke, S. (2020). Gamification as an approach to improve resilience and reduce attrition in mobile mental health interventions: A randomized controlled trial. *PLoS ONE*, *15*(9), e0237220. https://doi.org/10.1371/journal.pone.0237220

Liu, C., Chen, H., Zhou, F., Long, Q., Wu, K., Lo, L., Hung, T., Liu, C., & Chiou, W. (2022). Positive intervention effect of mobile health application based on mindfulness and social support theory on postpartum depression symptoms of puerperae. *BMC Women S Health*, *22*(1). https://doi.org/10.1186/s12905-022-01996-4

Liu, H., Peng, H., Song, X., Xu, C., & Zhang, M. (2022). Using AI chatbots to provide self-help depression interventions for university students: A randomized trial of effectiveness. *Internet Interventions*, *27*, 100495. https://doi.org/10.1016/j.invent.2022.100495

Liu, Y., Hasimu, M., Jia, M., Tang, J., Wang, Y., He, X., Yan, T., Xie, S., & Li, K. (2023). The Effects of APP-Based Intervention for Depression Among Community-Dwelling Individuals With Spinal Cord Injury: A Randomized Controlled Trial. *Archives Of Physical Medicine And Rehabilitation*, *104*(2), 195–202. https://doi.org/10.1016/j.apmr.2022.10.005

Lu, Y., Li, Y., Huang, Y., Zhang, X., Wang, J., Wu, L., & Cao, F. (2023). Effects and Mechanisms of a Web- and Mobile-Based Acceptance and Commitment Therapy Intervention for Anxiety and Depression Symptoms in Nurses: Fully Decentralized Randomized Controlled Trial. *Journal Of Medical Internet Research*, *25*, e51549. https://doi.org/10.2196/51549

Luangapichart, P., Saisavoey, N., & Viravan, N. (2022). Efficacy and Feasibility of the Minimal Therapist-Guided Four-Week Online Audio-Based mindfulness Program 'Mindful Senses' for Burnout and Stress Reduction in Medical Personnel: a randomized controlled trial. *Healthcare*, *10*(12), 2532. https://doi.org/10.3390/healthcare10122532

Lüdtke, T., Pult, L. K., Schröder, J., Moritz, S., & Bücker, L. (2018). A randomized controlled trial on a smartphone self-help application (Be Good to Yourself) to reduce depressive symptoms. *Psychiatry Research*, *269*, 753–762. https://doi.org/10.1016/j.psychres.2018.08.113

Luo, Y., Xia, W., Cheung, A. T., Ho, L. L. K., Zhang, J., Xie, J., Xiao, P., & Li, H. C. W. (2021). Effectiveness of a Mobile Device–Based Resilience Training Program in Reducing Depressive Symptoms and Enhancing Resilience and Quality of Life in Parents of Children With Cancer: Randomized Controlled Trial. *Journal Of Medical Internet Research*, *23*(11), e27639. https://doi.org/10.2196/27639

MacKinnon, A. L., Simpson, K. M., Salisbury, M. R., Bobula, J., Penner-Goeke, L., Berard, L., Rioux, C., Giesbrecht, G. F., Giuliano, R., Lebel, C., Protudjer, J. L. P., Reynolds, K., Sauer-Zavala, S., Soderstrom, M., Tomfohr-Madsen, L. M., & Roos, L. E. (2022). Building Emotional Awareness and Mental Health (BEAM): a pilot randomized controlled trial of an App-Based program for mothers of toddlers. *Frontiers in Psychiatry*, *13*. https://doi.org/10.3389/fpsyt.2022.880972

Mak, W. W., Tong, A. C., Yip, S. Y., Lui, W. W., Chio, F. H., Chan, A. T., & Wong, C. C. (2018). Efficacy and Moderation of Mobile App–Based Programs for Mindfulness-Based Training, Self-Compassion Training, and Cognitive Behavioral Psychoeducation on Mental Health: Randomized Controlled Noninferiority Trial. *JMIR Mental Health*, *5*(4), e60. https://doi.org/10.2196/mental.8597

Mantani, A., Kato, T., Furukawa, T. A., Horikoshi, M., Imai, H., Hiroe, T., Chino, B., Funayama, T., Yonemoto, N., Zhou, Q., & Kawanishi, N. (2017). Smartphone Cognitive Behavioral Therapy as an adjunct to pharmacotherapy for refractory Depression: Randomized Controlled trial. *Journal of Medical Internet Research*, *19*(11), e373. https://doi.org/10.2196/jmir.8602

Marciniak, M. A., Shanahan, L., Myin-Germeys, I., Veer, I. M., Yuen, K. S. L., Binder, H., Walter, H., Hermans, E. J., Kalisch, R., & Kleim, B. (2023). Imager—A mobile health mental

imagery-based ecological momentary intervention targeting reward sensitivity: A randomized controlled trial. *Applied Psychology Health And Well-Being*, *16*(2), 576–596. https://doi.org/10.1111/aphw.12505

McCloud, T., Jones, R., Lewis, G., Bell, V., & Tsakanikos, E. (2020). Effectiveness of a mobile app intervention for anxiety and depression symptoms in university students: randomized controlled trial. *JMIR Mhealth and Uhealth*, *8*(7). https://doi.org/10.2196/15418

McGillivray, L., Hui, N. K., Wong, Q. J. J., Han, J., Qian, J., & Torok, M. (2023). The Effectiveness of a Smartphone Intervention Targeting Suicidal Ideation in Young Adults: Randomized Controlled Trial Examining the Influence of Loneliness. *JMIR Mental Health*, *10*, e44862. https://doi.org/10.2196/44862

Miklowitz, D. J., Weintraub, M. J., Ichinose, M. C., Denenny, D. M., Walshaw, P. D., Wilkerson, C. A., Frey, S. J., Morgan-Fleming, G. M., Brown, R. D., Merranko, J. A., & Arevian, A. C. (2023). A randomized clinical trial of Technology-Enhanced Family-Focused therapy for youth in the early stages of mood disorders. *JAACAP Open*, *1*(2), 93–104. https://doi.org/10.1016/j.jaacop.2023.04.002

Min, B., Park, H., Kim, J. I., Lee, S., Back, S., Lee, E., Oh, S., Yun, J., Kim, B., Kim, Y., Hwang, J., Lee, S., & Kim, J. (2023). The effectiveness of a Neurofeedback-Assisted mindfulness training program using a mobile app on stress reduction in employees: randomized controlled trial. *JMIR Mhealth and Uhealth*, *11*, e42851. https://doi.org/10.2196/42851

Mistretta, E. G., Davis, M. C., Temkit, M., Lorenz, C., Darby, B., & Stonnington, C. M. (2018). Resilience Training for Work-Related Stress among Health care Workers. *Journal of Occupational and Environmental Medicine*, *60*(6), 559–568. https://doi.org/10.1097/jom.0000000000001285

Moberg, C., Niles, A., & Beermann, D. (2019). Guided Self-Help Works: Randomized Waitlist Controlled Trial of Pacifica, a Mobile App Integrating Cognitive Behavioral Therapy and Mindfulness for Stress, Anxiety, and Depression. *Journal Of Medical Internet Research*, *21*(6), e12556. https://doi.org/10.2196/12556

Moritz, S., Grudzień, D. P., Gawęda, Ł., Aleksandrowicz, A., Balzan, R., Shaffy, A., Bruhns, A., Borsutzky, S. M., & Rolvien, L. (2024). A randomized controlled trial on COGITO, a free self-help smartphone app to enhance mental well-being. *Journal of Psychiatric Research*, *174*, 254–257. https://doi.org/10.1016/j.jpsychires.2024.04.021

Mutter, A., Küchler, A., Idrees, A. R., Kählke, F., Terhorst, Y., & Baumeister, H. (2023). StudiCare procrastination - Randomized controlled non-inferiority trial of a persuasive design-optimized internet- and mobile-based intervention with digital coach targeting procrastination in college students. *BMC Psychology*, *11*(1). https://doi.org/10.1186/s40359-023-01312-1

Newman, M. G., Jacobson, N. C., Rackoff, G. N., Bell, M. J., & Taylor, C. B. (2021). A randomized controlled trial of a smartphone-based application for the treatment of anxiety. *Psychotherapy Research*, *31*(4), 443–454. https://doi.org/10.1080/10503307.2020.1790688

Nicol, G., Wang, R., Graham, S., Dodd, S., & Garbutt, J. (2022). Chatbot-Delivered Cognitive Behavioral therapy in adolescents with depression and Anxiety during the COVID-19 Pandemic: Feasibility and Acceptability study. *JMIR Formative Research*, 6(11).https://doi.org/10.2196/40242

Nishi, D., Imamura, K., Watanabe, K., Obikane, E., Sasaki, N., Yasuma, N., Sekiya, Y., Matsuyama, Y., & Kawakami, N. (2022). The preventive effect of internet-based cognitive behavioral therapy for prevention of depression during pregnancy and in the postpartum period (iPDP): a large scale randomized controlled trial. *Psychiatry And Clinical Neurosciences, 76*(11), 570–578. https://doi.org/10.1111/pcn.13458
Nishi, D., Imamura, K., Watanabe, K., Obikane, E., Sasaki, N., Yasuma, N., Sekiya, Y., Matsuyama, Y., & Kawakami, N. (2022). The preventive effect of internet-based cognitive behavioral therapy for prevention of depression during pregnancy and in the postpartum period (iPDP): a large scale randomized controlled trial. *Psychiatry And Clinical Neurosciences*, *76*(11), 570–578. https://doi.org/10.1111/pcn.13458
O'Dea, B., Han, J., Batterham, P. J., Achilles, M. R., Calear, A. L., Werner-Seidler, A., Parker, B., Shand, F., & Christensen, H. (2020). A randomised controlled trial of a relationship-focussed mobile phone application for improving adolescents' mental health. *Journal of Child Psychology and Psychiatry*, *61*(8), 899–913. https://doi.org/10.1111/jcpp.13294

Oh, J., Jang, S., Kim, H., & Kim, J. (2020). Efficacy of mobile app-based interactive cognitive behavioral therapy using a chatbot for panic disorder. *International Journal of Medical Informatics*, *140*, 104171. https://doi.org/10.1016/j.ijmedinf.2020.104171

Orosa-Duarte, Á., Mediavilla, R., Muñoz-Sanjose, A., Palao, Á., Garde, J., López-Herrero, V., Bravo-Ortiz, M., Bayón, C., & Rodríguez-Vega, B. (2021). Mindfulness-based mobile app reduces anxiety and increases self-compassion in healthcare students: A randomised controlled trial. *Medical Teacher*, 1–21. https://doi.org/10.1080/0142159x.2021.1887835

O'Toole, M. S., Arendt, M. B., & Pedersen, C. M. (2019). Testing an App-Assisted Treatment for Suicide Prevention in a randomized controlled Trial: Effects on suicide risk and Depression. *Behavior Therapy*, *50*(2), 421–429. https://doi.org/10.1016/j.beth.2018.07.007

Peake, E., Miller, I., Flannery, J., Chen, L., Lake, J., & Padmanabhan, A. (2024). Preliminary efficacy of a digital intervention for adolescent Depression: randomized controlled trial. *Journal of Medical Internet Research*, *26*. https://doi.org/10.2196/48467

Pham, Q., Khatib, Y., Stansfeld, S., Fox, S., & Green, T. (2016). Feasibility and Efficacy of an MHealth Game for Managing Anxiety: "Flowy" Randomized Controlled pilot trial and design evaluation. *Games for Health Journal*, *5*(1), 50–67. https://doi.org/10.1089/g4h.2015.0033

Ponzo, S., Morelli, D., Kawadler, J. M., Hemmings, N. R., Bird, G., & Plans, D. (2020). Efficacy of the Digital Therapeutic Mobile App BioBase to Reduce Stress and Improve Mental Well-Being Among University Students: Randomized Controlled Trial. *JMIR Mhealth And Uhealth*, *8*(4), e17767. https://doi.org/10.2196/17767

Possemato, K., Johnson, E., Barrie, K., Ghaus, S., Noronha, D., Wade, M., Greenbaum, M. A., Rosen, C., Cloitre, M., Owen, J., Jain, S., Beehler, G., Prins, A., Seal, K., & Kuhn, E. (2023). A randomized clinical trial of Clinician-Supported PTSD coach in VA primary care patients. *Journal of General Internal Medicine*, *38*(S3), 905–912. https://doi.org/10.1007/s11606-023-08130-6

Possemato, K., Kuhn, E., Johnson, E., Hoffman, J. E., Owen, J. E., Kanuri, N., De Stefano, L., & Brooks, E. (2016). Using PTSD Coach in primary care with and without clinician

support: a pilot randomized controlled trial. *General Hospital Psychiatry*, *38*, 94–98. https://doi.org/10.1016/j.genhosppsych.2015.09.005

Qin, X., Liu, C., Zhu, W., Chen, Y., & Wang, Y. (2022). Preventing postpartum depression in the early postpartum period using an APP-Based Cognitive Behavioral Therapy Program: a pilot randomized controlled study. *International Journal of Environmental Research and Public Health*, *19*(24), 16824. https://doi.org/10.3390/ijerph192416824

Raevuori, A., Vahlberg, T., Korhonen, T., Hilgert, O., Aittakumpu-Hyden, R., & Forman-Hoffman, V. (2021). A therapist-guided smartphone app for major depression in young adults: A randomized clinical trial. *Journal of Affective Disorders*, *286*, 228–238. https://doi.org/10.1016/j.jad.2021.02.007

Rajabi Majd, N., Broström, A., Ulander, M., Lin, C., Griffiths, M. D., Imani, V., Ahorsu, D. K., Ohayon, M. M., & Pakpour, A. H. (2020). Efficacy of a Theory-Based Cognitive Behavioral Technique App-Based Intervention for patients with insomnia: Randomized Controlled trial. *Journal of Medical Internet Research*, *22*(4), e15841. https://doi.org/10.2196/15841

Reid, S. C., Kauer, S. D., Hearps, S. J., Crooke, A. H., Khor, A. S., Sanci, L. A., & Patton, G. C. (2011). A mobile phone application for the assessment and management of youth mental health problems in primary care: a randomised controlled trial. *BMC Family Practice*, *12*(1). https://doi.org/10.1186/1471-2296-12-131

Riordan, K. M., Simonsson, O., Frye, C., Vack, N. J., Sachs, J., Fitch, D., Goldman, R. I., Chiang, E. S., Dahl, C. J., Davidson, R. J., & Goldberg, S. B. (2024). How often should I meditate? A randomized trial examining the role of meditation frequency when total amount of meditation is held constant. *Journal of Counseling Psychology, 71*, 104-114.

Rocamora González, C., Rodríguez Vega, B., Torrijos Zarcero, M., Mediavilla, R., Bouzó Molina, N., Plaza Fernández, R., Pascual Migueláñez, I., & Palao Tarrero, Á. (2022). Mindfulness based intervention through mobile app for colorectal cancer people awaiting surgery: a randomized clinical trial. *Cirugía Española (English Edition)*, *100*(12), 747–754. https://doi.org/10.1016/j.cireng.2022.08.008

Roepke, A. M., Jaffee, S. R., Riffle, O. M., McGonigal, J., Broome, R., & Maxwell, B. (2015). Randomized Controlled Trial of SuperBetter, a Smartphone-Based/Internet-Based Self-Help Tool to Reduce Depressive Symptoms. *Games For Health Journal*, *4*(3), 235–246. https://doi.org/10.1089/g4h.2014.0046

Röhr, S., Jung, F. U., Pabst, A., Grochtdreis, T., Dams, J., Nagl, M., Renner, A., Hoffmann, R., König, H., Kersting, A., & Riedel-Heller, S. G. (2021). A Self-Help App for Syrian Refugees With Posttraumatic Stress (Sanadak): Randomized Controlled Trial. *JMIR Mhealth And Uhealth*, *9*(1), e24807. https://doi.org/10.2196/24807

Roncero, M., Belloch, A., & Doron, G. (2019). Can Brief, Daily Training Using a Mobile App Help Change Maladaptive Beliefs? Crossover Randomized Controlled Trial. *JMIR Mhealth And Uhealth*, *7*(2), e11443. https://doi.org/10.2196/11443

Roy, A., Hoge, E. A., Abrante, P., Druker, S., Liu, T., & Brewer, J. A. (2021). Clinical Efficacy and Psychological Mechanisms of an App-Based Digital Therapeutic for Generalized Anxiety Disorder: Randomized Controlled Trial. *Journal Of Medical Internet Research*, *23*(12), e26987. https://doi.org/10.2196/26987

Sawyer, A., Kaim, A., Le, H., McDonald, D., Mittinty, M., Lynch, J., & Sawyer, M. (2019). The Effectiveness of an App-Based Nurse-Moderated Program for New Mothers With Depression and Parenting Problems (eMums Plus): Pragmatic Randomized Controlled Trial. *Journal Of Medical Internet Research*, *21*(6), e13689. https://doi.org/10.2196/13689

Schwob, J. T., & Newman, M. G. (2023). Brief imaginal exposure exercises for social anxiety disorder: A randomized controlled trial of a self-help momentary intervention app. *Journal Of Anxiety Disorders*, *98*, 102749. https://doi.org/10.1016/j.janxdis.2023.102749

Seo, J., Kim, S., Na, H., Kim, J., & Lee, H. (2022). Effectiveness of a Mobile Application for Postpartum Depression Self-Management: Evidence from a Randomised Controlled Trial in South Korea. *Healthcare*, *10*(11), 2185. https://doi.org/10.3390/healthcare10112185

Sharma, G., Schlosser, L., Jones, B. D. M., Blumberger, D. M., Gratzer, D., Husain, M. O., Mulsant, B. H., Rappaport, L., Stergiopoulos, V., & Husain, M. I. (2022). Brief App-Based Cognitive Behavioral Therapy for Anxiety Symptoms in Psychiatric Inpatients: Feasibility Randomized Controlled Trial. *JMIR Formative Research*, *6*(11), e38460. https://doi.org/10.2196/38460

Smith, E. N., Santoro, E., Moraveji, N., Susi, M., & Crum, A. J. (2020). Integrating wearables in stress management interventions: Promising evidence from a randomized trial. *International Journal of Stress Management*, *27*(2), 172–182. https://doi.org/10.1037/str0000137

Smith, J. L., Allen, J. W., Haack, C. I., Wehrmeyer, K. L., Alden, K. G., Lund, M. B., & Mascaro, J. S. (2021). Impact of App-Delivered Mindfulness Meditation on Functional Connectivity, Mental Health, and Sleep Disturbances Among Physician Assistant Students: Randomized, Wait-list Controlled Pilot Study. *JMIR Formative Research*, *5*(10), e24208. https://doi.org/10.2196/24208

Smith, R. B., Mahnert, N. D., Foote, J., Saunders, K. T., Mourad, J., & Huberty, J. (2021). Mindfulness effects in obstetric and gynecology patients during the Coronavirus Disease 2019 (COVID-19) pandemic. *Obstetrics and Gynecology*, *137*(6), 1032–1040. https://doi.org/10.1097/aog.0000000000004316

Soltani, Z., Parizad, N., Radfar, M., Alinejad, V., Arzanlo, M., & Haghighi, M. (2024). The effect of the Yara smartphone application on anxiety, sleep quality, and suicidal thoughts in patients with major depressive disorder in Iran: a randomized controlled trial. *BMC Psychiatry*, *24*(1). https://doi.org/10.1186/s12888-024-05688-1

Stiles-Shields, C., Montague, E., Kwasny, M. J., & Mohr, D. C. (2019). Behavioral and cognitive intervention strategies delivered via coached apps for depression: Pilot trial. *Psychological Services*, *16*(2), 233–238. https://doi.org/10.1037/ser0000261

Stolz, T., Schulz, A., Krieger, T., Vincent, A., Urech, A., Moser, C., Westermann, S., & Berger, T. (2018). A mobile app for social anxiety disorder: A three-arm randomized controlled trial comparing mobile and PC-based guided self-help interventions. *Journal of Consulting and Clinical Psychology*, *86*(6), 493–504. https://doi.org/10.1037/ccp0000301

Sun, S., Lin, D., Goldberg, S., Shen, Z., Chen, P., Qiao, S., Brewer, J., Loucks, E., & Operario, D. (2022). A mindfulness-based mobile health (mHealth) intervention among

psychologically distressed university students in quarantine during the COVID-19 pandemic: A randomized controlled trial. *Journal of Counseling Psychology*, *69*(2), 157–171. https://doi.org/10.1037/cou0000568

Sun, Y., Li, Y., Wang, J., Chen, Q., Bazzano, A. N., & Cao, F. (2021). Effectiveness of Smartphone-Based Mindfulness Training on Maternal Perinatal Depression: Randomized controlled trial. *Journal of Medical Internet Research*, *23*(1), e23410. https://doi.org/10.2196/23410

Taylor, H., Cavanagh, K., Field, A. P., & Strauss, C. (2022). Health Care Workers' Need for Headspace: Findings From a Multisite Definitive Randomized Controlled Trial of an Unguided Digital Mindfulness-Based Self-help App to Reduce Healthcare Worker Stress. *JMIR Mhealth And Uhealth*, *10*(8), e31744. https://doi.org/10.2196/31744

Taylor, R. W., Male, R., Economides, M., Bolton, H., & Cavanagh, K. (2023). Feasibility and preliminary efficacy of digital interventions for depressive symptoms in working adults: multiarm randomized controlled trial. *JMIR Formative Research*, *7*, e41590. https://doi.org/10.2196/41590

Thabrew, H., Boggiss, A. L., Lim, D., Schache, K., Morunga, E., Cao, N., Cavadino, A., & Serlachius, A. S. (2022). Well-being app to support young people during the COVID-19 pandemic: randomised controlled trial. *BMJ Open*, *12*(5), e058144. https://doi.org/10.1136/bmjopen-2021-058144

Tighe, J., Shand, F., Ridani, R., Mackinnon, A., De La Mata, N., & Christensen, H. (2017). Ibobbly mobile health intervention for suicide prevention in Australian Indigenous youth: a pilot randomised controlled trial. *BMJ Open*, *7*(1). https://doi.org/10.1136/bmjopen-2016-013518

Toh, S. H. Y., Tan, J. H. Y., Kosasih, F. R., & Sündermann, O. (2022). Efficacy of the mental health app Intellect to Reduce Stress: randomized controlled trial with a 1-Month follow-up. *JMIR Formative Research*, *6*(12), e40723. https://doi.org/10.2196/40723

Tønning, M. L., Faurholt-Jepsen, M., Frost, M., Martiny, K., Tuxen, N., Rosenberg, N., Busk, J., Winther, O., Melbye, S. A., Thaysen-Petersen, D., Aamund, K. A., Tolderlund, L., Bardram, J. E., & Kessing, L. V. (2021). The effect of smartphone-based monitoring and treatment on the rate and duration of psychiatric readmission in patients with unipolar depressive disorder: The RADMIS randomized controlled trial. *Journal Of Affective Disorders*, *282*, 354–363. https://doi.org/10.1016/j.jad.2020.12.141

Torok, M., Han, J., McGillivray, L., Wong, Q., Werner-Seidler, A., O'Dea, B., Calear, A., & Christensen, H. (2022). The effect of a therapeutic smartphone application on suicidal ideation in young adults: Findings from a randomized controlled trial in Australia. *PLoS Medicine*, *19*(5), e1003978. https://doi.org/10.1371/journal.pmed.1003978

Van Aubel, E., Bakker, J. M., Batink, T., Michielse, S., Goossens, L., Lange, I., Schruers, K., Lieverse, R., Marcelis, M., Van Amelsvoort, T., Van Os, J., Wichers, M., Vaessen, T., Reininghaus, U., & Myin-Germeys, I. (2020). Blended care in the treatment of subthreshold symptoms of depression and psychosis in emerging adults: A randomised controlled trial of Acceptance and Commitment Therapy in Daily-Life (ACT-DL). *Behaviour Research And Therapy*, *128*, 103592. https://doi.org/10.1016/j.brat.2020.103592

Van Stolk-Cooke, K., Wielgosz, J., Hallenbeck, H. W., Chang, A., Rosen, C., Owen, J., & Kuhn, E. (2023). The PTSD Family Coach App in Veteran Family Members: Pilot Randomized controlled trial. *JMIR Formative Research*, *7*, e42053. https://doi.org/10.2196/42053

Vázquez, F. L., Blanco, V., Hita, I., Torres, Á. J., Otero, P., Páramo, M., & Salmerón, M. (2023). Efficacy of a Cognitive Behavioral Intervention for the Prevention of Depression in Nonprofessional Caregivers Administered through a Smartphone App: A Randomized Controlled Trial. *Journal Of Clinical Medicine*, *12*(18), 5872. https://doi.org/10.3390/jcm12185872

Vereschagin, M., Wang, A. Y., Richardson, C. G., Xie, H., Munthali, R. J., Hudec, K. L., Leung, C., Wojcik, K. D., Munro, L., Halli, P., Kessler, R. C., & Vigo, D. V. (2024). Effectiveness of the minder mobile Mental Health and Substance use intervention for university students: randomized controlled trial. *Journal of Medical Internet Research*, *26*. https://doi.org/10.2196/54287

Versluis, A., Verkuil, B., Spinhoven, P., & Brosschot, J. F. (2018). Effectiveness of a smartphone-based worry-reduction training for stress reduction: A randomized-controlled trial. *Psychology and Health*, *33*(9), 1079–1099. https://doi.org/10.1080/08870446.2018.1456660

Vollert, B., Müller, L., Jacobi, C., Trockel, M., & Beintner, I. (2023). Effectiveness of an App-Based Short Intervention to Improve Sleep: Randomized Controlled Trial. *JMIR Mental Health*, *10*, e39052. https://doi.org/10.2196/39052

Wang, L., Guo, Y., Liu, Y., Yan, X., & Ding, R. (2022). The effects of a mobile phone-based psychological intervention program on stress, anxiety and self-efficacy among undergraduate nursing students during clinical practice: A randomized controlled trial. *Journal Of Professional Nursing*, *42*, 219–224. https://doi.org/10.1016/j.profnurs.2022.07.016

Watson-Singleton, N. N., & Pennefather, J. (2023). Using a Randomized Clinical Trial to Test the Efficacy of a Culturally Responsive Mobile Health Application in African Americans. *Behavior Therapy*, *55*(4), 813–824. https://doi.org/10.1016/j.beth.2023.12.002

Watts, S., Mackenzie, A., Thomas, C., Griskaitis, A., Mewton, L., Williams, A., & Andrews, G. (2013). CBT for depression: a pilot RCT comparing mobile phone vs. computer. *BMC Psychiatry*, *13*(1). https://doi.org/10.1186/1471-244x-13-49

Werner-Seidler, A., Li, S. H., Spanos, S., Johnston, L., O'Dea, B., Torok, M., Ritterband, L., Newby, J. M., Mackinnon, A. J., & Christensen, H. (2023). The effects of a sleep-focused smartphone application on insomnia and depressive symptoms: a randomised controlled trial and mediation analysis. *Journal Of Child Psychology And Psychiatry*, *64*(9), 1324–1335. https://doi.org/10.1111/jcpp.13795

Wilhelm, S., Weingarden, H., Greenberg, J. L., Hoeppner, S. S., Snorrason, I., Bernstein, E. E., McCoy, T. H., & Harrison, O. T. (2022). Efficacy of App-Based Cognitive Behavioral Therapy for Body Dysmorphic Disorder with Coach Support: Initial Randomized Controlled Clinical Trial. *Psychotherapy and Psychosomatics*, *91*(4), 277–285. https://doi.org/10.1159/000524628

Winslow, B. D., Kwasinski, R., Hullfish, J., Ruble, M., Lynch, A., Rogers, T., Nofziger, D., Brim, W., & Woodworth, C. (2022). Automated stress detection using mobile application

and wearable sensors improves symptoms of mental health disorders in military personnel. *Frontiers in Digital Health*, *4*. https://doi.org/10.3389/fdgth.2022.919626

Wong, V. W., Tong, J. T., Shi, N., Ng, C. H., Sarris, J., & Ho, F. Y. (2021). Smartphone-delivered multicomponent lifestyle medicine intervention for improving mental health in a nonclinical population: a randomized controlled trial. *Frontiers in Public Health*, *11*.https://doi.org/10.3389/fpubh.2023.1231981

Yang, Y. J., & Chung, K. (2023). Pilot Randomized Control Trial of an App-Based CBT Program for Reducing Anxiety in Individuals with ASD without Intellectual Disability. *Journal Of Autism And Developmental Disorders*, *53*(4), 1331–1346. https://doi.org/10.1007/s10803-022-05617-9

Yoon, S., Lee, S., Suh, H., Chung, S., & Kim, J. W. (2022). Effects of mobile mindfulness training on mental health of employees: A CONSORT-compliant pilot randomized controlled trial. *Medicine*, *101*(35), e30260. https://doi.org/10.1097/md.0000000000030260

Zainal, N. H., & Newman, M. G. (2023). A randomized controlled trial of a 14-day mindfulness ecological momentary intervention (MEMI) for generalized anxiety disorder. *European Psychiatry*, *66*(1). https://doi.org/10.1192/j.eurpsy.2023.2

Zainal, N. H., Tan, H. H., Hong, R. Y. S., & Newman, M. G. (2024). Testing the efficacy of a Brief, Self-Guided Mindfulness Ecological Momentary Intervention on Emotion Regulation and Self-Compassion in Social Anxiety Disorder: randomized Controlled trial. *JMIR Mental Health*, *11*. https://doi.org/10.2196/53712

Zhang, C., Liu, Y., Guo, X., Liu, Y., Shen, Y., & Ma, J. (2023). Digital Cognitive Behavioral Therapy for Insomnia Using a Smartphone Application in China. *JAMA Network Open*, *6*(3), e234866. https://doi.org/10.1001/jamanetworkopen.2023.4866

Zhang, X., Li, Y., Wang, J., Mao, F., Wu, L., Huang, Y., Sun, J., & Cao, F. (2023). Effectiveness of Digital Guided Self-help Mindfulness Training During Pregnancy on Maternal Psychological Distress and Infant Neuropsychological Development: Randomized Controlled Trial. *Journal Of Medical Internet Research*, *25*, e41298. https://doi.org/10.2196/41298

Zhao, C., Zhao, Z., Levin, M. E., Lai, L., Shi, C., Hu, J., Chen, W., & Ren, Z. (2023). Efficacy and acceptability of mobile application-delivered acceptance and commitment therapy for posttraumatic stress disorder in China: A randomized controlled trial. *Behaviour Research And Therapy*, *171*, 104440. https://doi.org/10.1016/j.brat.2023.104440

Zhou, X., Edirippulige, S., Jones, A., Bai, X., Smith, A. C., & Bambling, M. (2023). The feasibility, acceptability and efficacy of an app-based intervention (the Coping Camp) in reducing stress among Chinese school adolescents: A cluster randomised controlled trial. *PLoS ONE*, *18*(11), e0294119. https://doi.org/10.1371/journal.pone.0294119

Table S3. Sample and study characteristics of included studies (N=206)

					Mental health app						Outcome measure	
First Author (year)	Country	Population	M age (SD)	% female	Name	Technique	Primary Target	Intervention Group (n)	Comparison Group (n)	FU (weeks)	Depression	Anxiety
Abbasalizadeh (2024)	Iran	ICU nurses with at least 6 months experience working in an ICU	29.58 (4.68)	28.3	Resilience	Psychoeducation	General mental health	mHealth (30)	Control Group (30)	20	-	DASS-21
Abbott (2023)	United States	Adults with elevated anxiety symptoms	24 (9)	80.4	Headspace	Mindfulness	Anxiety and worry	Headspace (97)	Waitlist (66)	4, 8	-	BAI
Aboody (2020)	Israel	Female college students who speak Hebrew and have an active social media account	23.51 (1.45)	100	GGBI	CBT	General mental health	GGBI (48)	Waitlist (42)	2, 4	DASS-D	-
Abramovitch (2024)	United States	Undergraduate adult students at a large public university in the southeastern United States	18.78 (0.97)	85.70	GG-OCD app	CBT	Perfectionism	GG-OCD app (35)	Waitlist (35)	2, 4	DASS-D	DASS-A
Ahorsu (2020)	Iran	Individuals diagnosed with epilepsy and having moderate to severe insomnia	38.18	58.4	CBT-I APP	CBT	Insomnia	CBT-I APP (160)	Information resources (160)	4, 12, 24	HADS-D	HADS-A
Akechi (2023)	United States	Breast cancer patients between 20-49 years	43.95 (4.53)	100.0	PST and BA app	CBT	Concern about recurrence disease	PST and BA app (223)	Usual care (224)	8, 24	HADS-D	HADS-A
Al-Refae (2021)	Canada	Residents of Canada aged 18 and above	25.24 (8.74)	79	Serene app	Multidisciplinary	General mental health	Serene app (127)	Waitlist (118)	4	DASS-D	DASS-A
Anastasiadou (2020)	Spain	Patients older than 12 years, diagnosed with an eating disorder	18.06 (6.04)	91.4	TCApp	CBT	General mental health	F2F therapy and TCApp (53)	F2F therapy (53)	12	BDI-2	STAI
Araya (2021)	United Kingdom/Peru	Adults with depressive symptoms who were being treated for hypertension and/or diabetes	56	86.5	Digital intervention (CONEMO)	Behavioral activation	Depression	Digital intervention (CONEMO) (440)	(Enhanced) usual care (440)	12, 24	PHQ9	-
Araya (2021)	United Kingdom/Peru	Adults with depressive symptoms who were being treated for hypertension and/or diabetes	59.7	81.5	Digital intervention (CONEMO)	Behavioral activation	Depression	Digital intervention (CONEMO) (217)	(Enhanced) usual care (215)	12, 24	PHQ9	-
Bakker (2018)	Australia	NR	34.20 (12.10)	80	MoodKit, MoodPrism, MoodMission	CBT	General mental health	MoodKit (56), MoodPrism (56), MoodMission (50)	Waitlist (64)	4	PHQ9	GAD7
Barroso (2020)	United States	Adults with HIV and chronic fatigue	51.2 (9.9)	63.3	CBSM	CBT	Fatigue	CBSM (15)	Placebo App (15)	5, 10, 22	BDI-2	STAI-S STAI-T
Bear (2022)	New Zealand	Mothers of children betweem 0-12 months	31.16 (4.8	100.0	Smiling Mind	Mindfulness	Postnatal distress	Smiling Mind (49)	Placebo app (50)	8, 12	DASS-D	DASS-A
Bell (2023)	Australia	Young people from the general population with clinical levels of depression and anxiety	20.60 (2.7)	62	Mello app	Multidisciplinary	General mental health	Mello app (29)	No intervention (26)	3, 6	PHQ8	GAD7
Ben-Zeev (2018)	United States	Adults with schizophrenia, schizoaffective, bipolar or major depressive	49 (9.95)	39	FOCUS	Multidisciplinary	General mental health	FOCUS (82)	Group treatment WRAP (81)	12, 24	BDI-2	-

Ben-Zeev (2021)	United States	Adults with bipolar disorder, MDD, schizophrenia or schizoaffective disorder	37.92 (11.61)	83.8	CORE App	Nonspecific	General mental health	CORE App (154)	Waitlist and delayed CORE App (161)	4, 8	BDI-2	GAD7
Birney (2016)	United States	Working adults with mild-to-moderate depression	40.65 (11.39)	76.7	MoodHacker	Multidisciplinary	Depression	MoodHacker (150)	Bibliotherapy (150)	6, 10	PHQ9	-
Birrell (2023)	Australia	Year 9 students visiting Australian secondary schools	15.2 (0.4)	43.4	Mind your Mate Intervention	Nonspecific	General mental health	Mind your Mate Intervention (88)	Education program (78)	24, 48	PHQ9	GAD7
Boettcher (2018)	Sweden	Patients diagnosed with SAD	35.40 (12.25)	77.0	Challenger App	CBT Self-Help and Exposure	Social Anxiety	Bibliotherapy + Challenger App (70) and Bibilotherpy + delayed App (70)	Waitlist (69)	3, 7, 10, 14, 16, 52	PHQ9	GAD7
Borjalilu (2019)	Iran	University students with elevated levels of stress	24.29 (3.21)	71.0	Aramgar	Mindfulness	Stress	Aramgar (20); Face-to-face therapy + Aramgar (28)	Usual care (20)	6	DASS-D	DASS-A
Bostock (2016)	United Kingdom	Employees from a Fortune 500 company	33.6 (6.01)	33.3	Sleepio	CBT	Insomnia	Sleepio (135)	Waitlist (135)	8, 22	PHQ2	GAD2
Bostock (2019)	United Kingdom	Employees at two UK companies	35.50 (7.70)	60	Headspace	Mindfulness	General mental health	Headspace (128)	Waitlist (110)	8	HADS-D	HADS-A
Bruehlman-Senecal(2020)	United States	Incoming first-year students (aged 18-25 years) at a large public university in the US not living with their parents	18.68 (0.35)	59.3	NodApp	Multidisciplinary	Loneliness	NodApp (100)	Waitlist (121)	4, 8	PHQ9	GAD7
Bruhns (2021)	Germany	Adult students at a German university	22.98 (3.36)	89	MCT & More	Multidisciplinary	General mental health	MCT & More (208)	Waitlist (215)	4	PHQ9	-
Bruhns (2023)	Germany	Individuals with a diagnosis of depression (ICD-10 and DSM-5)	39.04 (12.90)	55.4	MCT & More	Multidisciplinary	General mental health	MCT & More + info video (47) and MCT & More + no info video (32)	Waitlist (80)	4	PHQ9	-
Bröcker (2024)	South Africa	Trauma-exposed adults with PTSD, scoring ≥23 on the CAPS-5	37.40 (11.35)	88.7	PTSD Couch app (counsellor-supported)	Multidisciplinary	General mental health	PTSD-Couch – CS (32)	Usual care (30)	4, 8, 12	DASS-21	DASS-21
Børøsund (2020)	Norway	Cancer patients	52 (11.2)	72.0	StressProffen	CBT	Stress	Stressproffen (87)	Usual care (88)	12	HADS-D	HADS-A
Børøsund (2022)	Norway	Adult Cancer survivors 1 year or less after their treatment	52 (11.3)	82	Stressproffen	Multidisciplinary	Stress	Stressproffen (87)	Usual Care (88)	24, 48	HADS-D	HADS-A
Cardi (2022)	Italy	Adults with episodes of binge eating	-	95.7	Food ICT Group	Nonspecific	Eating disorder	Food ICT Group (44)	Waitlist (50)	2, 3	DASS-D	DASS-A
Carl (2020)	United Kingdom	Individuals diagnosed with GAD, aged ≥18	30.90 (10.70)	68.4	Daylight	CBT	General mental health	Daylight (128)	Waitlist group (128)	3, 6, 10	PHQ9	GAD7
Carli (2022)	Italy and Portugal	Cancer patients	59.40 (10.68)	44.2	NEVERMIND	Multidisciplinary	Depression	NEVERMIND (213)	Usual care (212)	12, 24	BDI-2	-
Catuara-Solarz (2022)	Spain	Adults working in the United Kingdom	40 (6.10)	54	Foundations app	Multidisciplinary	General mental health	Foundations app (95)	Waitlist (95)	2, 4	-	GAD7

Cerea (2020)	Italy	University students scoring above the clinical threshold on ROCD symptoms, aged 20-24 years	22.00 (1.32)	76.0	GGRO	Multidisciplinary	General mental health	immediate-use App/iApp group (25)	delayed-use App/dApp group (25)	2, 4	DASS-21	DASS-21, SIAS
Cerea (2022)	Italy	Italian women at high-risk of developing Body Image Disorders (BIDs), aged 20–30 years	22.82 (2.11)	100.0	GGBI	CBT	General mental health	immediate-use App/iApp group (47)	delayed-use App/dApp group (48)	2, 4	-	SIAS
Chan (2021)	China	Individuals with comorbid depression and insomnia	27.30 (7.20)	73.0	CBT-I Intervention	CBT	General mental health	CBT-I Intervention (167)	Waitlist Group (153)	6, 12	CESD	HADS-A
Comtois (2022)	United States	People who were unemployed because of COVID-19	31.1 (9.5)	56.1	COVID Coach<br>Calm<br>7 Cups of Tea	Nonspecific	General mental health	COVID Coach (212)<br>Calm (204)<br>7 Cups of Tea (209)	Mood monitoring (Beautiful mood) (213)	4	PHQ9	GAD7
Cox (2019)	United States	Intensive Care Unit patients	49.50 (15.10)	44.0	mMT, tMT	Mindfulness	General mental health	mMT<br>(31), tMT (31)	Education program (18)	4, 12	PHQ9	GAD7
Cox (2023)	United States	Patients with a cardiopulmonary diagnosis	49.30 (13.20)	60.0	Blueprint with therapist, Blueprint without therapist	CBT	General mental health	Blueprint with therapist (16), Blueprint without therapist (14)	Usual Care Control (15)	4, 12	HADS-D	HADS-A
Dahne (2019)	United States	Latinx adults with limited English proficiency	36.05 (sd)	66.7	Aptivate	CBT	Depression	Aptivate (22)	iCouch (9)<br><br>Usual Care (11)	1, 2, 3, 4, 5, 6, 7, 8	BDI-2	-
Dahne (2019)	United States	Adults with depressive symptoms	43.79 (sd)	84.6	Moodivate	CBT	Depression	Moodivate (24)	MoodKit (19)<br><br>Usual Care (9)	1, 2, 3, 4, 5, 6, 7, 8	BDI-2	-
Danieli (2022)	Italy	Active workers aged over 55 years experiencing stress symptoms and mild-to-moderate anxiety	55.58 (5.08)	78	SMT-CBT, SMT-CBT + PHA,<br>PHA only	CBT,<br>CBT,<br>Chatbot	General mental health	SMT-CBT (16),<br>SMT-CBT + PHA (16),<br>PHA only (14)	Waitlist (14)	4, 8, 20	SCL-D, PHQ8	SCL-A, GAD7
De Kock (2022)	United Kingdom	Health and social care staff working in NHS	-	88.2	My Possible Self (MPS)	Multidisciplinary	Psychological well-being	NHS Highland Staff Wellbeing Project (NHSWBP) (51)<br><br>My Possible Self (MPS) (60)	Waitlist (58)	2, 4	PHQ9	GAD7
Deady (2022)	Australia	Working Australians in male-dominated industries	40.26 (10.63)	25.8	HeadGear	Multidisciplinary	Depression	HeadGear (1128)	Mood monitoring (1143)	5, 12, 52	PHQ9	-
Deady (2023)	Australia	Adult australian residents who are employed and feel stressed	42.96 (10.07)	71	Anchored	Multidisciplinary	Depression	Anchored (1056)	Psychoeducation (1056)	4, 12, 24	PHQ9	GAD7
Depp (2015)	United States	Outpatients diagnosed with either Bipolar Disorder 1 or 2	47.50 (12.80)	58.5	PRISM	Psychoeducation	Mood symptoms	PRISM (41)	Mood monitoring (41)	6, 12, 24	MADRS	-
DiNardo (2022)	United States	Veterans with type 1 or 2 diabetes	60.7 (10.6)	8.3	Mind-STRIDE	Mindfulness	General mental health	Mind-STRIDE and diabetes self-management education and support (65)	Diabetes self-management education and support (67)	12, 24	PHQ8	-
Dingwall (2021)	Australia	Indigenous Australians undergoing hemodialysis	55 (9.4)	71.8	ALMhi Stay Strong App	Nonspecific	General mental health	ALMhi Stay Strong App (62)	Hep B Story App and delayed ALMhi Stay Strong App (61)	12, 24	PHQ9	-

									Usual care and delayed ALMhi Stay Strong App (33)			
Ditton (2023)	Australia	Medical students	24 (5.48)	61.5	ACT app	ACT	General mental health	ACT app (individualised) (37)	ACT app (nonindividualised) (36)<br><br>Waitlist (35)	5	DASS-D	DASS-A
Domar (2023)	United States	Men who were each part of a couple experiencing infertility	33.70 (4.5)	0	FertiStrong	Multidisciplinary	General mental health	FertiStrong (20)	No intervention (19)	4	HADS-D	HADS-A
Donker (2019)	Netherlands	Dutch adults from the general population with acrophobia symptoms	41.33	66.8	OPhobia VR	CBT	Acrophobia	OPhobia (96)	Waitlist (97)	3, 12	PHQ9	BAI
Donker (2022)	Netherlands	People from Dutch general population with aviophobia symptoms	42 (12.15)	83	VR-CBT-App	CBT	General mental health	VR-CBT-App (77)	Waitlist (77)	6 (12, 24)	PHQ9	BAI
Economides (2022)	United Kingdom	Working adults in the United Kingdom with an active account on Prolific	44.60 (14.3)	52	Unmind	Multidiscplinary	General mental health	Unmind CBT and ACT-based intervention for stress (94), anxiety (97), or worry (98)	Waitlist (94)	2, 6	PHQ8	GAD7
El-Jawahri (2023)	United States	Patients with acute myeloid leukemia (AML)	60.12	36.7	DreamLand	Nonspecific	General mental health	DreamLand (29)	Usual care (31)	3,6	HADS-D, PHQ9	HADS-A
Everitt (2021)	Australia	General population, aged 18-69	32.97 (10.92)	85.5	MoodTracker, ImproveYourMood, ImproveYourMood+	Mood monitoring, Mindfulness	General mental health	MoodTracker (58), ImproveYourMood (62), ImproveYourMood+ (60)	Waitlist group (55)	3, 7	PHQ9	GAD7
Faurholt-Jepsen (2021)	Denmark	Patients with bipolar disorder discharged from hospitalization	42.69 (13.48)	49.9	Monseo System	CBT	General mental health	Monseo System (47)	Usual care (51)	12, 24	HDRS-17, BDI, HDRS-6	-
Fiol-De Roque (2021)	Spain	Adult health care workers who provided health care to patients with COVID-19	41.37 (10.4)	83.2	PsyCovid App	Multidisciplinary	General mental health	PsyCovid App (248)	Information resources (234)	2	DASS-D	DASS-A
Fish (2019)	United States	University students	21.00	96.0	Headspace Mindfulness	Mindfulness	General mental health	Headspace Mindfulness (47)	Usual care (44)	2	PHQ9	-
Fitzpatrick (2017)	United States	Students who self-identify as having symptoms of anxiety and depression	22.20 (2.33)	67.0	Woebot	CBT	General mental health	Woebot (34)	Information resources (36)	2	PHQ9	GAD7
Flett (2019)	New Zealand	Undergraduate university students	20.08 (2.8)	70	Headspace, Smiling Mind	Mindfulness	General mental health	Headspace (72), Smiling Mind (63)	Placebo app (75)	1.5, 4	CESD	HADS-A
Forman Hoffman (2024)	United States	Adults living in Colorado	37 (12.79)	95	Meru Health Program	Multidisciplinary	Depression	Meru Health Program (54)	Waitlist (46)	6, 12	PHQ9	GAD7
Fuller-Tyszkiewicz (2020)	Australia	Australians (adult) supporting a friend or relative with a physical or mental condition	39.64 (6.13)	95	StressLess	Multidisciplinary y	General mental health	StressLess (73)	Active control: self-monitoring (110)	5, 12	DASS-D	DASS-A
Gao (2022)	United States	Individuals with elevated worry (PSWQ > 40) and insomnia symptoms	41.41	80.3	MT + TAU	Mindfulness	General mental health	MT + TAU (40)	Usual care (40)	8, 16	-	GAD7
Gao (2024)	China	University student athtletes	19.4 (1.5)	62.8	WeChat	Mindfulness	Anxiety	WeChat (150)	Education program (138)	6	-	Dispositional Anxiety

Ghaemi (2022)	United States	Participants with moderate acute psychotic exacerbation in schizophrenia	44.7 (11.29)	34.5	PEAR-004	Nonspecific	General mental health	PEAR-004 (56)	Placebo app (56)	4, 8, 12, 16	BDI-2	-
Ghanbari (2021)	Iran	Women with breast cancer	46.45 (9.29)	100	BCSzone	Nonspecific	General mental health	BCSzone (41)	Waitlist (41)	5	-	STAI
Gnanapragasam (2023)	England	NHS-affiliated members of staff	44.3	84.3	Foundations App	Multidisciplinary	Psychiatric morbidity	Foundations App (502)	Usual care (500)	4, 8	PHQ9	GAD7
Goldberg (2020)	United States	Adults with little meditation retreat experience	41.74 (12.52)	84.5	HMP	Multidisciplinary	General mental health	Awareness & Connection from HMP (121);<br>Awareness & Insight from HMP (107)	Waitlist (115)	4, 8	PROMIS-D	PROMIS-A
Graham (2020)	United States	Adults with elevated depression or anxiety levels	42.3 (13.8)	82	IntelliCare	Nonspecific	General mental health	IntelliCare (74)	Usual care (72)	4, 8, 12, 16	PHQ9	GAD7
Greer (2019)	United States	Incurable cancer patients with clinically significant anxiety symptoms	56.45 (11.30)	73.8	CBT mobile app	CBT	Anxiety	CBTmobile app (72)	Education program (73)	12	HADS-D, PHQ9	HAM-A, HADS-A
Grubbs (2022)	United States	Veterans with anxiety or depression diagnosis	46	74.6	Moving Forward	Problem solving therapy (PST)	General mental health	PST+ Moving Forward (33)	PST + Workbook (33)	6, 12	DASS-D	DASS-A
Guo (2020)	China	Adults in China with HIV from an outpatient clinic	28.30 (5.8)	7.7	Run4Love	Multidisciplinary	Depression	Run4Love (150)	Usual care (150)	12, 24, 39	CESD, PHQ9	-
Ha (2020)	South Korea	Individuals with elevated worry (PSWQ > 40) and insomnia symptoms	21.44	70.2	Spring	CBT	General mental health	Spring (34)	Waitlist group (34)	2	BDI-2	STAI-T
Ham (2019)	South Korea	Cancer patients who scored ≥ 16 on the BDI-2 and/or ≥ 39 on the STAI	44.17	85.7	HARU Today	CBT	General mental health	HARU Today (21), Attention Group (21)	Waitlist Group(21)	10	BDI-2	STAI-S, STAI-T
Hanssen (2020)	Netherlands	Individuals with a schizophrenia spectrum disorders (SZ) diagnosis	39.00	36.0	Smart App: Personalized Feedback	Nonspecific	General mental health	Smart App: Personalized Feedback (27)	Smart App: No Feedback (23)	3	CAPE	-
He (2022)	China	College students with depressive symptoms	18.78 (0.88)	37.2	Chatbot XiaoE	CBT	Depression	Chatbot XiaoE (49)	Bibliotherapy (49)<br><br>Placebo App (50)	1, 4	PHQ-9	-
Heim (2021)	Switzerland	Lebanese and displaced people living in Lebanon affected by adversity	27.3 (7.9)	67.4	Step-By-Step Intervention	CBT	General mental health	Step-By-Step Intervention (67)	Information resources (71)	8, 20	PHQ9	GAD7
Hensler (2022)	Sweden	Adults with exposure to traumatic event and mild to severe posttraumatic stress symptoms	42.78 (10.90)	91.6	PTSD Coach	Multidisciplinary	General mental health	PTSD Coach (89)	Waitlist Group (90)	12	PHQ9	-
Hilt (2023)	United States	Adolescents in the United States reporting moderate-to-high levels of rumination	13.78 (0.89)	59	Mindfulness + Mood monitoring	Mindfulness	Rumination	Mindfulness + Modd monitoring (72)	Placebo app: Mood monitoring (80)	3, 6, 12, 26	CDI	MASC
Hirshberg (2022)	United Stated	School system employees	42.58 (10.67)	88	Healthy Minds Program (HMP)	Mindfulness	General mental health	HMP (346)	Waitlist (320)	1, 2, 3, 4, 16	PROMIS-D	PROMIS-A
Horsch (2017)	Netherlands	Adults with relatively mild insomnia disorder	39.66 (13.44)	62.3	SleepCare	CBT	Insomnia	SleepCare (74)	Waitlist Group (77)	6, 12	CESD	HADS-A

Howells (2016)	United Kingdom	Adult authentic happiness seekers	40.70 (10.6)	88	Headspace Mindfulness	Mindfulness	General mental health	Headspace Mindfulness (97)	Catch Notes: Placebo app (97)	2	CESD	-
Huberty (2021)	United States	Adults with elevated insomnia symptoms	44.5 (14.6)	74.6	Calm App	Mindfulness	Insomnia	Calm App (113)	No intervention (127)	4, 8	HADS-D	HADS-A
Huberty (2022)	United States	Employees of a large consumer electronics retailer	-	50.3	Calm App	Mindfulness	General mental health	Calm App (585)	Waitlist (444)	2, 4, 6, 8	DASS-D	DASS-A
Hunt (2021)	United States	Adults with IBS	32 (10.2)	75.2	Zemedy	CBT	IBS	Zemedy (62)	Waitlist (59)	8	DASS-D, PHQ9	DASS-A
Hur (2018)	South Korea	Individuals diagnosed with Other Specified Depressive Disorder	23.71 (3.26)	88.2	Todac App	CBT	General mental health	Todac App (17)	Mood monitoring (17)	3	BDI-2	STAI-X2
Hwang (2019)	Korea	Nurses employed at college hospitals in Seoul	/	95	Test group	Nonspecific	Stress	Test group (30)	No intervention (30)	4	PHQ9	GAD7
Hwang (2022)	Korea	Individuals experiencing work-related stress	37.94 (9.31)	80.2	BetterLife program	Multidisciplinary	Perceived stress	BetterLife program (63)	Waitlist (63)	10	BDI-2	BAI
Imamura (2021)	Vietnam	Full time nurses in a large general hospital	33.10 (6.78)	85	Program	CBT	Not specified	Free-choice stress management (317); Fixed-order iCBT (316)	Treatment as usual (316)	12, 28	DASS21-D	DASS-21 A
Jannati (2020)	Iran	Mothers with Postpartum depression (PPD) aged ≥18	27.52	100.0	Happy Mom	CBT	Postpartum depression	Happy Mom (38)	No intervention (37)	8	EPDS	-
Järvelä-Reijonen (2020)	Finland	Working-age adults with psychological distress and overweight/obesity	49.57 (7.24)	83.8	Oiva	ACT	General mental health	Oiva (85); Face-to-face ACT (84)	No intervention (85)	10, 36	BDI-2	-
Kauer (2012)	Australia	Young adults between 14-24 years with emotional mental health issues	18.04 (3.2)	72.9	Mobietype	Nonspecific	Depression	Mobietype (69)	Placebo app (49)	3, 6	DASS-D	-
Keng (2022)	Singapore	Health care workers in Singapore during the COVID-19 pandemic	30.18 (6.19)	90	Headspace	Mindfulness	General mental health	Headspace (40)	Placebo app: Lumosity (40)	3, 7	DASS-D	DASS-A
Kenny (2020)	Ireland	Students aged 15-18 years	16.05 (0.76)	62	CopeSmart	Multidisciplinary	General mental health	CopeSmart (385)	No intervention (175)	4, 8	DASS-D	DASS-A
Kerber (2023)	Germany	Adults with clinically relevant symptoms of internalising disorders	38.3 (11.19)	73.6	MindDoc	Multidisciplinary	General mental health	MindDoc (523)	Usual care (522)	8, 24	PHQ9	GAD7
Kim (2024)	Korea	Individuals with a history of panic attacks	34.95 (12)	73.9	Digital app for panic disorder	Multidisciplinary	Panic disorder	Digital app for panic disorder (25)	Information resources (25)	4	-	HAM-A, GAD7
Kirykowicz (2023)	South Africa	Adults working in a government healthcare facility	33.4 (3.8)	58.8	COVID Coach	Nonspecific	General mental health	COVID Coach (16)	No intervention (18)	4	CES-D	STAI-S
Kloos (2022)	Netherlands	Dutch and Flemish adults with low well-being	52.90 (15)	80	ZENN-Gratitude app	Gratitude	General mental health	ZENN- Gratitude app (424)	Waitlist (425)	6, 12	PHQ9	GAD7
Kollei (2017)	Germany	Students with elevated body image problems	21.64	92.5	MT-BD app	Multidisciplinary	General mental health	MT-BD app (26)	Waitlist group (27)	2, 6	ADS	-
Kosasih (2023)	Singapore	Adults aged ≥18	22.05 (4.06)	74.6	Intellect App "Anxiety and Worry"	Multidisciplinary	General mental health	Intellect App "Anxiety and Worry" (160)	Intellect "Procrastination" (163)	2, 4	PHQ9	GAD7

Krafft (2019)	United Sates	Adults interested in phone-based self-help	21.79	70.4	Simple matrix app and Complex matrix app	ACT	General mental health	Simple matrix app (33); Complex matrix app (34)	No intervention (31)	2, 4	DASS-D	DASS-A
Kubo (2019)	United States	Cancer patients receiving chemotherapy	58.3 (14.34)	68	Headspace	Mindfulness	General mental health	Headspace (54)	Usual care (43)	8	HADS-D	HADS-A
		Informal caregivers of cancer patients	57.6 (17.65)	58	Headspace	Mindfulness	General mental health	Headspace (17)	Usual care (14)	8	HADS-D	HADS-A
Kubo (2024)	United States	Patients with metastatic solid malignancies or hematological cancers	66.44 (9.59)	69.9	Headspace	Mindfulness	General mental health	Headspace (52)	Usual care (51)	6, 12	HADS-D	HADS-A
Kuhn (2017)	United States	Adults having been exposed to a traumatic event more than 1 month ago who do not receive treatment	39	69	PTSD Coach App	Multidisciplinary	PTSD symptoms	PTSD Coach App (62)	Waitlist (58)	12, 24	PHQ8	-
Kuhn (2022)	United States	U.S. military veterans (aged 18-55 years) with a subclinical level of insomnia	44.48 (7.9)	42	Coach	CBT	Insomnia	Coach (25)	Waitlist (25)	6, 12	PHQ8	GAD7
Kulikov (2023)	United States	Adolescents with self-reported symptoms of depression, aged 13-21	17.51	78.0	Spark	CBT	General mental health	Spark (35)	Active Control Group (25)	5	PHQ8, MFQ-Ps	GAD7
Kusumadewi (2023)	Indonesia	Students with elevated anxiety symptoms	20.45 (0.71)	78.8	GAMA-AIMS	CBT	Anxiety	GAMA-AIMS (43)	Usual care (43)	1,2,3,4, 5,6,7	-	TMAS
Lacey (2023)	New Zealand	Adults with specific phobia	42.2 (13.2)	80.0	oVRcome	CBT	Specific phobia	oVRcome (63)	Waitlist (63)	6, 12	PHQ9	-
LaFreniere (2023)	United States	Students aged 18 to 24 with symptoms of GAD	18.66 (1.14)	90.6	SkilJoy Ecological Momentary Intervention	Positive Psychology	Generalized anxiety disorder	SkilJoy Ecological Momentary Intervention (41)	Placebo App (45)	1, 4	BDI-2	-
Lahtinen (2023)	Finland	University faculty, staff, and students	34.68 (10.77)	83.2	Mindfulness Intervention	Mindfulness	General mental health	Mindfulness Intervention (282)	Psychoeducation (279)	4, 12	BDI	GAD7
Laird (2022)	United States	Middle-aged adults (40-65 years) with elevated stress levels	51.45 (6.8)	65.5	Calm App	Mindfulness	Perceived stress	Calm App (39)	Placebo App (POD) (35)	4	HADS-D	HADS-A
Lee (2018)	Canada	Undergraduate students	20.62	63.2	DeStressify	Mindfulness	General mental health	DeStressify (102)	No intervention (104)	4	QIDS-SR	STAI-S, STAI-T
Lee (2023)	Korea	Office workers with elevated levels of perceived stress	35.2 (8.49)	78	Mobile app-based stress managemen t intervention mSMI	Multidisciplinary	Perceived stress	mSMI (39)	No intervention (43)	6	HADS-D	HADS-A
Levin (2018)	United States	Adults high in self-criticism	22.76 (7.02)	68.9	Cog. Defusion, Cof. Restructurin g	Multidisciplinary	General mental health	Cog. Defusion (30), Cof. Restructuring (29)	Waitlist group (28)	2	DASS-21	DASS-21
Levin_a (2019)	United States	University students 18+	21.9 (5.47)	68.1	ACT app	ACT	General mental health	Tailored ACT app (23); Random app (22)	No intervention (24)	4	DASS-D	DASS-A

Levin_b (2019)	United States	University students on the waitlist for the Counseling and Psychological Services center (CAPS), aged ≥18	20.43 (2,46)	100.0	SBT App	Mindfulness	General mental health	SBT App (10)	No intervention (13)	2, 4	CCAPS-Depression	CCAPS-General Anx, CCAPS-Social Anx
Lewis (2020)	United Kingdom	Individuals with severe mental illness	34.5	33.3	ClinTouch	Symptom monitoring	Psychotic symptoms	ClinTouch (40)	Usual care (41)	6. 12	CDS	-
Li (2019)	China	HIV or AIDS patients	27.5	7.7	Run4Love	CBT	Depression	Run4Love (15)	Education program (150)	12, 24	CES-D	-
Li (2022)	Hong Kong	Individuals with distress related to pain	41.35 (14.56)	83.8	WhatsApp-based MBi	Mindfulness	Pain	WhatsApp-based MBI (118)	Waitlist (117)	3, 7, 15	PHQ9	GAD7
Li (2022)	Hong Kong	Individuals with distress related to dysregulated eating	36.03 (11.59)	89.7	WhatsApp-based MBI	Mindfulness	Dysregulated eating	WhatsApp-based MBI (177)	Waitlist (174)	3, 7, 15	PHQ9	GAD7
Li (2022)	Hong Kong	Individuals with distress related to insomnia	42.13 (13.33)	79.6	WhatsApp-based MBI	Mindfulness	Insomnia	WhatsApp-based MBI (167)	Waitlist (166)	3, 7, 15	PHQ9	GAD7
Li (2024)	United States	Chinese women residing in the United States who experienced intimate partner violence (IPV)	36.16 (9.89)	100	SHE Intervention	Multidisciplinary	General mental health	SHE Intervention (25)	Information resources (26)	7, 15	PHQ9	GAD7
Lim (2023)	Malaysia	Medical practitioners (faculty staff, students, or corporate staff) aged >18	NA	64.2	ThoughtFull Chat App	Nonspecific	Reducing the symptoms of self-reported Depression, Anxiety, and Stress	ThoughtFullChat App (167)	No intervention (167)	12	DASS-21	DASS-21
Linardon (2022)	Australia	Individuals who self-reported the presence of binge eating	28.95 (8.17)	93.0	Break Binge Eating	CBT	General mental health	Break Binge Eating (197)	Waitlist group(195)	4, 8	PHQ4	PHQ4
Linardon (2023)	Australia	Adults who are binge eating	33.85 (9.83)	93.8	Break Binge Eating and Break the Diet Cycle	`CBT	Binge eating	Break Binge Eating (199); Break the Diet Cycle (199)	Waitlist (202)	4, 8	PHQ4	PHQ4
Litvin (2020)	Germany	Bosch UK employees	/	37.7	eQuoo	Multidisciplinary	General mental health	eQuoo (222)	Control group: Multidisciplinary (269), Waitlist (218)	2, 5	-	One-item Anxiety Scale
Litvin (2023)	United Kingdom	Adult college/university students	-	76.5	eQuoo	Multidisciplinary; CBT	General mental health	eQuoo (389); Sanvello Mental Health App (384)	No intervention (392)	5	PHQ8	GAD7
Liu (2022)	Taiwan	Women aged 25 to 40 in the postpartum period	31.81 (5.36)	100	We'll App	Nonspecific	Postpartumdepr essive symptoms	We'll App (65)	Waitlist (65)	8	EPDS	-
Liu (2022)	China	University students above the age 18 with elevated depressive symptoms	23.08 (1.76)	55.4	XiaoNan	CBT	General mental health	XiaoNan (41)	Bibliotherapy (42)	16	PHQ9	GAD7
Liu (2023)	China	Individuals with spinal cord injury	41.71 (12.14)	17.4	Together	Multidisciplinary	General mental health	Together (49)	Usual care (49)	12, 24	BDI-2	-
LooGee (2021)	Australia	Adults with elevated social anxiety symptoms	28.7 (12.4)	81	EMI App	CBT	Social anxiety	EMI App (28)	No intervention (27)	4	PHQ2	GAD2

Lu (2023)	China	Nurses with anxiety or depression symptoms	35.36 (7.22)	97.2	Rain Classroom	ACT	General mental health	Rain Classroom (72)	Waiting control group (73)	2, 3, 4, 5, 17	PHQ9	GAD7
Luangapichart (2022)	Thailand	Medical personnel suffering from burnout and stress	33.4 (7.92)	84.4	Mindful Senses Program	Mindfulness	General mental health	Mindful Senses Program and psychological self-help articles (45)	Psychological self-help articles (week 1-4) and delayed Mindful Senses Program (week 9-12) (45)	4, 8, (12, 16)	Thai HADS-D	Thai HADS-A
Luo (2021)	China	Parents of children diagnosed with cancer	33.60 (5.2)	69.9	device–based resilience	Nonspecific	General mental health	device–based resilience (52)	Information resources (51)	8, 24	SDS	-
Lüdtke (2018)	Germany	Adults with a subjective need for an intervention to reduce depressive symptoms	42.89 (11.19)	78.4	Be Good to Yourself	Multidisciplinary	Depression	Be Good to Yourself (45)	Waitlist (45)	4	PHQ9	-
MacKinnon (2022)	Canada	Mothers of preschool children (aged 18–36 months old) with moderate to severe depression	33.84 (5.34)	100	BEAM	Nonspecific	General mental health	BEAM (33)	Usual care (32)	10	PHQ9	GAD7
Mak (2018)	China	Adults in the general population	33.64 (12.08)	72.9	MBP, SCP, CBP	Mindfulness, Self-compassion, CBT	General mental health	MBP (739), SCP (748), CBP (795)	NA	4, 12	ACS	ACS
Mantani (2017)	Japan	Antidepressant-resistant adults with a primary diagnosis of major depressive disorder	40.90 (8.7)	53.4	Kokoro App	CBT	Depression	Kokoro App (81)	Usual care (83)	9, 17	PHQ9; BDI-2	-
Marciniak (2023)	Switzerland	University students with lowered reward sensitivity scores	21.50 (2.3)	80.0	Imager App Group	Nonspecific	General mental health	Imager App Group (51)	No intervention (44)	1	BDI-2	STAI
McCloud (2020)	United Kingdom	University students	24.3 (6.76)	85.1	Feel Stress Free App	CBT	General mental health	Feel Stress Free App (84)	Waitlist (84)	2, 4, 6	HADS-D	HADS-A
McGillivray (2023)	Australia	Adults between 18-25 years with suicidal thoughts the past year	21.5	84.6	LifeBuoy	Multidisciplinary	Suicidal ideation	LifeBuoy (228)	Education program (227)	6.14, 18.86	PHQ9	GAD7
Miklowitz (2023)	United States	Youth with bipolar and depressive disorders	15.8 (1.6)	72.3	My Coach-connect	Nonspecific	General mental health	My Coach-connect (32)	Placebo App (33)	9, 18, 27	-	SCARED
Min (2023)	South Korea	Hospital employees with elevated stress levels	38.64 (10.87)	90.2	Neuro-feedback assessed mindfulness ; mindfulness only	Mindfulness	Stress	Neuro-feedback assessed mindfulness (30); mindfulness only (33)	Education program (31)	4, 8	PHQ9	-
Mistretta (2018)	United States	Health care workers working at Mayo Clinic in Arizona	46 (12.6)	87	MBRT Int. (Mindfulness-based)	Mindfulness; Nonspecific	General mental health	MBRT Int. (Mindfulness-based) (22); Smartphone resilience int. (23)	Control group: self-monitoring (15)	6, 18	DASS-D	DASS-A
Moberg (2019)	United States	Adults scoring between 5-14 on the PHQ-8 or between 5-14 on the GAD7	30.20	54.8	Pacifica	Multidisciplinary	General mental health	Pacifica (253)	Waitlist (247)	4, 12	DASS-21, PHQ8	DASS-21, GAD7
Moritz (2024)	Germany	Adults aged between 18 and 85 years	30.45 (10.09)	84.5	COGITO App	Nonspecific	General mental health	COGITO App (108)	Waitlist (105)	6	PHQ9	GAD7

Mutter (2023)	Germany	University students struggling with procrastination	26.21 (5.3)	60.0	StudiCare Procrastionation	CBT	Procrastionation	StudiCare Procrastionation (116)	E-coach CBT (117)	4, 8, 12	PHQ8	GAD7
Newman (2021)	United States	Undergraduate students with self-reported GAD	21.40	77.0	self-help mobile program	CBT	General mental health	self-help mobile program (50)	No intervention (50)	12, 26	-	STAI-T
Nicol (2022)	United States	Adolescents aged 13 to 17 years with moderate depressive symptoms	14.7 (1.7)	88.2	WGenZ (Woebot-based application)	Multidisciplinary	General mental health	WGenZ (10)	Waitlist (8)	Depression: 2, 4, 6, 8. 12<br><br>Anxiety: 4, 8, 12	PHQ9	GAD7
Nishi (2022)	Japan	Pregnant women at 16–20 weeks' gestation, aged ≥ 20	30.44 (4.6)	100	Luna Baby app	CBT	General mental health	iCBT - Luna Baby app (2509)	No intervention (2508)	16, 22, 33	EPDS	-
Oh (2020)	Korea	Adult patients with mild-to-severe panic symptoms	41 (11.55)	51.2	*Todaki* Chatbot	CBT	Panic disorder	*Todaki* Chatbot (23)	Information resources (22)	4	HADS-D	HADS-A
Orosa-Duarte (2021)	Spain	University students	23.00 (4.16)	84.5	REM Volver a casa, IMBP group	Mindfulness		REM Volver a casa (54), IMBP group (51)	No intervention (49)	8	-	STAI-T
O'dea (2020)	Australia	Adolescents aged 12-16	14.82 (0.93)	86.5	WeClick	CBT	General mental health	WeClick (98)	Waitlist (95)	4, 12	PHQ-A	SCAS
O'Toole (2019)	Denmark	Individuals referred to out-patient suicide prevention treatment	28.75 (9.47)	41.9	LifeApp'tite	Nonspecific	Suicide risk	Usual care and LifeApp'tite (60)	Usual care (69)	8, 16	MDI	-
Peake (2024)	United States	Adolescents aged 13 to 21 years with depression symptoms	16.84 (2.55)	63.1	Spark app	CBT	Depression	Spark app (74)	Placebo app (79)	1, 2, 3, 4, 5	PHQ8	GAD7
Pham (2016)	United Kingdom	Adults with at least moderate anxiety symptoms	-	49.1	Flowy App	Mindfulness	Anxiety	Flowy App (31)	Waitlist (32)	2, 4	-	GAD7
Ponzo (2020)	United Kingdom	University students	19.96	65.9	Biobase app	Multidisciplinary	General mental health	Biobase app (59)	Waitlist Group (64)	4, 6	PHQ9, DASS-21	STAI
Possemato (2016)	United States	VA primary care veterans with PTSD symptoms	42 (12)	80	PTSD Coach	CBT	PTSD symptoms	PTSD Coach (Clinician support) (10)	PTSD Coach (Self-managing) (10)	8	PHQ9	-
Possemato (2023)	United States	Veterans with PTSD symptoms	50.92 (15.43)	9.8	Clinician supported PTSD Coach	Nonspecific	PTSD	Clinician supported PTSD Coach (115)	Usual care (119)	8, 16, 24	PHQ9	-
Qin (2022)	China	Chinese-speaking women who recently gave birth at a public hospital in Shanghai	31.90 (3.62)	100	CareMom + Usual Care	CBT	Depression	CareMom + Usual Care (57)	Waitlist (55)	1, 2, 3, 4	EPDS	GAD7
Raevuori (2021)	Finland	Patients with clinical depression in a Finnish university student health service	25.10 (4.5)	73	MeruHealth	Multidisciplinary	Depression	MeruHealth + TAU (63)	TAU (61)	4, 8, 20, 32	PHQ9	GAD7
Rajabi Majd (2020)	Iran	Adults having an insomnia disorder who speak Persian	35.75 (5.79)	55.8	CBT-I App	Multidisciplinary	Insomnia	CBT-I App (156)	Information resources (156)	10, 18, 30	HADS-D	HADS-A
Reid (2011)	Australia	Patients with mild or severe mental health issues	18.10 (3.2)	72	Mobiletype	Multidisciplinary	General mental health	Mobiletype (69)	Placebo app (49)	3, 9	DASS-D	DASS-A
Riordan (2024)	United States	Undergraduate student with elevated anxiety and/or depression symptoms	20.17 (1.58)	77.8	Healthy Minds App	Multidisciplinary	General mental health	Healthy Minds App (massed) (176)	Healthy Minds App (spaced) (175)	2	PROMIS-D	PROMIS-A

Rocamora González (2022)	Spain	Patients newly diagnosed with colorectal cancer	35.4			Mindfulness	General mental health	Calm App (52)	Usual care (50)	-	HADS-D	HADS-A
Roepke (2015)	United States	Individuals with clinically significant depression (CES-D ≥ 16)	40.15 (12.40)	69.60	CBT/PPT SuperBetter, General SuperBetter	Multidisciplinary	General mental health	CBT/PPT SuperBetter (93), General SuperBetter (97)	Waitlist Group (93)	2, 4, 6	CESD	GAD7
Roncero (2019)	Spain	Psychology students	21.56 (6.15)	81.4	GGRO	CBT	OCD	Immediate use (iApp) (51)	Waitlist (dApp) (46)	2, 4	DASS-D	-
Roy (2021)	United States	Individuals with GAD	41.95	90.48	TAU + unwinding anxiety app	Mindfulness	Anxiety	TAU + unwinding anxiety app (28)	TAU (33)	4, 8	-	GAD7
Röhr (2021)	Germany	Syrian refugee adults with elevated posttraumatic stress symptoms	33.33 (11.20)	38.3	Sanadak	CBT	Posttraumatic stress	Sanadak (65)	Education program (68)	4, 16	PHQ9	GAD7
Sawyer (2019)	Australia	New mothers	31.66	100.0	eMums Plus	CBT	Depression	eMums Plus (54)	Usual care (57)	32, 48	EPDS	-
Schwob (2023)	United States	Adults with social anxiety disorder (SAD)	19.40 (0.64)	53.7	Imaginal Exposure	Exposure	General mental health	Imaginal Exposure (39)	Self-monitoring (43)	1, 4	-	SPDQ, SIAS
Seo (2022)	South Korea	New mothers with elevated depression symptoms (EPDS ≥ 9)	NA	100.0	Happy Mother App	CBT	General mental health	Happy Mother App (50)	Information resources (50)	8, 20	EPDS	-
Sharma (2022)	Canada	Psychiatric inpatients between 18-65 years	31.83 (8.0)	24.8	Mindshift CBT	CBT	Anxiety	Mindshift CBT (9)	Usual care (11)	4	PHQ9	GAD7
Smith (2020)	United States	Employees from a large technology corporation	33.2 (7.8)	55	Wearable-based treatment	Mindfulness	General mental health	Wearable-based treatment (107)	Waitlist (108)	4	MASQ-D	MASQ-A
Smith (2021)	United States	Obstetrics and gynecology patients during the COVID-19 pandemic	36.21 (11.3)	100	Meditation App (CALM)	Mindfulness	General mental health	Mediation App (CALM) (50)	Usual Care (51)	2, 4	HADS-D	HADS-A
Smith_b (2021)	United States	Students enrolled in their third semester of physician assistant (PA) school	NA	78.6	10% Happier	Mindfulness	General mental health	10% Happier(8)	Waitlist Group (8)	8	DASS-D	DASS-A
Soltani (2024)	Iran	Iranian patients with MDD	NA	76.6	Yara App	Nonspecific	General mental health	Yara App (32)	Usual Care (32)	12	-	STAI
Stiles-Shields (2019)	United States	Adults with moderate depressive symptoms	NA	NR	Boost Me; Thought Challanger	CBT	Depression	Boost Me (10); Thought Challanger (10)	Waitlist (10)	3, 6, 10	PHQ9	-
Stolz (2018)	Switzerland	Adults who speak German and are diagnosed with Social anxiety disorder	34.76	63	PC-based treatment; Mobile treatment	CBT	Social Anxiety	PC-based treatment (60); Mobile treatment (60)	Waitlist (30)	12, 24	BDI2	-
Sun (2021)	China	Pregnant adult women from an obstetrics clinic	29.91 (4.02)	100	Spirits Healing App	Mindfulness	Depression	Spirits Healing App (84)	Attention control group (84)	4, 8, 18	EPDS	GAD7
Sun (2022)	China	Chinese university students in quarantine	22.21 (2.67)	74	Mindfulness based mHealth	Mindfulness	General mental health	Mindfulness based mHealth (57)	Placebo app: Social support based mHealth (57)	4, 8	PHQ9	GAD7
Taylor (2022)	United Kingdom	Health care workers	40.53	83.2	HeadSpace	Mindfulness	General mental health	HeadSpace (1095)	MoodZone: Psychoeducation (1087)	6, 18	DASS-21	DASS-21

Taylor (2023)	United Kingdom	UK-based working adults experiencing mild to moderate depressive symptoms	36.90 (9.5)	54.8	Activate your mood; Mind your mood; Finding happiness	Behavioural activation; CBT; ACT	Depression	Activate your mood (102); Mind your mood (101); Finding happiness (100)	Waitlist (102)	3, 7	PHQ8	GAD7
Thabrew (2022)	New Zealand	New Zealand residents aged between 16 and 30 years	23.68 (3.81)	87.8	Whitu	Multidisciplinary	General mental health	Whitu (45)	Waitlist (45)	4, 12	CESD	GAD7
Tighe (2017)	Australia	Indigenous Australians aged 18–35 years	26.25 (8.13)	63.9	ibobbly App	ACT	General mental health	ibobbly App (31)	Waitlist and delayed ibobbly App (30)	6	PHQ9	-
Toh (2022)	Singapore	Undergraduate university students	22.50 (5.41)	71	Stress-coping	CBT	Psychological stress	Stress-coping (135)	Cooperation: Placebo app (129)	1, 4	PHQ9	GAD7
Torok (2022)	Australia	Young adults from Australia experiencing recent suicidal ideation, aged 18-25 years	21.50 (2.18)	85	LifeBuoy	DBT	Change in suicidal ideation	LifeBuoy (228)	LifeBuoy-C Control: Placebo app (227)	6, 18	PHQ9	GAD7
Tønning (2021)	Denmark	Adult patients with a diagnosis of unipolar depressive disorder	43.94	52.5	Smartphone-based CBT	Multidisciplinary	General mental health	Smartphone-based CBT (59)	Usual care (61)	12, 26	HDRS-17, HDRS-6, BADS, BDI, HAM-D6	-
van Aubel (2020)	Netherlands	Individuals aged 16 to 25 with subthreshold depressive and/or psychotic complaints	21.01	72.7	ACT-DL Con.	ACT	General mental health	ACT-DL Con. (27)	FILM Cond. : Group treatment (28)	6, 26, 52	MADRS, IDS-SR, SCL-D	STAI-T, SCL-A
van Stolk-Cooke (2023)	United States	Adult family members of veterans with PTSD	39 (8.44)	97	PTSD Family Coach	Mutlidisciplinary	General mental health	PTSD Family Coach (104)	Psychoeducation-only app (96)	4	PHQ9	GAD7
Vereschagin (2024)	Canada	University students	20	70.3	Minder App	Multidisciplinary	General mental health	Minder App (743)	Waitlist (746)	4	PHQ9	GAD7
Versluis (2018)	Netherlands	Individuals reporting work stress	43.23 (11.39)	71	MovisensXS, VGZ mindfulness coach application	Multidisciplinary	General mental health	MovisensXS, worry-reduction training, VGZ mindfulness coach application (46)	Moodmonitoring (MovisensXS) (48)<br><br>Waitlist (42)	2, 4	PHQ9	GAD7
Vollert (2023)	Germany	individuals who wish to improve their sleep, age ≥18	37.30 (14.24)	62.8	Refresh	CBT	General mental health	Refresh (186)	Waitlist Group (185)	8, 24	PHQ9	-
Vàsquez (2023)	Spain	Caregivers with elevated depressive symptoms	50.0 (9.8)	92.6	CBIA	CBT	Depression	CBIA (58)	CBIA + conference call (54); Attention control group (63)	5	CES-D	-
Wang (2022)	China	Undergraduate nursing students	22.50 (1.50)	85.1	Intervention Group	Nonspecific	General mental health	Intervention Group (57)	Usual care (57)	8, 32	-	STAI
Watson-Singleton (2023)	United States	African Americans	36.06 (12.29)	54.1	BlackFULLness	Mindfulness	General mental health	BlackFULLness (84)	Waitlist (86)	12	DASS-21	DASS-21
Watts (2013)	Australia	Adults self identifying as suffering from mild to moderate depression	41 (12.38)	80	Get Happy	CBT	Depression	Get Happy- Mobile (22)	Get Happy- Computer (30)	8, 12	PHQ9; BDI2	-
WernerSeidler (2023)	Australia	Adolescents (ages 12–16) experiencing insomnia symptoms	14.71 (1.21)	71.3	SleepNinja	CBT	Insomnia	SleepNinja (131)	Active Control: Text message tips (133)	6, 14	PHQ-A	GAD7
Wilhelm (2022)	United States	Adults with body dysmorphic disorder	27 (9.6)	83.8	App-based CBT	CBT	Body Dysmorphic Disorder	App-based CBT (40)	Waitlist (40)	6, 12	QIDS-SR	-

Winslow (2022)	United States	Active duty military personnel with diagnosis or complaint of stress and anger	37.4 (7.7)	20	mHealth App	CBT	General mental health	mHealth App (10)	CBT alone (10); Asymptomatic group (10)	12	DASS-D	DASS-A
Wong (2021)	China	Hong Kong residents aged 18 years or older	35.69 (12)	77.4	Lifestyle Hub	Multidisciplinary	General mental health	Lifestyle Hub (53)	Waitlist (53)	8, 12	DASS-D	DASS-A
Yang (2023)	South Korea	Individuals with ASD aged 15–35 and had a total score of ≥39 on the STAI	20.97 (5.06)	10.0	HARU ASD	CBT	Anxiety symptoms	HARU ASD (15)	Waitlist Group (15)	9.43	-	STAI
Yoon (2022)	South Korea	Stressed employees	37.04 (9.25)	48.9	InMind App	Mindfulness	Stress	InMind App (22)	Waitlist (23)	4, 8	MBI	MBI
Zainal (2023)	United States	Clients with GAD and no treatment	20.80 (5.41)	85.5	Mindfulness EMI	Mindfulness	GAD	Mindfulness EMI (68)	Placebo app (42)	2, 4	-	GAD-Q-4-Dimensional
Zainal (2024)	Singapore	Adults with elevated levels of social anxiety symptoms	21.84 (3.37)	78	Mindfulness ecological momentary interventio (MEMI)	Mindfulness	Social anxiety disorder	MEMI (96)	Mood monitoring (95)	2, 6	BDI-2	GAD-Q-4
Zhang a (2023)	China	Women who were 12-20 weeks pregnant with psychological distress	30.29 (4,29)	100	Digital mindfulness-based intervention (Digital MBI)	Mindfulness	Maternal psychological distress	Digital mindfulness-based intervention (Digital MBI) (80)	No intervention (80)	8, 24, 30, 36, 48	EPDS	GAD7
Zhang b (2023)	China	Adults from China with insomnia	49.67 (14.49)	74	DCBT-I - Resleep	CBT	Insomnia	DCBT-I – Resleep (41)	Control group: information resources (41)	6, 10, 18, 30	PHQ9	GAD7
Zhao (2023)	China	Chinese individuals with elevated PTSD symptoms	25.12 (6.4)	75.6	ACT program	ACT	PTSD	ACT program (78)	Mindfulness (76)<br><br>Waitlist (67)	4	PHQ9	GAD7
Zhou (2023)	China	High school students in grades 10 and 11	16.80	43.5	Coping Camp	CBT	General mental health	Coping Camp (275)	No intervention (265)	11, 19	DASS-21	DASS-21

Note. Primary target: Primary target of the intervention. This can be different from the primary outcome if the intervention is primarily targeted to improve a different outcome than depression or anxiety (e.g., PTSD, insomnia, well-being). It was coded as "General mental health" if the primary target was not described. Technique: Primary therapeutic framework the intervention or control group is based on. Outcome measure questionnaire abbreviations: ACS = Affective Control Scale; ADS = General Depression Scale; BADS = Behavioral Activation for Depression Scale; BAI = Beck Anxiety Inventory; BDI = Beck Depression Inventory; BDI-2 = Beck Depression Inventory-2; BDI-FS = Beck Depression Inventory Fast Screen; BSI-A = Brief Symptom Inventory - Anxiety subscale; BSI-D = Brief Symptom Inventory - Depression subscale; CALS = Child and Adolescent Life Satisfaction Scale; CAPE = Community Assessment of Psychic Experiences; CCAPS-Depression = Counseling Center Assessment of Psychological Symptoms 34-item version - Depression subscale; CCAPS-General Anxiety = Counseling Center Assessment of Psychological Symptoms 34-item version - General Anxiety subscale; CCAPS-Social Anxiety = Counseling Center Assessment of Psychological Symptoms 34-item version - Social Anxiety subscale; CDI = Children's Depression Inventory; CDRS-R = Child Depression Rating Scale-Revised; CDS = Calgary Depression Scale; CES-D = Center for Epidemiological Studies Depression Scale; CGAS = Children's Global Assessment Scale; DASS-21 = The Depression Anxiety Stress Scales – 21; DASS-A = Depression, Anxiety, Stress Scale - Anxiety subscale; DASS-D = Depression, Anxiety, Stress Scale - Depression subscale; EPDS = Edinburgh Postnatal Depression Scale; GAD2 = Generalized Anxiety Disorder-2; GAD7 = Generalized Anxiety Disorder-7; GAD-Q-4-Dimensional = Generalised Anxiety Dimensional; GADQ-4 = Generalized Anxiety Disorder Questionnaire – fourth edition; HAM-A = Hamilton Anxiety Rating Scale; HAM-D6 = Hamilton Depression Self-rating Scale 6-item; HADS-A = Hospital Anxiety and Depression Scale - Anxiety subscale; HADS-D = Hospital Anxiety and Depression Scale - Depression subscale; HRSD-17 = Hamilton Rating Scale for Depression-17; IDS-SR = Inventory of Depressive Symptomatology, Self-Report; LSAS-SR = Liebowitz Social Anxiety Scale; MADRS = Montgomery Asberg Depression Rating Scale; MASQ-A = Mood and Anxiety Symptoms Questionnaire; MBI = Mibeong Index; MFQ-Ps = Mood and Feelings Questionnaire - Short Parent Version; MASC = Multidimensional Anxiety Scale for Children; Mini-SPIN = Social Phobia Inventory; PHQ2 = Patient Health Questionnaire-2; PHQ4 = Patient Health Questionnaire–4; PHQ8 = Patient Health Questionnaire-8; PHQ9 = Patient Health Questionnaire-9; PHQ-A = The Patient Health Questionnaire for Adolescents; PROMIS-A = Patient-Reported Outcomes Measurement Information System Anxiety; PROMIS-D = Patient-Reported Outcomes Measurement Information System Depression; PSRs = Psychiatric Status Ratings; QIDS-C = Quick Inventory of Depressive Symptomatology; QIDS-SR = Quick Inventory of Depressive Symptomatology - Self Report; RADS2 = Reynold's Adolescent Depression Rating Scale - 2nd Edition; SAS-A = Social Anxiety Scale; SCARED = Screen for Child Anxiety and Related Emotional Disorders (subscale generalised anxiety only); SCAS = Spence Children's Anxiety Scale; SCL-A = Symptom Checklist-90-R - Anxiety subscale; SCL-D = Symptom Checklist-90-R - Depression subscale; SIAS = Social Interaction Anxiety Scale; SDS = The Self-

Rating Depression Scale; SOPHS = Social Phobia Screener Scale; SPDQ = Social Phobia Diagnostic Questionnaire; STAI = State-Trait Anxiety Inventory; STAI-S = The State–Trait Anxiety Inventory - State anxiety subscale; STAI-T = The State–Trait Anxiety Inventory - Trait anxiety subscale; STAI-X2 = The situation-dependent trait version of State-Trait Anxiety Inventory; TMAS = Taylor Manifest Anxiety Scale.

Table S4. Risk of bias assessment on trial level

Author	D1 Inclusion/exclusion	D2 random sequence generation	D3 allocation concealment	D4 blinding of participants or personnel	D5 blinding of outcome assessment	D6 Completeness of outcome data	D7 Description of drop-out
Miklowitz	Low	Low	High	High	Low	Low	Low
Dahne (a)	Low	Low	High	High	Low	High	High
Smith a	Low	Low	High	High	Low	Low	High
He	Low	Low	Low	Low	Low	Low	Low
Graham	Low	Low	Low	High	Low	Low	Low
Al-Refae	Low	Low	High	High	Low	High	High
Zhang	Low	Low	High	High	Low	Low	Unclear
Hwang	High	Low	High	High	Low	High	Unclear
Marciniak	Low	Unclear	Unclear	High	Low	High	Low
Wang	Low	Low	High	Low	Low	High	High
Torok	Low	Low	Low	Low	Low	Low	Low
Yang	Low	Low	High	High	High	Low	Low
Boettcher	Low	Low	Unclear	High	Low	Low	Unclear
Sun	Low	Low	Low	Low	Low	Low	Low
Sawyer	Low	Low	Low	High	Low	Low	Unclear
Abbasalizadeh	Low	Unclear	Unclear	High	Low	Low	Low
Zhang b	Low	Low	Low	Low	Low	Unclear	Unclear
Mistretta	Low	Unclear	High	Low	Low	Unclear	Low
Litvin	Low	Low	High	Low	Low	High	Unclear
Roy	Low	Low	Low	High	Low	Unclear	Low
Jannati	Low	Low	High	Low	Low	Low	Low
Lim	Low	Low	High	High	Low	High	High

Luo	Low	Low	Low	Low	Low	Low	Low
Roncero	Low	Low	High	High	Low	Low	High
Goldberg	Low	Unclear	High	High	Low	Low	Unclear
Raevuori	Low	Low	Low	Low	Low	Low	Unclear
Toh	Low	Low	High	Low	Low	High	Unclear
Soltani	Low	Low	High	High	Low	High	Low
Kuhn	Low	Low	High	High	Low	Low	Low
El-Jawahri	Low	Low	Unclear	High	Low	Low	Low
DiNardo	Low	Unclear	High	Low	Low	Low	Low
Kubo	Low	Low	Low	High	Low	Low	High
Hanssen	Low	Unclear	High	High	High	High	High
Ahorsu	Low	Low	Low	Low	Low	Low	Unclear
Liu	Low	Low	Low	High	Low	Unclear	Unclear
Possemato	Low	Unclear	High	High	Low	Low	Low
Ben-Zeev	Low	Low	Low	Low	Low	Low	Unclear
Imamura	Low	Low	Low	Low	Low	Low	Low
Bakker	High	Unclear	High	High	Low	Unclear	Low
Kloos	Low	Low	High	High	Low	Low	Low
Flett	Low	Low	High	High	Low	Unclear	Unclear
Forman-Hoffman	Low	Low	High	High	Low	Low	Unclear
Sharma	Low	Low	High	High	Low	High	Low
van Stolk-Cooke	Low	Unclear	High	High	Low	Low	High
Catuara-Solarz	Low	Low	Low	High	Low	High	Low
Dahne_b	Low	Unclear	High	High	Low	High	Low
Howells	Low	Low	High	Low	Low	High	High
Kerber	Low	Low	High	High	Low	Low	High
J√§rvel√§-Reijonen	Low	Low	High	High	Low	Unclear	Unclear
Bostock	Low	Low	High	High	Low	Unclear	Low

Aboody	Low	Low	High	High	Low	Low	Low
Abramovitch	Low	Unclear	High	High	Low	Low	Low
Bruhns	Low	Low	High	High	Low	Low	Low
Deady	Low	Low	Low	High	Low	High	High
Ghaemi	Low	Low	Unclear	Low	Low	Low	Low
Birney	Low	Unclear	High	Low	Low	Low	High
Ditton	Low	Unclear	High	Low	Low	Low	Low
Stolz	Low	Low	Low	Low	Low	Unclear	High
Sun	Low	Low	Low	Unclear	Low	Low	Unclear
Deady	Low	Low	High	Low	Low	Low	Unclear
Greer	Low	Low	High	High	Low	Low	Low
Hunt	Low	Unclear	High	High	Low	Low	High
Ponzo	Low	Unclear	High	High	Low	High	Unclear
Nishi	Low	Low	Low	Low	Low	Low	High
Watson-Singleton	High	Low	High	Low	Low	High	High
Everitt	Low	Low	High	High	Low	High	High
Carl	Low	Low	Low	Low	Low	Low	Unclear
Anastasiadou	Low	Low	Unclear	High	Low	Low	Unclear
O'Toole	Low	Unclear	High	High	Low	Low	High
Guo	Low	Low	High	High	Low	Low	Low
Thabrew	Low	Unclear	High	High	Low	Unclear	Low
McCloud	Low	Low	High	High	Low	Low	Unclear
RocamoraGonzalez	Low	Low	High	Low	Low	Low	Low
Ghanbari	Low	Low	Unclear	High	Low	Unclear	Low
Donker	Low	Low	Low	Low	Low	Low	Low
LooGee	Low	Low	High	High	Low	Low	Unclear
Winslow	Low	Unclear	High	High	Low	Unclear	High
Huberty	Low	Low	High	High	Low	Low	Unclear

Zhao	Low	Low	Low	Low	Low	Low	High
Hwang	Low	Low	Low	High	Low	Low	Low
Kim	Low	Low	High	High	High	Low	Low
Versluis	Low	Low	Low	High	Low	Low	Unclear
De Kock	Low	Low	Low	Low	Low	Low	Unclear
Fiol-De Roque	Low	Low	Low	Low	Low	Low	Low
Vollert	Low	Unclear	Low	High	Low	Low	High
Fuller-Tyszkiewicz	Low	Low	High	Low	Low	Low	High
Dingwall	Low	Low	Low	Low	Low	Low	Low
Birrell	High	Low	Low	Low	Low	Unclear	Low
Laird	Low	Low	High	High	Low	High	Unclear
Vereschagin	Low	Low	High	High	Low	Low	Unclear
Hilt	Low	Unclear	High	High	Low	Low	Low
Keng	Low	Low	High	High	Low	Low	Low
Tighe	Low	Low	High	High	Low	Low	Low
Ludtke	Low	Unclear	Low	High	Low	Low	Low
Heim	Low	Low	High	Low	Low	Low	Low
Luangapichart	Low	Low	Low	High	Low	Low	Low
Oh	Low	Unclear	High	High	Low	Unclear	Low
Danieli	Low	Low	High	High	Low	Unclear	Low
Yoon	Low	Low	Unclear	High	Low	Low	Low
Vasquez	Low	Low	Low	Low	Unclear	Low	Low
Qin	Low	Low	High	High	Low	Unclear	Unclear
Li	Low	Low	High	Low	Low	Low	Low
Ham	Low	Unclear	High	Low	Low	Unclear	Low
McGillivray	Low	Low	Low	Low	Low	Low	Low
R√∂hr	Low	Low	Low	Low	Low	Low	Low
Kauer	Low	Low	Unclear	Low	Low	Low	Low

Depp	Low	Low	Low	Low	Low	High	Unclear
Hur	Low	Low	High	High	Low	High	High
Werner,ÄêSeidler	Low	Low	Low	Low	Low	Low	High
Chan	Low	Unclear	High	Unclear	High	Low	Unclear
Wong	Low	Low	Low	High	Low	Low	Low
Ben-Zeev	Low	Low	Low	High	Low	Low	Low
Wilhelm	Low	Unclear	High	Low	Low	Low	Low
Br√∂cker	Low	Low	Low	Low	Low	Low	Low
Levin	Low	Low	High	Low	Low	Low	High
Grubbs	Low	Low	High	High	Low	Low	High
Lacey	Low	Low	High	High	Low	Low	Low
Li	Low	Unclear	High	High	Low	Unclear	Unclear
Orosa-Duarte	Low	Low	High	High	Low	Unclear	High
Zhou	Low	Low	Low	High	Low	Low	Unclear
Horsch	Low	Low	Low	Low	Low	Low	Unclear
Taylor	Low	Low	Low	Low	Low	Low	High
Cox	Low	Low	Unclear	High	Low	Unclear	Low
Kosasih	Low	Low	High	Low	Low	Low	Unclear
Seo	Low	Unclear	Low	Low	Low	Unclear	Low
Linardon	Low	Low	Low	Low	Low	Low	High
Gao	Low	Low	Low	Low	Low	Low	Low
Kollei	Low	Low	High	High	Low	Low	Unclear
Bruhns	Low	Low	High	High	Low	Low	Unclear
Smith_b	High	Low	Low	Low	Low	Low	Low
Roepke	Low	Low	High	High	Low	Low	Unclear
Kulikov	Low	Low	High	High	Low	Unclear	High
Schwob	Low	Unclear	Unclear	Low	Low	Low	Low
Liu	Low	Low	High	High	Low	Low	Unclear

Gao	Low	Low	Low	High	Low	Low	Unclear
Krafft	Low	Unclear	High	High	Low	Low	Unclear
Litvin	Low	Low	High	High	Low	Low	Unclear
Linardon	Low	Low	Low	High	Low	Low	Low
Economides	Low	Low	Low	High	Low	Low	Low
Domar	Low	Low	Low	High	Low	Low	Low
Bruehlman-Senecal	Low	Low	High	High	Low	High	Unclear
Borosund	Low	Low	High	High	Low	Low	Unclear
Bell	Low	Low	Low	High	Low	Low	Low
Kuhn	Low	Low	Low	High	Low	Low	Low
Moritz	Low	Low	High	High	Low	Low	Unclear
Comtois	Low	Low	High	Unclear	Low	Low	Unclear
Donker	Low	Low	Low	Low	Low	Low	Low
Lee	Low	Low	Low	High	Low	Low	Unclear
Ben-Zeev	Low	Low	High	High	Low	Low	Unclear
Huberty	Low	Low	Low	High	Low	Low	Unclear
Pham	Low	Low	High	High	Low	Low	High
LaFreniere	High	Low	High	High	Low	Low	Low
Taylor	Low	Low	Unclear	High	Low	Low	Unclear
Mantani	Low	Low	Low	High	Low	Low	Low
Carli	Low	Low	Low	High	Low	Low	Low
Kubo	Low	Low	Unclear	High	Low	Low	Low
Akechi	Low	Low	Low	High	Low	Low	Low
Gnanapragasam	Low	Low	Low	Low	Low	Low	Low
Kirykowicz	Low	Unclear	High	High	Low	Unclear	Unclear
Abboot	Low	Unclear	High	High	Low	Low	High
Riordan	Low	Unclear	High	High	Low	Unclear	Unclear
Min	Low	Low	Low	High	Low	Unclear	Unclear

Zainal	Low	Low	Low	Low	Low	Low	High
Mutter	Low	Low	Low	High	Low	Low	Unclear
Levin	Low	Low	High	High	Low	Low	Unclear
Tonning	Low	Low	Low	Low	Low	Low	Low
Cerea	Low	Low	High	High	Low	Unclear	Low
Lu	Low	Unclear	High	High	Low	Low	Unclear
Moberg	Low	Unclear	High	High	Low	Low	High
van Aubel	Low	Low	High	Low	Low	Unclear	Unclear
Mak	Low	Low	High	High	Low	Low	Unclear
Newman	Low	Low	Low	High	Low	Unclear	Unclear
Cox	Low	Unclear	Unclear	High	Low	Unclear	Low
Cerea	Low	Low	High	High	Low	Low	Low
Barroso	Low	Low	High	High	Low	Low	Low
Bear	Low	Unclear	High	High	Low	Unclear	High
Borjalilu	Low	Unclear	High	High	Low	High	High
Borosund	Low	Low	High	High	Low	Low	Low
Cardi	Low	Low	High	High	Low	Unclear	High
Watts	Low	Low	Low	High	Low	Low	Low
Lewis	Low	Low	High	High	Low	Low	Low
Li_dysregulated	Low	Low	Low	High	Low	Low	High
Liu_b	Low	Low	High	Low	Low	Low	High
MacKinnon	Low	Low	Low	Low	Low	Low	Unclear
Nicol	Low	Low	High	High	High	Unclear	Unclear
Zainal	Low	Low	Low	Low	Low	Low	Unclear
Faurholt-Jepsen	Low	Low	Low	Low	Low	Low	Low
Stiles-Shields	Low	Low	Low	High	Low	High	Low
Reid	Low	Low	Low	Low	Low	Low	Low
Rajabi Majd	Low	Low	Low	High	Low	Low	Low

Possemato	Low	Unclear	Low	High	Low	Low	Low
Hirshberg	Low	Low	Low	High	Low	Low	Unclear
Smith	High	Unclear	High	High	Low	High	Unclear
Fish	Low	Unclear	High	High	Low	High	Low
Fitzpatrick	Low	Low	Low	High	Low	Low	Unclear
Ha	High	Unclear	High	High	Low	Unclear	Low
Hensler	Low	Low	Low	High	Low	Low	Low
Lahtinen	Low	Low	High	High	Low	Unclear	High
Lee	Low	Low	High	High	Low	Unclear	Low
Levin_b	Low	Low	High	High	Low	NA	NA
Araya_Brazil	Low	Low	Low	Low	Low	Unclear	Low
Peake	Low	Low	High	High	Low	Low	Unclear
Bostock	Low	Low	Low	High	Low	Low	High
Kusumadewi	Low	Unclear	High	High	Low	Unclear	Low
Kenny	Low	Low	High	High	Low	Low	High
O'Dea	Low	Low	Low	High	Low	Low	Unclear

Figure S1. Correlation matrix

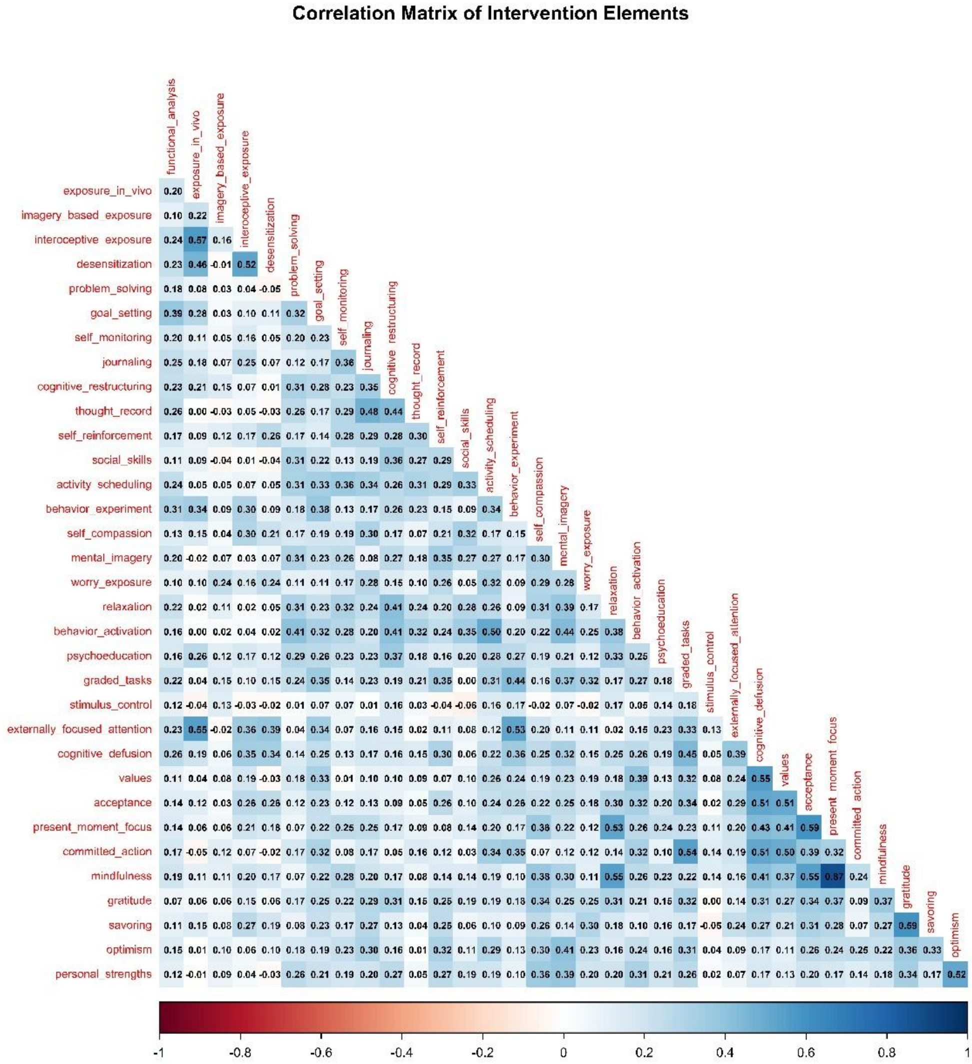

Figure S2. Risk of bias across all trials included in the systematic review

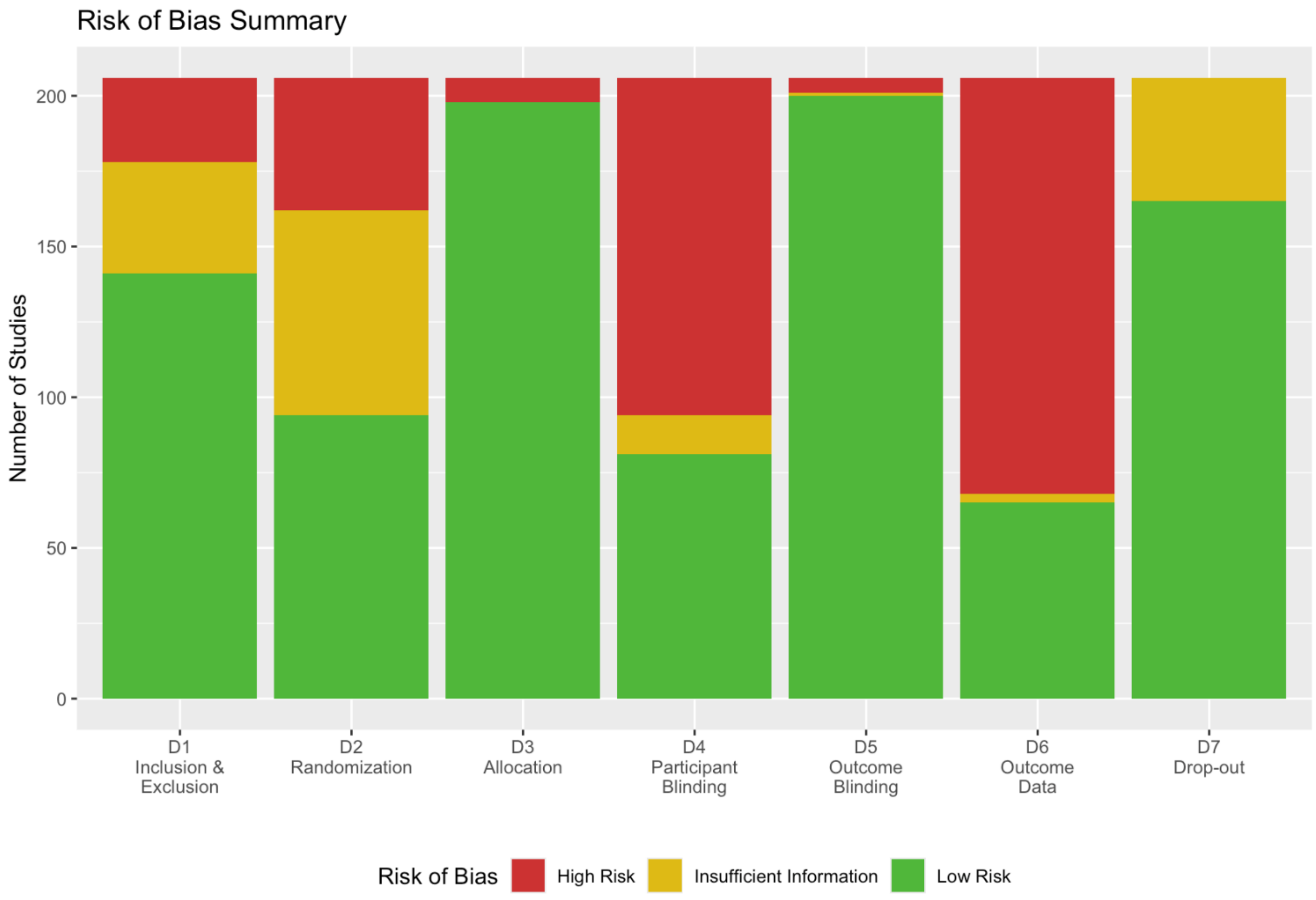

Table S5. PRISMA reporting checklist.

Section and Topic	Item #	Checklist item	Location where item is reported
**TITLE**			
Title	1	Identify the report as a systematic review.	1
**ABSTRACT**			
Abstract	2	See the PRISMA 2020 for Abstracts checklist.	2
**INTRODUCTION**			
Rationale	3	Describe the rationale for the review in the context of existing knowledge.	3-4
Objectives	4	Provide an explicit statement of the objective(s) or question(s) the review addresses.	4-5
**METHODS**			
Eligibility criteria	5	Specify the inclusion and exclusion criteria for the review and how studies were grouped for the syntheses.	16
Information sources	6	Specify all databases, registers, websites, organisations, reference lists and other sources searched or consulted to identify studies. Specify the date when each source was last searched or consulted.	17
Search strategy	7	Present the full search strategies for all databases, registers and websites, including any filters and limits used.	18, review protocol on OSF
Selection process	8	Specify the methods used to decide whether a study met the inclusion criteria of the review, including how many reviewers screened each record and each report retrieved, whether they worked independently, and if applicable, details of automation tools used in the process.	17
Data collection process	9	Specify the methods used to collect data from reports, including how many reviewers collected data from each report, whether they worked independently, any processes for obtaining or confirming data from study investigators, and if applicable, details of automation tools used in the process.	17-19
Data items	10a	List and define all outcomes for which data were sought. Specify whether all results that were compatible with each outcome domain in each study were sought (e.g. for all measures, time points, analyses), and if not, the methods used to decide which results to collect.	17-19
	10b	List and define all other variables for which data were sought (e.g. participant and intervention characteristics, funding sources). Describe any assumptions made about any missing or unclear information.	17-19
Study risk of bias assessment	11	Specify the methods used to assess risk of bias in the included studies, including details of the tool(s) used, how many reviewers assessed each study and whether they worked independently, and if applicable, details of automation tools used in the process.	19-20
Effect measures	12	Specify for each outcome the effect measure(s) (e.g. risk ratio, mean difference) used in the synthesis or presentation of results.	20
Synthesis methods	13a	Describe the processes used to decide which studies were eligible for each synthesis (e.g. tabulating the study intervention characteristics and comparing against the planned groups for each synthesis (item #5)).	20-21
	13b	Describe any methods required to prepare the data for presentation or synthesis, such as handling of missing summary statistics, or data conversions.	20-21

Section and Topic	Item #	Checklist item	Location where item is reported
	13c	Describe any methods used to tabulate or visually display results of individual studies and syntheses.	20-21
	13d	Describe any methods used to synthesize results and provide a rationale for the choice(s). If meta-analysis was performed, describe the model(s), method(s) to identify the presence and extent of statistical heterogeneity, and software package(s) used.	20-21
	13e	Describe any methods used to explore possible causes of heterogeneity among study results (e.g. subgroup analysis, meta-regression).	20-21
	13f	Describe any sensitivity analyses conducted to assess robustness of the synthesized results.	20-21
Reporting bias assessment	14	Describe any methods used to assess risk of bias due to missing results in a synthesis (arising from reporting biases).	Not conducted
Certainty assessment	15	Describe any methods used to assess certainty (or confidence) in the body of evidence for an outcome.	Not conducted
**RESULTS**			
Study selection	16a	Describe the results of the search and selection process, from the number of records identified in the search to the number of studies included in the review, ideally using a flow diagram.	5, Figure 1
	16b	Cite studies that might appear to meet the inclusion criteria, but which were excluded, and explain why they were excluded.	5, Figure 1
Study characteristics	17	Cite each included study and present its characteristics.	Supporting information (S2, Table S3)
Risk of bias in studies	18	Present assessments of risk of bias for each included study.	Supporting information (Table S4, Figure S2)
Results of individual studies	19	For all outcomes, present, for each study: (a) summary statistics for each group (where appropriate) and (b) an effect estimate and its precision (e.g. confidence/credible interval), ideally using structured tables or plots.	7-8, Table 3, Table 4
Results of syntheses	20a	For each synthesis, briefly summarise the characteristics and risk of bias among contributing studies.	5-6
	20b	Present results of all statistical syntheses conducted. If meta-analysis was done, present for each the summary estimate and its precision (e.g. confidence/credible interval) and measures of statistical heterogeneity. If comparing groups, describe the direction of the effect.	7-8, Figure 2, Table 3, Table 4, Figure 3
	20c	Present results of all investigations of possible causes of heterogeneity among study results.	7-8
	20d	Present results of all sensitivity analyses conducted to assess the robustness of the synthesized results.	7-8, Output of sensitivity analyses on OSF

Section and Topic	Item #	Checklist item	Location where item is reported
Reporting biases	21	Present assessments of risk of bias due to missing results (arising from reporting biases) for each synthesis assessed.	Not conducted
Certainty of evidence	22	Present assessments of certainty (or confidence) in the body of evidence for each outcome assessed.	Not conducted
**DISCUSSION**			
Discussion	23a	Provide a general interpretation of the results in the context of other evidence.	9-14
	23b	Discuss any limitations of the evidence included in the review.	15
	23c	Discuss any limitations of the review processes used.	15
	23d	Discuss implications of the results for practice, policy, and future research.	9-14
**OTHER INFORMATION**			
Registration and protocol	24a	Provide registration information for the review, including register name and registration number, or state that the review was not registered.	16
	24b	Indicate where the review protocol can be accessed, or state that a protocol was not prepared.	16
	24c	Describe and explain any amendments to information provided at registration or in the protocol.	19
Support	25	Describe sources of financial or non-financial support for the review, and the role of the funders or sponsors in the review.	22
Competing interests	26	Declare any competing interests of review authors.	22
Availability of data, code and other materials	27	Report which of the following are publicly available and where they can be found: template data collection forms; data extracted from included studies; data used for all analyses; analytic code; any other materials used in the review.	16, 21, 22
